# Regioselective ring expansion followed by H-shift of 3-ylidene oxindoles: a convenient synthesis of N-substituted/un-substituted pyrrolo[2,3-*c*] quinolines and marinoquinolines†

**DOI:** 10.1039/c9ra07831b

**Published:** 2019-10-30

**Authors:** Gopathi Ramu, Srinivas Ambala, Jagadeesh Babu Nanubolu, Bathini Nagendra Babu

**Affiliations:** Department of Fluoro-Agrochemicals, CSIR-Indian Institute of Chemical Technology Hyderabad 500 007 India; Academy of Scientific and Innovative Research (AcSIR) New Delhi 110025 India; Centre for X-ray Crystallography, CSIR-Indian Institute of Chemical Technology Hyderabad India bathini@iict.res.in

## Abstract

Herein, we report a simple and metal-free protocol for the synthesis of 4-oxo-4,5-dihydro-3*H*-pyrrolo[2,3-*c*]quinolines. The present method under mild reaction conditions with wide functional group compatibility gives several unexplored N-substituted/unsubstituted 4-oxo-4,5-dihydro-3*H*-pyrrolo[2,3-*c*]quinolines and marinoquinolines in good to excellent yields. Mechanistic insights for the synthesis of N-substituted pyrroloquinolines reveal the ring expansion of 3-ylideneoxindoles and H-shift as the key steps.

## Abstract 2

Herein, we report a simple and metal-free protocol for the synthesis of 4-oxo-4,5-dihydro-3*H*-pyrrolo[2,3-*c*]quinolines.

## Graphical abstract

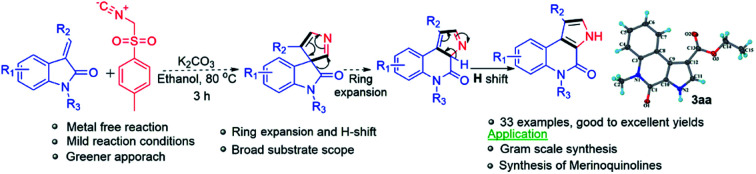

## Introduction

**Fig. 1:**
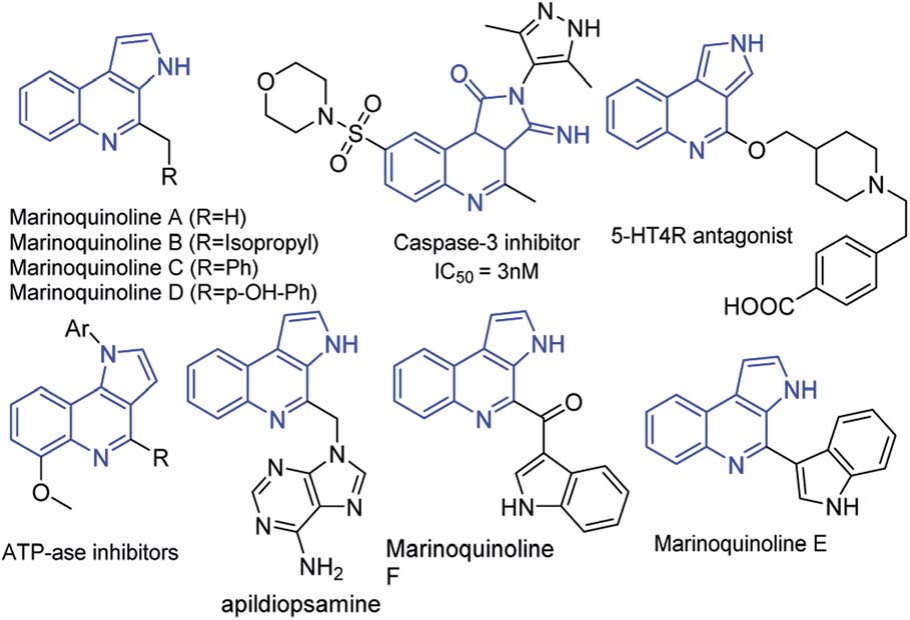
Examples of biologically important pyrroloquinoline derivatives.

Functionalized heteroarenes, particularly azaheteroarenes are indispensable structural units in a large number of natural products, pharmaceuticals, agrochemicals, and functional materials.^1*a*–*e*^ The fused azaheteroarenes such as pyrrolo[2,3-*c*]quinoline derivatives occupy an essential role, due to their wider occurrence in the pharmaceutically active compounds. These are very well explored in the literature for various medicinal applications such as a caspase 3-inhibitor,^2^ 5HT4R antagonist,^3^ ATPase inhibitor,^4^ apildiopsamine,^5^ and marinoquinoline A, E, and F.^6^ A few examples are shown (Fig. 1).

**Scheme 1:**
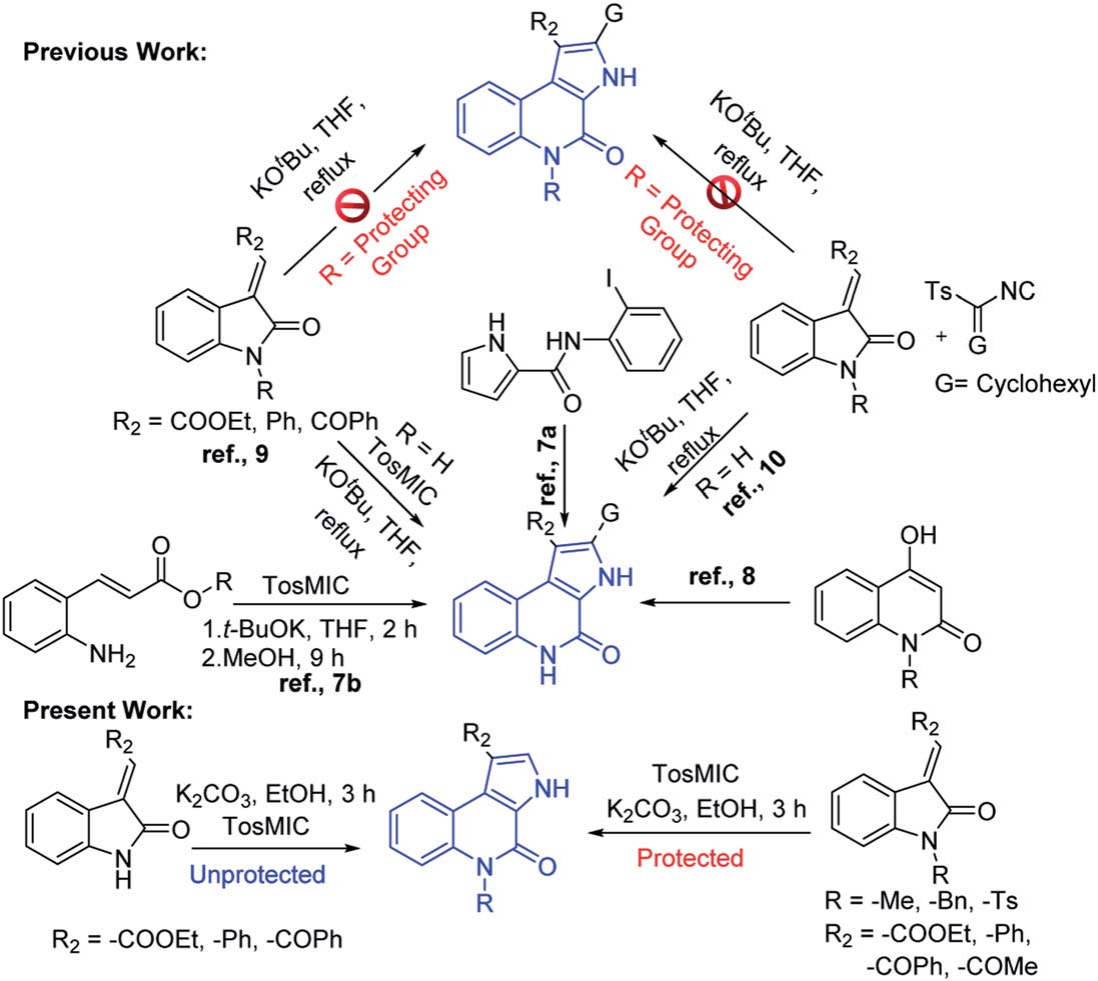
Comparison of our work with the previous reports for the synthesis of pyrroloquinolines.

Owing to its huge application in various scientific fields, the development of new synthetic methodologies for the synthesis of these compounds is of high demand and attractive in the synthetic community. In the recent past, several synthetic methods have been reported for the synthesis of fused pyrrolo[2,3-*c*]quinoline derivatives. In 2009, Rossi *et al.*, developed photostimulated intramolecular S_RN_1 reactions for 3*H*-pyrrolo[2,3-*c*]quinolin-4(5*H*)-one synthesis.^7^ Recently, palladium catalysed intramolecular cycloaddition was also explored.^8^ KO^*t*^Bu mediated synthesis of 3*H*-pyrrolo[2,3-*c*]quinolin-4(5*H*)-one was achieved by Bergman *et al.*,^9^ and Ji *et al.*,^10^ separately (Scheme 1). Synthesis of N-unsubstituted pyrrolo[2,3-*c*]quinoline, which in fact attempted by Bergman *et al.*, where, isocyanate intermediate formation and H-shift are the key steps in the mechanism and it is applied only for the synthesis of N-unsubstituted pyrrolo[2,3-*c*]quinolines. Unfortunately, to the best of our knowledge no successful attempts were reported for the synthesis of N-substituted fused pyrrolo[2,3-*c*]quinolines. More recently, in our previous work, we have developed methods for the synthesis of pyrazoloquinazolinones in a single step from isatin derivatives.^11^ Herein, we report the study on development of an effective method for the synthesis of N-unsubstituted as well as N-substituted pyrrolo[2,3-*c*]quinoline, which in fact was also attempted by Ji *et al.*, but it is applicable only for the synthesis of N-unsubstituted pyrrolo[2,3-*c*]quinolines.^10^ However, the synthesis of N-substituted pyrrolo[2,3-*c*]quinolines are not-feasible with this strategy as N–H cleavage is involved in the isocyanate intermediate formation. In this regard, herein our current approach, spiro ring expansion followed by H-shift are the crucial steps and offers both N-substituted and unsubstituted pyrrolo[2,3-*c*]quinolines.

## Results and discussion

**Scheme 2:**
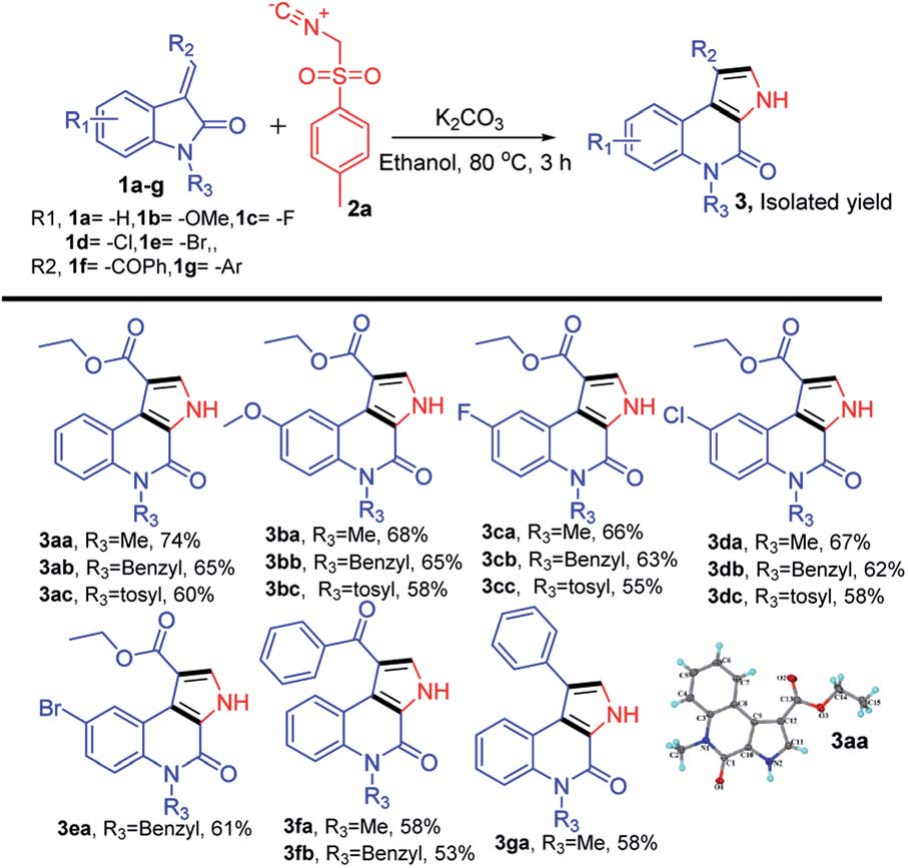
Substrate scope for synthesis of N-substituted-4-oxo-4,5-dihydro-3*H*-pyrrolo[2,3-*c*]quinolines.

We began our study by evaluating various conditions for the synthesis of ethyl 5-methyl-4-oxo-4,5-dihydro-3*H*-pyrrolo[2,3-*c*]quinoline-1-carboxylate 3aa, with the reaction of ethyl (*E*)-2-(1-methyl-2-oxoindolin-3-ylidene)acetate (1a) with TosMIC (2a) in the presence of K_2_CO_3_ in ethanol at room temperature. Disappointingly, no product formation was observed (Table 1, entry 1). Next, a series of experiments were conducted at different temperatures, to our delight, the best results, 65%, 74% and 67% of 3aa, were obtained at 60, 80 and 100 °C respectively (Table 1, entries 2–4). Afterwards, various solvents were screened (Table 1, entries 5–11), the use of polar solvents gave relatively better yields than the non-polar solvents, 74% and 70% of 3aa were obtained using ethanol and methanol as solvents. Subsequently, reactions were also tested with various inorganic and organic bases in ethanol at 80 °C, which revealed that K_2_CO_3_ was suitable among all (Table 1, entries 12–16). Therefore, we used K_2_CO_3_ (2 equiv.) in ethanol at 80 °C as the optimal reaction condition (Table 1, entry 3). After investigating the optimal reaction condition, we next focused our attention to the scope of this reaction by reacting various N-substituted, along with aryl substituted compounds 1a–g with TosMIC 2a and almost all the reactions underwent smoothly to the respective products from good to excellent yields (Scheme 2). Reaction of 1a (*R*_1_ = H; *R*_3_ = –Me, –Bn and –Tos) with TosMIC 2a underwent smooth reaction and afforded the corresponding products 3aa–3ac with 74, 65 and 60% yield respectively. Further, compounds substituted with electron donating group such as 1b (*R*_1_ = –OMe; *R*_3_ = –Me, –Bn and –Tos) when treated with TosMIC 2a, gave the required products in 68, 65, 58% yield 3ba–3bc. On the other hand, the substrates with electron withdrawing groups at 5th position (*R*_1_ = –F, –Cl, and –Br; *R*_3_ = –Me, –Bn and –Tos) 1c–e were tried for the reaction with TosMIC 2a, and produced the required products in moderate to good yields 3ca–3ga.

**Table 1:** Optimization of reaction conditions^a^

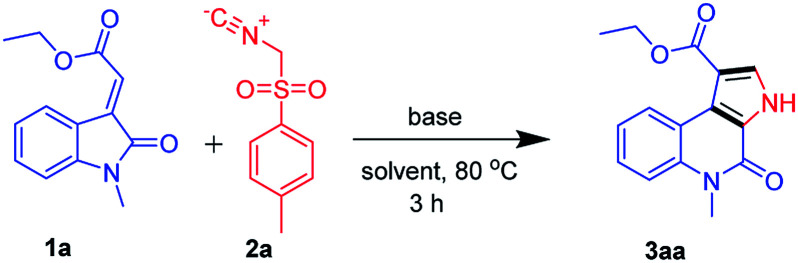				
Entry	Base	Solvent	Temp (°C)	Yield^b^ (%)
1	K_2_CO_3_	Ethanol	rt	—
2	K_2_CO_3_	Ethanol	60	65
3	K_2_CO_3_	Ethanol	80	74
4	K_2_CO_3_	Ethanol	100	67
5	K_2_CO_3_	Methanol	80	70
6	K_2_CO_3_	CH_3_CN	80	68
7	K_2_CO_3_	1-Propanol	80	62
8	K_2_CO_3_	Toluene	80	63
9	K_2_CO_3_	THF	40	60
10	K_2_CO_3_	CHCl_3_	60	52
11	K_2_CO_3_	DCE	80	60
12	Cs_2_CO_3_	Ethanol	80	72
13	Na_2_CO_3_	Ethanol	80	65
14	KO^*t*^Bu	Ethanol	80	52
15	Et_3_N	Ethanol	80	48
16	Pyridine	Ethanol	80	52

a Reaction conditions: 1a (0.5 mmol), 2a (0.5 mmol), base (1.0 mmol), solvent (5.0 mL).

b Isolated yields.

From the above results, there is no such general trend was observed with the substrates having substitution on the phenyl ring of (*E*)-2-(2-oxoindolin-3-ylidene)acetate 1a, whereas a remarkable impact on the yields was observed with the N-protected substrates. Electron donating groups on the N-atom produced the better yields than the electron withdrawing groups.

**Scheme 3:**
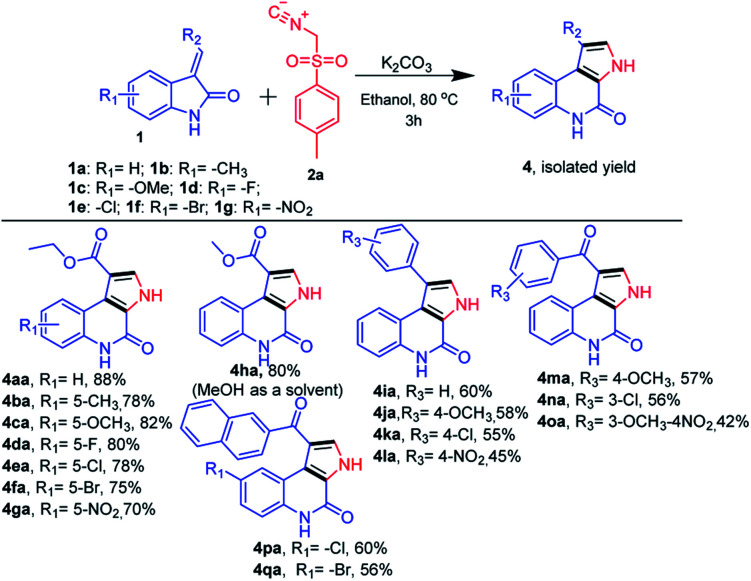
Substrate scope for synthesis of N-unsubstituted 4-oxo-4,5-dihydro-3*H*-pyrrolo[2,3-*c*]quinolines.

Next, we further extended the use of the present optimized base-mediated synthesis of ethyl 4-oxo-4,5-dihydro-3*H*-pyrrolo[2,3-*c*]quinoline-1-carboxylate 4aa to explore unprotected ethyl (*E*)-2-(2-oxoindolin-3-ylidene)acetate with TosMic 2a. We then examined the scope of the reaction using a variety of structurally diverse unprotected ethyl (*E*)-2-(2-oxoindolin-3-ylidene)acetates 1a precursors (Scheme 3). Further, a range of functional groups such as electron donating (*R*_1_ = –OMe, and –Me) as well as electron withdrawing groups on the phenyl ring of oxindole (*R*_1_ = –F, –Cl, –Br and –NO_2_) were also compatible with the reaction. These underwent the desired transformation efficiently and furnished the corresponding product (Scheme 3, 4aa–4ga) in good to excellent yields. It was found that various substitutions on the olefinic position of the oxindole (*R*_2_ = –CO_2_Et, –CO_2_Me, substituted aryl and phenacyl) are amenable to the present reaction and also afforded good to high yields (Scheme 3). It is to be noted that, the electronic nature of substituents on the olefinic position of (*E*)-2-(2-oxoindolin-3-ylidene)acetate has discernible impact on the reaction efficacy where electron donating (*R*_3_ = –substituted aryl) groups provided corresponding products in slightly better yields than the electron withdrawing groups (*R*_2_ = –substituted phenacyl) (Scheme 3, 4ha–qa).

**Scheme 4:**
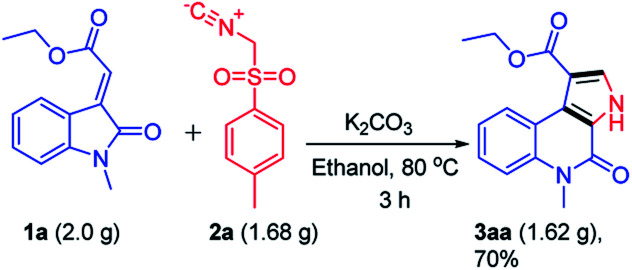
Gram scale synthesis of compound 3aa.

To further explore the synthetic utility of this reaction, a gram scale reaction was performed under optimized reaction conditions which delivered ethyl 5-methyl-4-oxo-4,5-dihydro-3*H*-pyrrolo[2,3-*c*]quinoline-1-carboxylate 3aa without affecting the reaction efficacy showing its potential in bulk scale utility (Scheme 4).

**Fig. 2:**
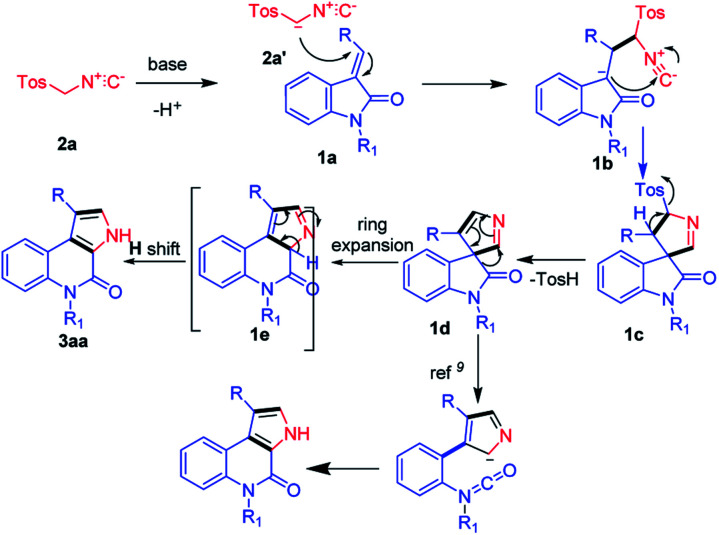
Plausible reaction mechanism for the synthesis of pyrrolo[2,3-*c*]quinolines.

With the aforementioned experimental results and the literature precedents, we propose a plausible reaction mechanism for the base-mediated synthesis of 4-oxo-4,5-dihydro-3*H*-pyrrolo[2,3-*c*]quinolines (Fig. 2). The reaction starts with, TosMIC 2a reacts with base K_2_CO_3_ and generates anionic intermediate 2a′. The generated anionic intermediate 2a′ participates in the 3 + 2 cycloaddition with (*E*)-2-(2-oxoindolin-3-ylidene)acetate 1a to form the corresponding intermediate 1b, which undergoes 5-membered spiro ring formation and generates 1c. Next, formation of 1d occurs with the elimination of TsOH. Here, in the literature, Isocyanate formation occurs with the –H-shift on the –N atom of 1d. Whereas the groups like –Me, –Bn, –Tos shift could be a tedious process and difficult to form isocyanate intermediate.^9^ In our current strategy, 1d undergoes ring expansion to form 6-membered quinoline 1e. Here, the compound 1e will undergo rearrangement *via* H-shift to form the required product 3aa (Fig. 2).

**Scheme 5:**
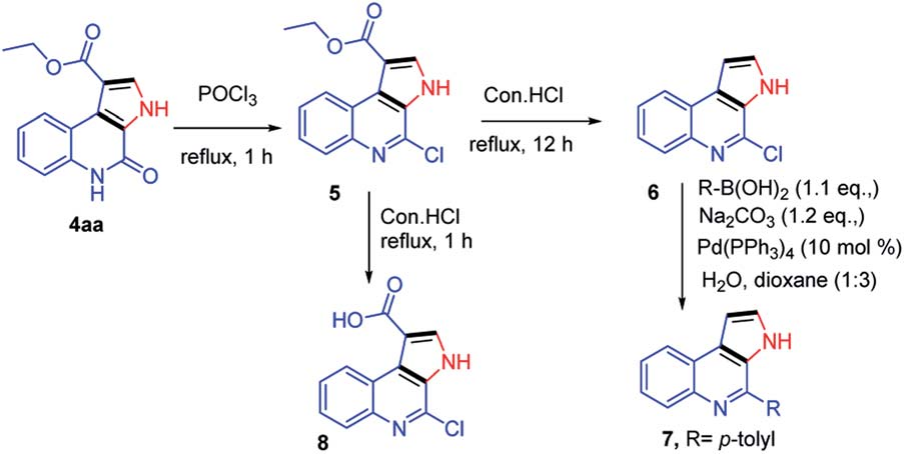
Synthetic route for marinoquinolines and its derivatives.

Next, our curiosity further extended to show the utility of the synthetic derivatives obtained from the current protocol, the synthesized compounds were demonstrated for the synthesis of natural products such as marinoquinolines. Reaction of 4aa with POCl_3_ under reflux conditions gave the corresponding product 5 in 85% yield.^12^ Next, the compound 5 was subjected to de-carboxylation in presence of con. HCl (12 h).^13^ The compound 6 further treated with *p*-tolylboronic acid under Suzuki condition furnished the desired compound 7 which are having skeletal similarities of marinoquinolines in 80% yield.^14^ When the compound 5 was refluxed in con. HCl for 1 h, gave the required acid derivative 8, which is a very useful precursor to participate in amide coupling with a variety of amines to synthesize novel amide derivatives of marinoquinolines (Scheme 5).

## Conclusions

We have developed an efficient, metal-free and greener approach for the synthesis of N-substituted and N-unsubstituted 4-oxo-4,5-dihydro-3*H*-pyrrolo[2,3-*c*]quinolines. A range of unexplored N-substituted and unsubstituted 4-oxo-4,5-dihydro-3*H*-pyrrolo[2,3-*c*]quinolines were synthesized *via* ring expansion and H-shift as the key steps in the mechanism. Further, the present methodology was also compatible with variety of substituents on both phenyl and olefinic positions on the oxindole, demonstrated the gram scale as well as marine natural product synthesis. Having prominent highlights, for example, readily available substrates, mild reaction conditions, helpful synthetic protocols, and the depicted one-pot reaction is relied upon to discover wide applications in the development of potential pharmacological candidates.

## Conflicts of interest

There are no conflicts to declare.

## Supplementary Material

RA-009-C9RA07831B-s001

RA-009-C9RA07831B-s002
