# Supplementary material for: Regioselective ring expansion followed by H-shift of 3-ylidene oxindoles: a convenient synthesis of N-substituted/un-substituted pyrrolo[2,3-c] quinolines and marinoquinolines
Source: RSC Adv. 2019 Oct 30;9(60):35068–72. doi: 10.1039/c9ra07831b (PMC9074153; doi:10.1039/c9ra07831b)
Supplement: RA-009-C9RA07831B-s001 [file RA-009-C9RA07831B-s001.pdf]

Electronic Supplementary Material (ESI) for RSC Advances.
This journal is © The Royal Society of Chemistry 2019

Supporting information for

Regioselective Ring Expansion Followed By H-shift of 3-Ylidene oxindoles: A Convenient Synthesis of *N*-substituted/un-substituted Pyrrolo[2,3-*c*] quinolines and Marinoquinolines

Gopathi Ramu,[a,b] Srinivas Ambala,[a] Jagadeesh Babu Nanubolu,[a,c] Bathini Nagendra Babu *[a,b]

[a]Department of Fluoro-Agrochemicals, CSIR-Indian Institute of Chemical Technology, Hyderabad 500 007, India
[b]Academy of Scientific and Innovative Research (AcSIR), New Delhi 110025, India
[c] Centre for X-ray Crystallography, CSIR-Indian Institute of Chemical Technology, Hyderabad, India
* Corresponding author:
Bathini Nagendra Babu, Fax: +91-40-27160512; E-mail: bathini@iict.res.in

Contents:

1. General Information **2**
2. Experimental procedures and characterization of products
   2.1. General procedure for the preparation of *N*-Substituted-3,5-dihydro-4*H*-pyrrolo [2,3-*c*]quinolin-4-ones (**3aa-3ga**): **2**
   Spectral data of *N*-Substituted-3,5-dihydro-4*H*-pyrrolo[2,3-*c*]quinolin-4-ones (**3aa-3ga**): **3-6**
   2.2. General procedure for the preparation of *N*-Unsubstituted-3,5-dihydro-4*H*-pyrrolo [2,3-*c*]quinolin-4-ones (**4aa-4qa**): **7**
   2.3. Spectral data of *N*-Unsubstituted-3,5-dihydro-4*H*-pyrrolo[2,3-*c*]quinolin-4-one (**4aa-4qa)** and synthetic route of marinoquinolines (**5-8**): **7-11**
3. ^{1}H and ^{13}C Spectra of the compounds (**3aa-3ga**) **12-27**
4. ^{1}H and ^{13}C Spectra of the compounds (**4aa-8**) **28-48**
5. X-ray Crystallographic Data of compounds **3aa** **49-50**
6. References **50**

General Information:

General Procedures.
Unless otherwise noted, all reagents were used as received from commercial sources. All air and moisture sensitive reactions were conducted under a nitrogen or argon atmosphere using flame-dried or oven-dried glassware with magnetic stirring. Tetrahydrofuran (THF) was dried over Na, benzophenone and distilled prior to use. Reactions were monitored by thin-layer chromatography carried out on silica plates (silica gel 60 F254, Merck) using UV-light, iodine and *p*-anisaldehyde for visualization. Column chromatography was carried out using silica gel (60-120 mesh or 100- 200 mesh) packed in glass columns. Technical grade EtOAc and petroleum ether used for column chromatography and were distilled prior to use. Organic solutions were concentrated under reduced pressure using a rotary evaporator. Room temperature (r.t.) is 23-25°C.

Materials. Commercial reagents were purchased from Merck, Alfa, Spectrochem or TCI, and used as received with the following exceptions. Tetrahydrofuran (THF), ethylene glycol dimethyl ether (DME), toluene and 1,4-dioxane were dried over Na with benzophenone-ketyl intermediate as indicator. Dichloroethane (DCE) and Dichloromethane DCM) were distilled over CaH_2 and acetonitrile (CH_3CN) was distilled over P_2O_5. *N,N*-Dimethylformamide (DMF) was distilled under reduced pressure. Other commercially available reagents and solvents were used without further purification.

Instrumentation. ^{1}H NMR and ^{13}C NMR spectra were recorded in $CDCl_3$ or DMSO-d_6 as solvent on Bruker AVANCE 400, INOVA instruments with 300, 400 and 500 MHz frequencies spectrometers. The coupling constant J is given in Hz. Chemical shifts (δ) were reported in ppm relative to the residual solvent signal ($CDCl_3$ δ = 7.26 for ^{1}H NMR and δ = 77.0 for ^{13}C NMR), DMSO-d_6 (^{1}H NMR: δ = 2.54and ^{13}C NMR: δ = 39.52 ppm). Chemical shifts (ppm) were recorded with tetramethylsilane (TMS) as the internal reference standard, or TMS (δ = 0.0) as internal standard and signal patterns are indicated as follows: s = singlet, d = doublet, dd = doublet of doublet, dt = doublet of triplet, t = triplet, q = quartet, qd = quartet of doublet, m = multiplet, br = broad, tt = triplet of triplet. IR spectra were recorded on a Bruker Infrared spectrophotometer and are reported as cm-1. High-resolution mass spectra (HRMS) were recorded on a Waters- spectrometer.TOF

General procedure for the preparation of *N*-Substituted-3,5-dihydro-4*H*-pyrrolo[2,3-*c*]quinolin-4-ones (3aa-3ga):

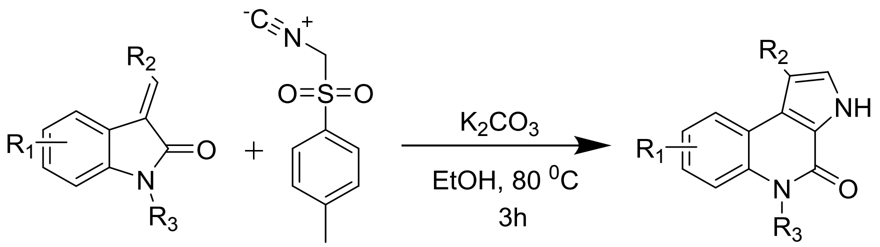

1a **2a** **3aa-3ga**

The 3–ylideneoxindole **1** (0.50 mmol), TosMIC **2a** (0.50mmol) and K_2CO_3 (1.0 mmol) (2 equiv) were stirred in 5.0 mL of EtOH was stirred at refluxing temperature for 3h. Upon the completion of the reaction (monitored by TLC), the reaction mixture was cooled to room temperature and diluted with 10 ml of water and extracted with EtOAc (3X10 ml). The organic layers were combined and washed with brine, dried over anhydrous Na_2SO_4. After the solvent was

removed under reduced pressure, the crude product was purified by flash column chromatography on silica gel (petroleum ether/EtOAc) to afford the desired products(**3aa-3ga)**.

Analytical data for the compounds 3aa-3ga:

Ethyl 5-methyl-4-oxo-4,5-dihydro-3H-pyrrolo[2,3-c]quinoline-1-carboxylate(3aa)

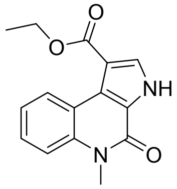

Following the general procedure (80 °C for 3 h), compound **3aa** was obtained after column chromatography (hexane:EtOAc 6:4) in 70% as a white solid. mp 283-285 °C. ^{1}H NMR (300 MHz, DMSO-d_6) δ 13.03 (s, 1H), 9.41 – 9.32 (m, 1H), 7.99 (s, 1H), 7.41 – 7.55 (m, 2H), 7.33 (t, *J* = 10.8, 4.0 Hz, 1H), 4.31 (q, *J* = 7.1 Hz, 2H), 3.74 (s, 3H), 1.35 (t, *J* = 7.1 Hz, 3H). ^{13}C NMR (101 MHz, DMSO-d_6) δ 164.48, 154.86, 137.35, 133.76, 128.21, 127.40, 124.82, 124.76, 122.32, 118.06, 115.88, 111.34, 60.34, 29.49, 14.76. HRMS calcd for $C_{15}H_{15}O_3N_2$ $[M + H]^+$: 271.1077; found: 271.1068.

Ethyl 5-benzyl-4-oxo-4,5-dihydro-3H-pyrrolo[2,3-c]quinoline-1-carboxylate(3ab)

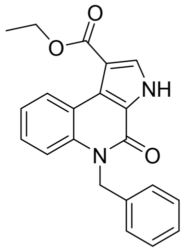

Following the general procedure (80 °C for 3 h), compound **3ab** was obtained after column chromatography (hexane:EtOAc 6:4) in 65% as a creamy white solid. mp 290-292 °C. ^{1}H NMR (300 MHz, $CDCl_3$+ DMSO-d_6) δ 12.31 (s, 1H), 9.34 (d, *J* = 7.7 Hz, 1H), 7.84 (d, *J* = 3.2 Hz, 1H), 7.19 – 7.02 (m, 8H), 5.53 (s, 2H), 4.22 (q, *J* = 7.1 Hz, 2H), 1.26 (t, *J* = 7.1 Hz, 3H). ^{13}C NMR (75 MHz, $CDCl_3$+ DMSO-d_6) δ 164.45, 155.46, 136.72, 136.16, 133.45, 128.58, 127.80, 127.39, 126.96, 126.25, 125.68, 124.22, 122.13, 118.62, 115.53, 111.59, 60.01, 45.38, 14.33. HRMS calcd for $C_{21}H_{19}O_3N_2$ $[M + H]^+$: 347.1390; found: 347.1381.

Ethyl 4-oxo-5-tosyl-4,5-dihydro-3H-pyrrolo[2,3-c]quinoline-1-carboxylate(3ac)

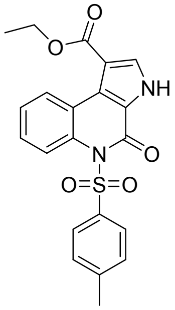

Following the general procedure (80 °C for 3 h), compound **3ac** was obtained after column chromatography (hexane:EtOAc 6:4) in 60% as a creamy white solid. mp 158-160°C.^{1}H NMR (300 MHz, DMSO-d_6) δ 11.51 (s, 1H), 8.65 (s, 1H), 7.42 – 7.43 (m, 1H), 7.37 (d, *J* = 8.1 Hz, 2H), 7.24 – 7.16 (m, 3H), 7.12 – 7.07 (m, 1H), 6.39 – 6.40 (m, 1H), 4.00 (q, *J* = 7.1 Hz, 2H), 2.34 (s, 3H), 1.06 (t, *J* = 7.1 Hz, 3H). ^{13}C NMR (75 MHz, DMSO-d_6) δ 164.46, 142.69, 137.43, 134.41, 132.08, 131.13, 129.19, 127.11, 126.30, 124.98, 124.82, 124.35, 120.15, 119.85, 113.07, 58.87, 20.94, 13.96. HRMS calcd for $C_{21}H_{19}N_2O_5S$ $[M + H]^+$: 411.1009; found: 411.1001.

Ethyl 8-methoxy-5-methyl-4-oxo-4,5-dihydro-3H-pyrrolo[2,3-c]quinoline-1-carboxylate(3ba)

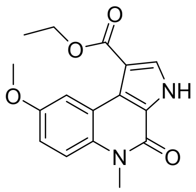

Following the general procedure (80 °C for 3 h), compound **3ba** was obtained after column chromatography (hexane:EtOAc 6:4) in 68% as a creamy white solid. mp 270-275°C. ^{1}H NMR (300 MHz, DMSO-d_6) δ 12.93 (s, 1H), 9.09 (s, 1H), 7.97 – 7.88 (m, 1H), 7.52 – 7.45 (m, 1H), 7.16 – 7.05 (m, 1H), 4.32 (q, *J* = 7.1 Hz, 2H), 3.88 (s, 3H), 3.72 (s, 3H), 1.37 (t, *J* = 7.1 Hz, 3H). ^{13}C NMR (101 MHz, DMSO-d_6) δ 164.47, 154.65, 154.47, 133.39, 131.62, 125.12, 124.77, 119.01, 116.51, 115.50, 111.27, 110.75, 60.17, 55.67, 29.43, 14.74. HRMS calcd for $C_{16}H_{17}O_4N_2$ $[M + H]^+$: 301.1183; found: 301.1172.

Ethyl 5-benzyl-8-methoxy-4-oxo-4,5-dihydro-3H-pyrrolo[2,3-c]quinoline-1-carboxylate(3bb)

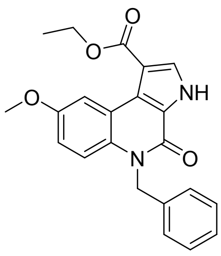

Following the general procedure (80 ^{o}C for 3 h), compound **3bb** was obtained after column chromatography (hexane:EtOAc 6:4) in 65% as a creamy white solid. mp 308-310°C. ^{1}H NMR (300 MHz, DMSO-d_6) δ 13.11 (s, 1H), 9.07 (d, *J* = 2.7 Hz, 1H), 8.05 (s, 1H), 7.38 – 7.25 (m, 6H), 7.01 (dd, *J* = 9.2, 2.7 Hz, 1H), 5.62 (s, 2H), 4.32 (q, *J* = 7.0 Hz, 2H), 3.81 (s, 3H), 1.34 (t, *J* = 7.0 Hz, 2H). ^{13}C NMR (75 MHz, DMSO-d_6) δ 164.09, 157.36, 154.19, 137.11, 133.65, 130.12, 128.62, 126.96, 126.32, 124.62, 124.31, 118.78, 117.07, 115.08, 110.90, 110.45, 59.98, 55.27, 44.47, 14.26. HRMS calcd for $C_{22} H_{21} O_4 N_2$ [M + H]$^+$: 377.1496; found: 377.1491.

Ethyl 8-methoxy-4-oxo-5-tosyl-4,5-dihydro-3H-pyrrolo[2,3-c]quinoline-1-carboxylate(3bc)

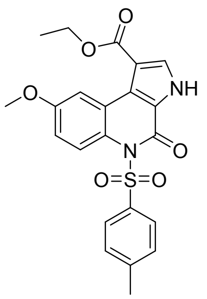

Following the general procedure (80 ^{o}C for 3 h), compound **3bc** was obtained after column chromatography (hexane:EtOAc 6:4) in 58% as a creamy white solid. mp 160-162°C. ^{1}H NMR (300 MHz, DMSO-d_6) δ 11.45 (s, 1H), 8.60 (s, 1H), 7.37 (s, 1H), 7.23 (d, *J* = 8.1 Hz, 1H), 7.13 (d, *J* = 8.1 Hz, 1H), 7.07 (d, *J* = 8.8 Hz, 1H), 6.80 (d, *J* = 8.7, 2.8 Hz, 1H), 6.63 (d, *J* = 2.8 Hz, 1H), 6.39 – 6.35 (m, 1H), 4.03 (q, *J* = 7.0 Hz, 2H), 3.69 (s, 3H), 2.34 (s, 3H), 1.12 (t, *J* = 7.0 Hz, 3H). ^{13}C NMR (101 MHz, DMSO-d_6) δ 165.07, 157.33, 142.88, 137.88, 134.57, 129.51, 128.64, 127.23, 126.69, 125.19, 120.96, 120.45, 117.61, 113.23, 112.96, 59.43, 55.59, 21.45, 14.54. HRMS calcd for $C_{22} H_{21} O_6 N_2 S$ [M + H]$^+$: 441.1115; found: 441.1128.

Ethyl 8-fluoro-5-methyl-4-oxo-4,5-dihydro-3H-pyrrolo[2,3-c]quinoline-1-carboxylate(3ca)

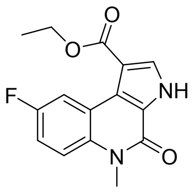

Following the general procedure (80 ^{o}C for 3 h), compound **3ca** was obtained after column chromatography (hexane:EtOAc 6:4) in 66% as a creamy white solid. mp 333-335°C. ^{1}H NMR (300 MHz, DMSO-d_6) δ 13.12 (s, 1H), 9.23 (s, 1H), 8.01 (s, 1H), 7.75 – 7.61 (m, 1H), 7.39 – 7.31 (m, 1H), 4.32 (q, *J* = 7.0 Hz, 2H), 3.73 (s, 3H), 1.35 (t, *J* = 7.0 Hz, 3H). ^{13}C NMR (101 MHz, DMSO-d_6) δ 164.50, 158.83, 156.48, 154.56, 134.14, 133.92, 125.27, 124.04, 119.21, 117.80, 117.72, 115.43, 115.20, 112.89, 112.63, 111.34, 60.53, 29.81, 14.73. HRMS calcd for $C_{15} H_{14} O_3 N_2 F$ [M + H]$^+$: 289.0983; found: 289.0976.

Ethyl 5-benzyl-8-fluoro-4-oxo-4,5-dihydro-3H-pyrrolo[2,3-c]quinoline-1-carboxylate(3cb)

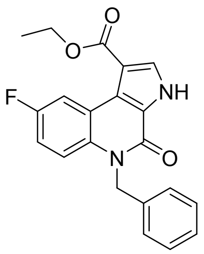

Following the general procedure (80 ^{o}C for 3 h), compound **3cb** was obtained after column chromatography (hexane:EtOAc 6:4) in 63% as a creamy white solid. mp 340-341°C. ^{1}H NMR (400 MHz, DMSO-d_6) δ 13.24 (s, 1H), 9.26 (dd, *J* = 11.2, 3.0 Hz, 1H), 8.08 (s, 1H), 7.43 – 7.39 (m, 1H), 7.34 – 7.17 (m, 6H), 5.66 (s, 2H), 4.33 (q, *J* = 7.1 Hz, 2H), 1.36 (t, *J* = 7.1 Hz, 3H). ^{13}C NMR (75 MHz, DMSO-d_6) δ 163.97, 158.67, 155.53, 154.42, 136.81, 133.83, 132.64, 128.67, 127.03, 126.32, 124.46, 123.99, 119.15, 119.01, 117.81, 117.70, 115.01, 114.70, 112.65, 112.31, 110.98, 60.07, 44.69, 14.22. HRMS calcd for $C_{21} H_{18} O_3 N_2 F$ [M + H]$^+$: 365.1296; found: 365.1289

Ethyl 8-fluoro-4-oxo-5-tosyl-4,5-dihydro-3H-pyrrolo[2,3-c]quinoline-1-carboxylate(3cc)

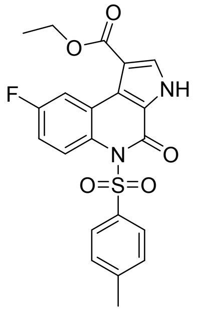

Following the general procedure (80 ^{o}C for 3 h), compound **3cc** was obtained after column chromatography (hexane:EtOAc 6:4) in 55% as a creamy white solid. mp 158-160°C. ^{1}H NMR (300 MHz, DMSO-d$_6$) δ 11.52 (s, 1H), 8.86 (s, 1H), 7.39 – 7.30 (m, 1H), 7.30 (d, *J* = 8.2 Hz, 1H), 7.16 – 7.09 (m, 2H), 7.11 – 7.04 (m, 1H), 6.93 (dd, *J* = 9.7, 2.9 Hz, 1H), 6.50 (s, 1H), 4.01 (q, *J* = 7.1 Hz, 2H), 2.34 (s, 3H), 1.08 (t, *J* = 7.1 Hz, 3H). ^{13}C NMR (75 MHz, DMSO-d$_6$) δ 164.07, 160.92, 157.71, 142.52, 137.25, 134.63, 134.51, 130.29, 129.07, 127.94, 127.82, 126.15, 124.81, 120.01, 119.18, 118.56, 118.26, 113.73, 113.44, 112.87, 58.76, 20.88, 13.88. HRMS calcd for C_{21} H_{18} O_5 N_2 F S [M + H]$^+$: 429.0915; found: 429.0896.

Ethyl 8-chloro-5-methyl-4-oxo-4,5-dihydro-3H-pyrrolo[2,3-c]quinoline-1-carboxylate(3da)

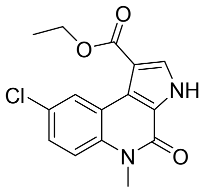

Following the general procedure (80 ^{o}C for 3 h), compound **3da** was obtained after column chromatography (hexane:EtOAc 6:4) in 67% as a creamy white solid. mp 298-300°C**.** ^{1}H NMR (300 MHz, $CDCl_3$+ DMSO-d$_6$) δ 13.10 (s, 1H), 9.43 (d, *J* = 2.1 Hz, 1H), 7.98 (d, *J* = 3.1 Hz, 1H), 7.59 – 7.48 (m, 2H), 4.31 (q, *J* = 7.1 Hz, 2H), 3.69 (s, 3H), 1.35 (t, *J* = 7.1 Hz, 3H). ^{13}C NMR (101 MHz, DMSO-d$_6$) δ 164.46, 154.62, 136.13, 134.08, 127.71, 126.70, 126.42, 125.17, 123.65, 119.33, 117.83, 111.35, 60.58, 29.71, 14.72. HRMS calcd for C_{15} H_{14} O_3 N_2 Cl [M + H]$^+$: 305.0687; found: 305.0678.

Ethyl 5-benzyl-8-chloro-4-oxo-4,5-dihydro-3H-pyrrolo[2,3-c]quinoline-1-carboxylate(3db)

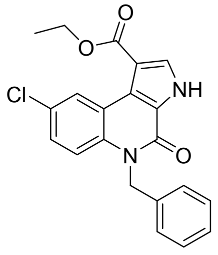

Following the general procedure (80 ^{o}C for 3 h), compound **3db** was obtained after column chromatography (hexane:EtOAc 6:4) in 62% as a creamy white solid. mp 286-289°C**.** ^{1}H NMR (300 MHz, DMSO-d$_6$) δ 13.28 (s, 1H), 9.51 (s, 1H), 8.09 (d, *J* = 3.0 Hz, 1H), 7.44 – 7.42 (m, 2H), 7.35 – 7.16 (m, 5H), 5.65 (s, 2H), 4.34 (q, *J* = 7.0 Hz, 2H), 1.37 (t, *J* = 7.0 Hz, 3H). ^{13}C NMR (101 MHz, DMSO-d$_6$) δ 164.44, 154.98, 137.17, 135.22, 134.50, 129.19, 127.72, 127.56, 126.85, 126.79, 126.70, 124.93, 124.15, 119.77, 118.31, 111.52, 60.62, 45.12, 14.73**.** HRMS calcd for C_{21} H_{18} O_3 N_2 Cl [M + H]$^+$: 381.1000; found: 381.1003.

Ethyl 8-chloro-4-oxo-5-tosyl-4,5-dihydro-3H-pyrrolo[2,3-c]quinoline-1-carboxylate(3dc)

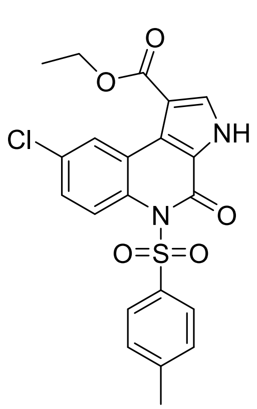

Following the general procedure (80 ^{o}C for 3h), compound **3dc** was obtained after column chromatography (hexane:EtOAc 6:4) in 58% as a creamy white solid. mp 158-160°C**.** ^{1}H NMR (300 MHz, $CDCl_3$+ DMSO-d$_6$) δ 11.54 (s, 1H), 8.87 (s, 1H), 7.44 – 7.32 (m, 2H), 7.27 (d, *J* = 7.6 Hz, 1H), 7.30 – 7.18 (m, 2H), 7.14 (d, *J* = 10.3 Hz, 1H), 6.50 (s, 1H), 3.98 (q, *J* = 6.9 Hz, 2H), 2.34 (s, 3H), 1.05 (t, *J* = 6.9 Hz, 3H). ^{13}C NMR (101 MHz, DMSO-d$_6$) δ 164.59, 143.38, 137.68, 133.92, 133.85, 132.09, 129.79, 129.65, 127.38, 126.92, 126.80, 125.51, 120.60, 119.25, 113.63, 59.37, 21.46, 14.41. HRMS calcd for $C_{21}H_{18}$ Cl N_2 O_5 S [M + H]$^+$: 445.0619; found :445.1590.

Ethyl 5-benzyl-8-bromo-4-oxo-4,5-dihydro-3H-pyrrolo[2,3-c]quinoline-1-carboxylate(3ea)

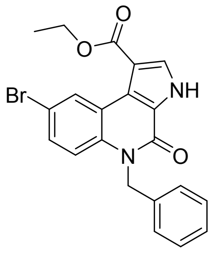

Following the general procedure (80 °C for 3 h), compound **3ea** was obtained after column chromatography (hexane:EtOAc 6:4) in 61% as a creamy white solid. mp 298-300°C. ^{1}H NMR (300 MHz, DMSO-d$_6$) δ 13.28 (s, 1H), 9.65 (d, *J* = 2.0 Hz, 1H), 8.08 (s, 1H), 7.55 (dd, *J* = 9.0, 1.9 Hz, 1H), 7.40 – 7.33 (m, 3H), 7.21 – 7.12 (m, 3H), 5.64 (s, 2H), 4.34 (q, *J* = 7.0 Hz, 2H), 1.37 (t, *J* = 7.0 Hz, 3H). ^{13}C NMR (101 MHz, DMSO-d$_6$) δ 164.46, 154.98, 137.14, 135.59, 134.54, 130.50, 129.67, 129.19, 127.56, 126.79, 124.90, 124.06, 120.22, 118.66, 114.90, 111.52, 60.63, 45.08, 14.75. HRMS calcd for $C_{21}H_{18}O_3N_2Br$ [M + H]$^+$: 425.0495; found: 425.0491.

1-benzoyl-5-methyl-3,5-dihydro-4H-pyrrolo[2,3-c]quinolin-4-one(3fa)

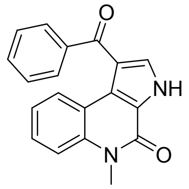

Following the general procedure (80 °C for 3 h), compound **3fa** was obtained after column chromatography (hexane:EtOAc 6:4) in 58% as a pale yellow solid. mp 232-234°C. ^{1}H NMR (500 MHz, CDCl$_3$) δ 12.21 (s, 1H), 9.13 (d, *J* = 8.1 Hz, 1H), 7.92 (dd, *J* = 5.1, 3.3 Hz, 2H), 7.68 (d, *J* = 2.6 Hz, 1H), 7.63 – 7.59 (m, 1H), 7.56 – 7.50 (m, 4H), 7.36 (ddd, *J* = 8.2, 6.8, 1.4 Hz, 1H), 3.90 (s, 3H). ^{13}C NMR(126 MHz, CDCl$_3$) δ 191.80, 155.84, 140.37, 136.90, 135.22, 132.18, 129.69, 128.42, 128.16, 127.72, 126.54, 124.81, 122.93, 120.58, 118.65, 115.06, 29.75. HRMS calcd for $C_{19}H_{15}O_2N_2$ [M + H]$^+$: 303.1128; found: 303.1138.

1-benzoyl-5-benzyl-3,5-dihydro-4H-pyrrolo[2,3-c]quinolin-4-one(3fb)

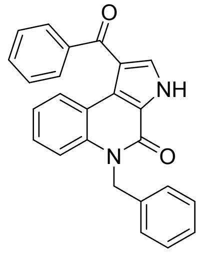

Following the general procedure (80 °C for 3 h), compound **3fb** was obtained after column chromatography (hexane:EtOAc 8:2) in 53% as a pale yellow solid. mp 212-214°C. ^{1}H NMR (300 MHz, CDCl$_3$) δ 11.93 (s, 1H), 9.10 (d, *J* = 7.6 Hz, 1H), 7.90 (d, *J* = 7.4 Hz, 2H), 7.62 (t, *J* = 7.3 Hz, 1H), 7.56 – 7.47 (m, 3H), 7.45 – 7.36 (m, 2H), 7.31 (dd, *J* = 9.7, 4.6 Hz, 1H), 7.23 – 7.11 (m, 5H), 5.76 (s, 2H). ^{13}C NMR (75 MHz, CDCl$_3$) δ 196.22, 160.22, 145.24, 141.82, 141.23, 140.45, 136.86, 134.19, 133.57, 133.22, 132.57, 132.15, 131.96, 131.29, 130.69, 129.69, 126.85, 124.75, 123.41, 120.67, 50.07. HRMS calcd for $C_{25}H_{19}O_2N_2$ [M + H]$^+$: 379.1441; found: 379.1467.

5-methyl-1-phenyl-3,5-dihydro-4H-pyrrolo[2,3-c]quinolin-4-one(3ga)

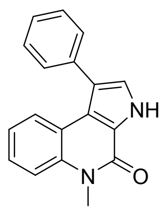

Following the general procedure (80 °C for 3 h), compound **3ga** was obtained after column chromatography (hexane:EtOAc 8:2) in 58% as a pale yellow solid. mp 222-224°C. ^{1}H NMR (500 MHz, CDCl$_3$) δ 11.24 (s, 1H), 7.89 (dd, *J* = 8.0, 1.1 Hz, 1H), 7.57 – 7.53 (m, 2H), 7.50 – 7.44 (m, 3H), 7.44 – 7.38 (m, 2H), 7.29 (d, *J* = 2.6 Hz, 1H), 7.11 – 7.05 (m, 1H), 3.90 (s, 3H). ^{13}C NMR(101 MHz, CDCl$_3$) δ 156.00, 136.66, 135.73, 130.01, 128.54, 127.20, 126.66, 125.67, 124.04, 123.11, 122.03, 121.69, 119.44, 115.25, 29.42. HRMS calcd for $C_{18}H_{15}ON_2$ [M + H]$^+$: = 275.1178; found: 275.1185.

General procedure for the preparation of *N*-Substituted-3,5-dihydro-4*H*-pyrrolo[2,3-*c*]quinolin-4-ones (4aa-4qa):

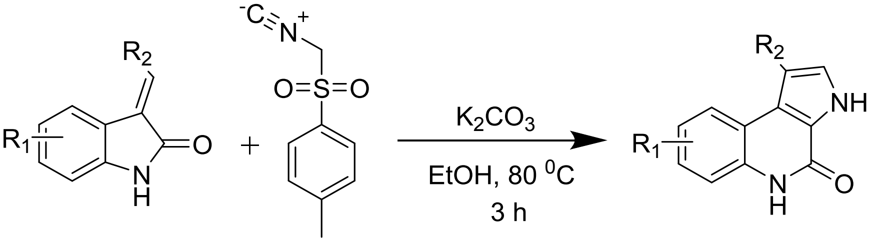

1a **2a** **4aa-4qa**

The 3–ylideneoxindole **1** (0.50 mmol), TosMIC **2a** (0.50 mmol) and K_2CO_3 (1.00 mmol) (2 equiv) were stirred in 5.0 mL of EtOH was stirred at refluxing temperature for 3h. Upon the completion of the reaction (monitored by TLC), the reaction mixture was cooled to room temperature and diluted with 10 ml of water and extracted with EtOAc (3X10 ml). The organic layers were combined and washed with brine, dried over anhydrous Na_2SO_4. After the solvent was removed under reduced pressure, the crude product was purified by flash column chromatography on silica gel (petroleum ether/EtOAc) to afford the desired products (**4aa-4qa)**.

Analytical data for the compounds 4aa-4qa:

Ethyl 4-oxo-4,5-dihydro-3H-pyrrolo[2,3-c]quinoline-1-carboxylate(4aa)

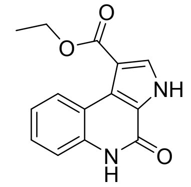

Following the general procedure (80 ^oC for 3 h), compound **4aa** was obtained after column chromatography (hexane: EtOAc 6:4) in 88% as a white solid. mp 290-292 ^oC. ^1H NMR (400 MHz, DMSO-d$_6$) δ 12.96 (s, 1H), 11.67 (s, 1H), 9.22 (d, *J* = 8.0 Hz, 1H), 7.99 (s, 1H), 7.45 – 7.37 (m, 2H), 7.22 (dd, *J* = 8.2, 6.2, 2.2 Hz, 1H), 4.31 (q, *J* = 7.1 Hz, 2H), 1.34 (t, *J* = 7.1 Hz, 3H). ^{13}C NMR (75 MHz, $CDCl_3$+ DMSO-d$_6$) δ 169.22, 159.91, 141.24, 138.20, 132.49, 131.65, 130.76, 129.98, 126.75, 121.91, 121.17, 116.28, 65.04, 19.51. HRMS calcd for $C_{14}H_{13}O_3N_2$ $[M + H]^+$: 257.0921; found: 257.0913.

Ethyl 8-methyl-4-oxo-4,5-dihydro-3H-pyrrolo[2,3-c]quinoline-1-carboxylate(4ba)

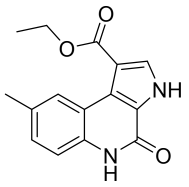

Following the general procedure (80 ^oC for 3 h), compound **4ba** was obtained after column chromatography (hexane:EtOAc 6:4) in 78% as a white solid. mp 276-278^oC**.** ^1H NMR (300 MHz, DMSO-d$_6$) δ 12.99 (s, 1H), 11.64 (s, 1H), 9.07 (s, 1H), 7.99 (d, *J* = 3.2 Hz, 1H), 7.33 (d, *J* = 8.3 Hz, 1H), 7.23 (d, *J* = 7.7 Hz, 1H), 4.32 (q, *J* = 7.1 Hz, 2H), 2.40 (s, 3H), 1.36 (t, *J* = 7.1 Hz, 3H). ^{13}C NMR (75 MHz, DMSO-d$_6$) δ 163.95, 154.54, 133.91, 132.87, 130.23, 128.27, 126.18, 125.31, 124.83, 116.58, 115.77, 110.98, 59.79, 20.95, 14.27. HRMS calcd for $C_{15}H_{15}O_3N_2$ $[M + H]^+$: 271.1077; found: 271.1068.

Ethyl 8-methoxy-4-oxo-4,5-dihydro-3H-pyrrolo[2,3-c]quinoline-1-carboxylate(4ca)

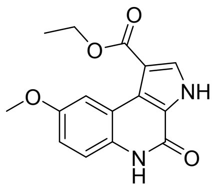

Following the general procedure (80 ^oC for 3 h), compound **4ca** was obtained after column chromatography (hexane:EtOAc 6:4) in 82% as a creamy white solid. mp 280-282^oC**.** ^1H NMR (300 MHz, $CDCl_3$+ DMSO-d$_6$) δ 12.82 (s, 1H), 11.45 (s, 1H), 8.91 (d, *J* = 2.4 Hz, 1H), 7.88 (d, *J* = 2.8 Hz, 1H), 7.30 (d, *J* = 8.9 Hz, 1H), 6.94 – 6.80 (m, 1H), 4.29 (q, *J* = 7.0 Hz, 2H), 3.82 (s, 3H), 1.34 (t, *J* = 7.0 Hz, 3H). ^{13}C NMR (75 MHz, DMSO-d$_6$) δ 163.93, 154.36, 154.02,

132.55, 130.10, 125.63, 125.00, 117.48, 116.79, 115.28, 110.96, 109.21, 59.57, 55.10, 14.21. HRMS calcd for $C_{15}H_{15}O_4N_2$ $[M + H]^+$: 287.1026; found: 287.1015.

Ethyl 8-fluoro-4-oxo-4,5-dihydro-3H-pyrrolo[2,3-c]quinoline-1-carboxylate(4da)

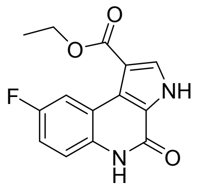

Following the general procedure (80 °C for 3 h), compound **4da** was obtained after column chromatography (hexane:EtOAc 6:4) in 80% as a creamy white solid. mp 326-329°C. ^1H NMR (300 MHz, DMSO-d_6) δ 13.08 (s, 1H), 11.76 (s, 1H), 9.07 (dd, J = 11.5, 2.6 Hz, 1H), 8.00 (d, J = 3.1 Hz, 1H), 7.42 – 7.32 (m, 1H), 7.30 – 7.28 (m, 1H), 4.30 (q, J = 7.0 Hz, 2H), 1.33 (t, J = 7.1 Hz, 3H). ^{13}C NMR (101 MHz, DMSO-d_6) δ 164.47, 158.62, 156.28, 154.87, 133.60, 133.17, 125.67, 125.35, 118.09, 117.98, 117.89, 115.52, 115.28, 112.30, 112.04, 111.53, 60.47, 14.72. HRMS calcd for $C_{14}H_{12}O_3N_2F$ $[M + H]^+$: 275.0826; found: 275.0818.

Ethyl 8-chloro-4-oxo-4,5-dihydro-3H-pyrrolo[2,3-c]quinoline-1-carboxylate(4ea)

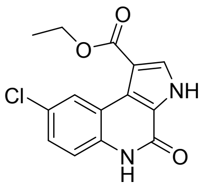

3d

Following the general procedure (80 °C for 3 h), compound **4ea** was obtained after column chromatography (hexane:EtOAc 6:4) in 78% as a creamy white solid. mp 300-302°C. ^1H NMR (300 MHz, DMSO-d_6) δ 13.15 (s, 1H), 11.86 (s, 1H), 9.36 (s, 1H), 8.03 (d, J = 2.9 Hz, 1H), 7.50 – 7.40 (m, 2H), 4.33 (q, J = 7.0 Hz, 2H), 1.36 (t, J = 7.0 Hz, 3H). ^{13}C NMR(75 MHz, DMSO-d_6) δ 163.92, 154.44, 134.77, 133.21, 127.04, 125.56, 125.14, 124.39, 117.91, 117.61, 111.07, 59.98, 14.22. HRMS calcd for $C_{14}H_{12}O_3N_2Cl$ $[M + H]^+$: 291.0531; found: 291.0524.

Ethyl 8-bromo-4-oxo-4,5-dihydro-3H-pyrrolo[2,3-c]quinoline-1-carboxylate(4fa)

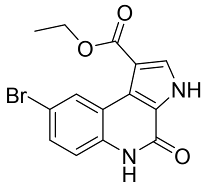

Following the general procedure (80 °C for 3 h), compound **4fa** was obtained after column chromatography (hexane:EtOAc 6:4) in 75% as a creamy white solid. mp 302-304°C**.** ^1H NMR (300 MHz, DMSO-d_6) δ 13.15 (s, 1H), 11.85 (s, 1H), 9.50 (d, J = 1.7 Hz, 1H), 8.01 (s, 1H), 7.65 – 7.57 (m, 1H), 7.39 (d, J = 9.8 Hz, 1H), 4.33 (q, J = 7.0 Hz, 2H), 1.36 (t, J = 7.0 Hz, 3H). ^{13}C NMR (101 MHz, DMSO-d_6) δ 164.47, 154.96, 135.61, 133.80, 130.30, 129.02, 125.61, 124.79, 118.93, 118.48, 114.05, 111.58, 60.54, 14.76. HRMS calcd for $C_{14}H_{12}O_3N_2Br$ $[M + H]^+$: 335.0026; found: 335.0019.

Ethyl 8-nitro-4-oxo-4,5-dihydro-3H-pyrrolo[2,3-c]quinoline-1-carboxylate(4ga)

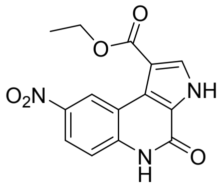

Following the general procedure (80°C for 3 h), compound **4ga**was obtained after column chromatography (hexane:EtOAc 6:4) in 70% as a pale yellow solid. mp 360-362°C**.** ^1H NMR (300 MHz, DMSO-d_6) δ 13.28 (s, 1H), 12.26 (s, 1H), 10.28 (s, 1H), 8.26 (d, J = 9.0 Hz, 1H), 8.07 (s, 1H), 7.54 (d, J = 9.1 Hz, 1H), 4.36 (q, J = 7.1 Hz, 2H)), 1.36 (t, J = 7.1 Hz, 3H). ^{13}C NMR (75 MHz, $CDCl_3$+ DMSO-d_6) δ 168.99, 159.86, 146.64, 145.96, 138.85, 130.29, 129.85, 128.14, 127.38, 121.86, 121.50, 116.55, 65.37, 19.45. HRMS calcd for $C_{14}H_{12}O_5N_3$ $[M + H]^+$: 302.0771; found302.0791.

Methyl 4-oxo-4,5-dihydro-3H-pyrrolo[2,3-c]quinoline-1-carboxylate(4ha)

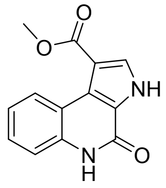

Following the general procedure (80°C for 3 h), compound **4ha** was obtained after column chromatography (hexane:EtOAc 6:4) in 80% as a white solid. mp 348-350°C**.** ^1H NMR (300 MHz, DMSO-d_6) δ 13.03 (s, 1H), 11.71 (s, 1H), 9.24 (d, J = 8.1 Hz, 1H), 8.01 (s, 1H), 7.45 – 7.37 (m, 2H), 7.30 – 7.23 (m, 1H), 3.84 (s, 3H). ^{13}C NMR (101 MHz, DMSO-d_6) δ 164.92, 155.15, 136.53, 133.46,

127.78, 126.87, 126.05, 125.26, 122.03, 117.14, 116.44, 111.18, 51.84. HRMS calcd for C_{13} H_{11} O_3 N_2 $[M + H]^+$: 243.0764; found: 243.0756.

1-phenyl-3,5-dihydro-4H-pyrrolo[2,3-c]quinolin-4-one(4ia)

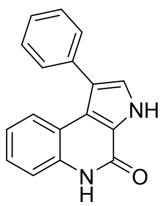

Following the general procedure (80 °C for 3 h), compound **4ia** was obtained after column chromatography (hexane:EtOAc 6:4) in 60% as a creamy white solid. mp 310-312°C. ^{1}H NMR (300 MHz, $CDCl_3$+ DMSO-d_6) δ 11.42 (s, 1H), 10.48 (s, 1H), 6.70 (d, J = 8.0 Hz, 1H), 6.52 – 6.40 (m, 4H), 6.44 (d, J = 7.6 Hz, 2H), 6.30 – 6.24 (m, 2H), 5.98 (t, J = 7.5 Hz, 1H). ^{13}C NMR (75 MHz, DMSO-d_6) δ 155.10, 135.54, 135.48, 129.53, 128.34, 126.84, 125.93, 125.46, 123.14, 122.85, 122.31, 121.01, 120.63, 117.56, 116.14. HRMS calcd for C_{17} H_{13} O N_2 $[M + H]^+$: 261.1022; found: 261.1012.

1-(4-methoxyphenyl)-3,5-dihydro-4H-pyrrolo[2,3-c]quinolin-4-one(4ja)

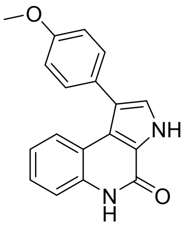

Following the general procedure (80 °C for 3 h), compound **4ja** was obtained after column chromatography (hexane:EtOAc 6:4) in 58% as a creamy white solid. mp 320-322°C. ^{1}H NMR (400 MHz, DMSO-d_6) δ 12.34 (s, 1H), 11.43 (s, 1H), 7.67 (d, J = 7.8 Hz, 1H), 7.41 – 7.30 (m, 3H), 7.25 (d, J = 7.3 Hz, 2H), 7.02 (d, J = 8.1 Hz, 2H), 6.95 (t, J = 7.3 Hz, 1H), 3.84 (s, 3H). ^{13}C NMR (75 MHz, DMSO-d_6) δ 158.36, 155.03, 135.52, 130.79, 127.62, 125.98, 125.44, 122.98, 122.89, 122.23, 121.09, 120.26, 117.65, 116.08, 113.90, 55.10. HRMS calcd for C_{18} H_{15} O_2 N_2 $[M + H]^+$: 291.1128; found: 291.1118.

1-(4-chlorophenyl)-3,5-dihydro-4H-pyrrolo[2,3-c]quinolin-4-one(4ka)

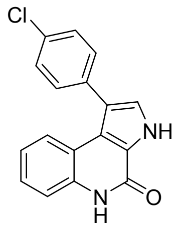

Following the general procedure (80 °C for 3 h), compound **4ka** was obtained after column chromatography (hexane:EtOAc 6:4) in 55% as a creamy white solid. mp 328-330°C. ^{1}H NMR (300 MHz, $CDCl_3$+ DMSO-d_6) δ 12.50 (s, 1H), 11.49 (s, 1H), 7.60 (d, J = 7.9 Hz, 1H), 7.53 – 7.48 (m, 4H), 7.39 – 7.26 (m, 2H), 7.28 (t, J = 7.5 Hz, 1H), 6.99 (t, J = 7.5 Hz, 1H). ^{13}C NMR (101 MHz, DMSO-d_6) δ 155.49, 136.08, 134.98, 132.20, 131.90, 128.99, 126.73, 126.41, 123.82, 123.21, 122.70, 121.78, 119.77, 117.83, 116.73. HRMS calcd for C_{17} H_{12} O N_2 Cl $[M + H]^+$: 295.0633; found: 295.0625.

1-(4-nitrophenyl)-3,5-dihydro-4H-pyrrolo[2,3-c]quinolin-4-one(4la)

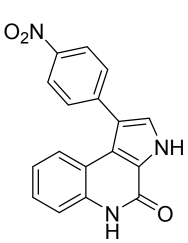

Following the general procedure (80 °C for 3 h), compound **4la** was obtained after column chromatography (hexane:EtOAc 6:4) in 45% as a pale yellow solid. mp 370-373°C. ^{1}H NMR (400 MHz, DMSO-d_6) δ 12.66 (s, 1H), 11.55 (s, 1H), 8.34 (d, J = 8.8 Hz, 2H), 7.85 – 7.80 (m, 2H), 7.66 (d, J = 8.0 Hz, 1H), 7.54 (d, J = 2.9 Hz, 1H), 7.43 (d, J = 8.2 Hz, 1H), 7.35 – 7.28 (m, 1H), 7.07 – 7.00 (m, 1H). ^{13}C NMR (101 MHz, DMSO-d_6) δ 155.42, 146.66, 143.43, 136.17, 130.91, 127.35, 127.06, 124.39, 124.27, 123.14, 122.86, 121.98, 119.26, 117.47, 116.88. HRMS calcd for C_{17} H_{12} O_3 N_3 $[M + H]^+$: 306.0873; found: 306.0869.

1-(4-methoxybenzoyl)-3,5-dihydro-4H-pyrrolo[2,3-c]quinolin-4-one(4ma)

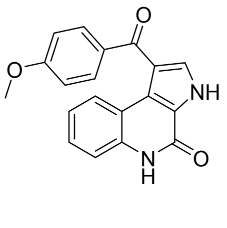

Following the general procedure (80 °C for 3 h), compound **4ma** was obtained after column chromatography (hexane:EtOAc 6:4) in 57% as a creamy white solid. mp 245-248°C. ^{1}H

NMR (300 MHz, DMSO-d$_6$) δ 13.12 (s, 1H), 11.80 (s, 1H), 8.88 (d, J = 8.0 Hz, 1H), 7.71 (d, J = 3.2 Hz, 1H), 7.52 – 7.35 (m, 5H), 7.27 – 7.18 (m, 2H), 3.85 (s, 3H). ^{13}C NMR (75 MHz, DMSO-d$_6$) δ 190.56, 159.09, 154.81, 141.37, 136.10, 135.08, 129.51, 127.37, 126.11, 125.98, 125.12, 121.71, 121.41, 119.45, 117.91, 116.80, 115.99, 113.85, 55.25. HRMS calcd for $C_{19}H_{15}O_3N_2$ [M + H]$^+$: 319.1077; found: 319.1064.

1-(3-chlorobenzoyl)-3,5-dihydro-4H-pyrrolo[2,3-c]quinolin-4-one(4na)

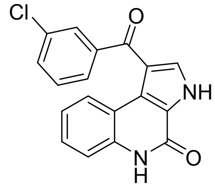

Following the general procedure (80 °C for 3 h), compound **4na** was obtained after column chromatography (hexane:EtOAc 6:4) in 56% as a creamy white solid. mp 275-278°C. ^{1}H NMR (300 MHz, DMSO-d$_6$) δ 13.11 (s, 1H), 11.78 (s, 1H), 8.86 (d, J = 8.1 Hz, 1H), 7.85 (d, J = 7.1 Hz, 2H), 7.67 – 7.60 (m, 2H), 7.58 (d, J = 7.6 Hz, 1H), 7.43 – 7.38 (m, 2H), 7.25 – 7.16 (m, 1H). ^{13}C NMR (101 MHz, DMSO-d$_6$) δ 189.84, 155.27, 142.55, 136.66, 136.24, 133.77, 132.14, 130.86, 129.08, 128.35, 127.98, 126.67, 126.54, 125.84, 121.95, 119.63, 117.22, 116.50. HRMS calcd for $C_{18}H_{12}O_2N_2Cl$ [M + H]$^+$: 323.0582; found: 323.0574.

1-(3-methoxy-4-nitrobenzoyl)-3,5-dihydro-4H-pyrrolo[2,3-c]quinolin-4-one(4oa)

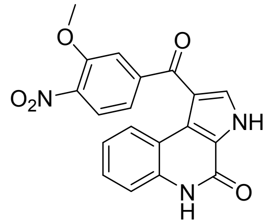

Following the general procedure (80 °C for 3 h), compound **4oa** was obtained after column chromatography (hexane:EtOAc 6:4) in 42% as a pale yellow solid. mp 260-262°C**.** ^{1}H NMR (300 MHz, DMSO-d$_6$) δ 13.15 (s, 1H), 11.76 (s, 1H), 8.76 (d, J = 8.1 Hz, 1H), 8.33 (d, J = 2.1 Hz, 1H), 8.16 (dd, J = 8.8, 2.0 Hz, 1H), 7.84 (s, 1H), 7.53 (d, J = 8.9 Hz, 1H), 7.46 – 7.37 (m, 2H), 7.19 (t, J = 7.2 Hz, 1H), 4.04 (s, 3H). ^{13}C NMR (126 MHz, DMSO-d$_6$) δ 187.29, 155.86, 146.78, 144.44, 142.00, 139.41, 136.10, 132.53, 131.11, 127.11, 126.21, 123.41, 117.24, 116.40, 115.02, 112.09, 57.85. HRMS calcd for $C_{19}H_{14}O_5N_3$ [M + H]$^+$: 364.0928; found: 364.0924.

1-(2-naphthoyl)-8-chloro-3,5-dihydro-4H-pyrrolo[2,3-c]quinolin-4-one(4pa)

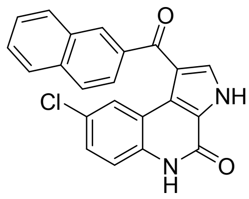

Following the general procedure (80 °C for 3 h), compound **4pa** was obtained after column chromatography (hexane:EtOAc 6:4) in 60% as a creamy white solid. mp 260-263°C**.** ^{1}H NMR (300 MHz, DMSO-d$_6$) δ 13.26 (s, 1H), 11.93 (s, 1H), 9.02 (s, 1H), 8.47 (s, 1H), 8.17 – 8.03 (m, 3H), 7.96 (dd, J = 8.5, 1.5 Hz, 1H), 7.85 (d, J = 3.2 Hz, 1H), 7.74 – 7.66 (m, 2H), 7.48 – 7.30 (m, 2H). ^{13}C NMR (75 MHz, DMSO-d$_6$) δ 191.01, 154.61, 137.07, 135.76, 134.91, 134.60, 131.97, 130.70, 129.43, 128.18, 127.62, 127.21, 126.82, 125.61, 125.46, 125.34, 124.99, 119.60, 118.13, 117.72. HRMS calcd for $C_{22}H_{14}O_2N_2Cl$ [M + H]$^+$: 373.0738; found: 373.0734.

1-(2-naphthoyl)-8-bromo-3,5-dihydro-4H-pyrrolo[2,3-c]quinolin-4-one(4qa)

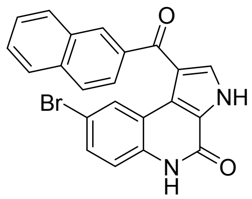

Following the general procedure (80 °C for 3 h), compound **4qa** was obtained after column chromatography (hexane:EtOAc 6:4) in 56% as a creamy white solid. mp 290-292°C**.** ^{1}H NMR (300 MHz, DMSO-d$_6$) δ 13.27 (s, 1H), 11.94 (s, 1H), 9.17 (d, J = 2.1 Hz, 1H), 8.47 (s, 1H), 8.17 – 8.02 (m, 3H), 7.99 – 7.93 (m, 1H), 7.85 (d, J = 3.2 Hz, 1H), 7.64 – 7.50 (m, 3H), 7.42 (d, J = 8.8 Hz, 1H). ^{13}C NMR (75 MHz, DMSO-d$_6$) δ 190.99, 154.62, 137.09, 135.73, 134.91, 134.60, 131.98, 130.66, 129.41, 128.16, 127.61, 127.19, 126.81, 125.63, 125.47, 125.33, 125.00, 119.61, 118.14, 117.71. HRMS calcd for $C_{22}H_{14}O_2N_2Br$ [M + H]$^+$: 417.0233; found: 417.0226.

Ethyl 4-chloro-3H-pyrrolo[2,3-c]quinoline-1-carboxylate(5)

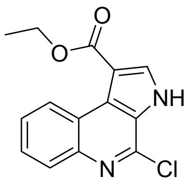

Following the general procedure (**Scheme 5**), compound **5** was obtained after column chromatography (hexane:EtOAc 7:3) in 85% as a creamy white solid. mp 270-272°C. ^{1}H NMR (500 MHz, $CDCl_3$) δ 9.64 (s, 1H), 9.38 (s, 1H), 8.15 (dd, J = 34.0, 10.8 Hz, 2H), 7.77 – 7.57 (m, 2H), 4.48 (q, J = 7.1 Hz, 2H), 1.47 (t, J = 7.1 Hz, 3H). ^{13}C NMR (75 MHz, $CDCl_3$+ DMSO-d_6) δ 168.92, 148.13, 141.90, 139.78, 133.30, 133.17, 132.09, 131.93, 131.81, 131.07, 128.13, 116.27, 65.03, 19.31. HRMS calcd for $C_{14} H_{12} O_2 N_2$ Cl [M + H]$^+$: 275.0582; found: 275.0575.

4-chloro-3H-pyrrolo[2,3-c]quinoline(6)

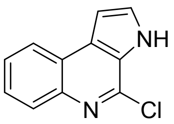

Following the general procedure (**Scheme 5**), compound **6** was obtained after column chromatography (hexane:EtOAc 7:3) in 70% as a creamy white solid. mp 260-262 °C.^{1}H NMR (400 MHz, $CDCl_3$) δ 9.10 (s, 1H), 8.20 – 8.15 (m, 1H), 8.14 – 8.07 (m, 1H), 7.65 – 7.57 (m, 2H), 7.50 – 7.45 (m, 1H), 7.11 (dd, J = 3.0, 2.1 Hz, 1H). ^{13}C NMR (101 MHz, $CDCl_3$) δ 142.60, 136.56, 130.51, 128.90, 126.88, 126.83, 126.50, 126.04, 123.33, 123.11. HRMS calcd for $C_{11} H_8 N_2$ Cl [M + H]$^+$: 203.0371; found: 203.0364.

4-(p-tolyl)-3H-pyrrolo[2,3-c]quinoline(7)

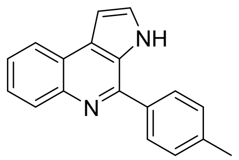

Following the general procedure (**Scheme 5**), compound **7** was obtained after column chromatography (hexane:EtOAc 7:3) in 80% as a creamy white solid. mp 258-260 °C. ^{1}H NMR (500 MHz, $CDCl_3$) δ 9.46 (s, 1H), 8.23 (d, J = 7.3 Hz, 1H), 8.19 (d, J = 7.5 Hz, 1H), 7.77 (d, J = 7.5 Hz, 2H), 7.58 (t, J = 7.1 Hz, 2H), 7.44 (d, J = 2.7 Hz, 1H), 7.27 – 7.22 (m, 2H), 7.08 (d, J = 2.8 Hz, 1H), 2.35 (s, 3H). ^{13}C NMR (101 MHz, $CDCl_3$) δ 146.63, 142.08, 139.65, 134.46, 129.80, 129.63, 129.44, 129.18, 128.70, 128.38, 127.25, 126.53, 125.95, 123.02, 122.94, 102.14. HRMS calcd for $C_{18} H_{15} N_2$ [M + H]$^+$: 259.1230; found: 259.1222.

4-chloro-3H-pyrrolo[2,3-c]quinoline-1-carboxylic acid(8)

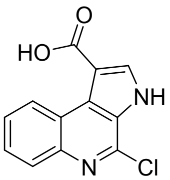

Following the general procedure (**Scheme 5**), compound **8** was obtained after column chromatography (hexane:EtOAc 5:5) in 76% as a creamy white solid. mp 358-360 °C.^{1}H NMR (400 MHz, DMSO-d_6) δ 13.14 (s, 1H), 12.63 (s, 1H), 9.70 (dd, J = 6.8, 2.8 Hz, 1H), 8.34 (s, 1H), 8.02 (dd, J = 6.8, 2.6 Hz, 1H), 7.75 – 7.65 (m, 2H). ^{13}C NMR (101 MHz, DMSO-d_6) δ 164.77, 142.27, 136.15, 135.19, 127.80, 127.34, 126.81, 126.24, 125.75, 122.41, 111.21. HRMS calcd for $C_{12} H_8 O_2 N_2$ Cl [M + H]$^+$: 247.0269; found: 247.0264.

^{1}H-NMR of 3aa:

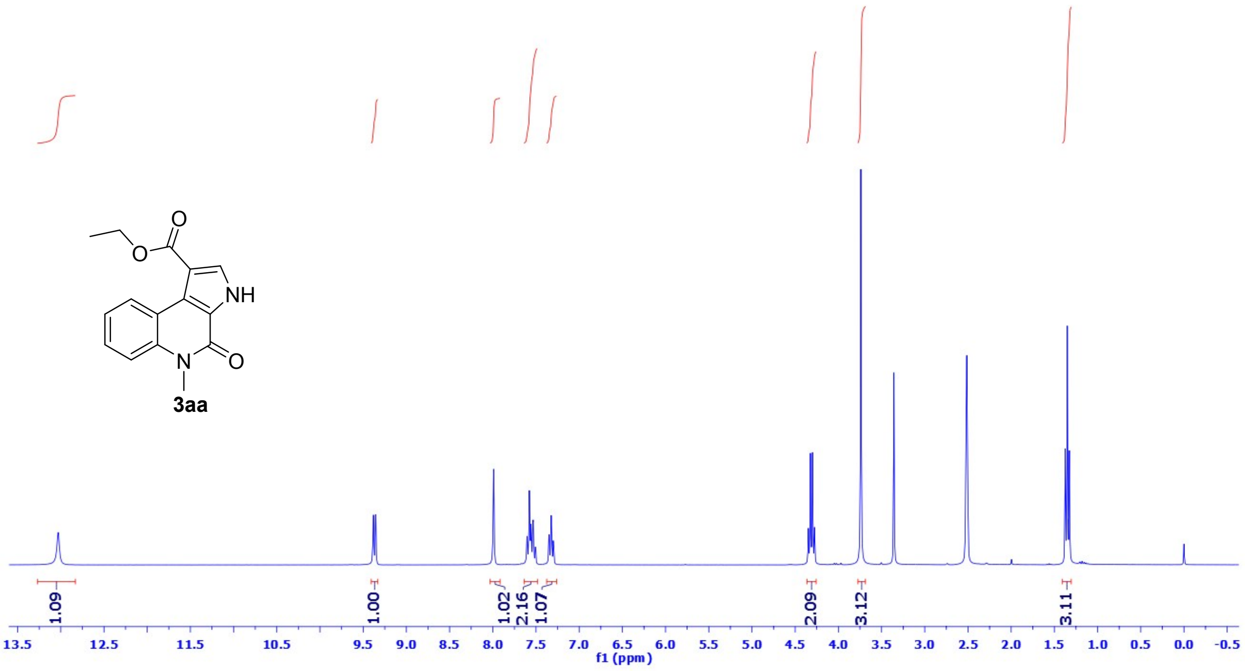

^{13}C-NMR of 3aa:

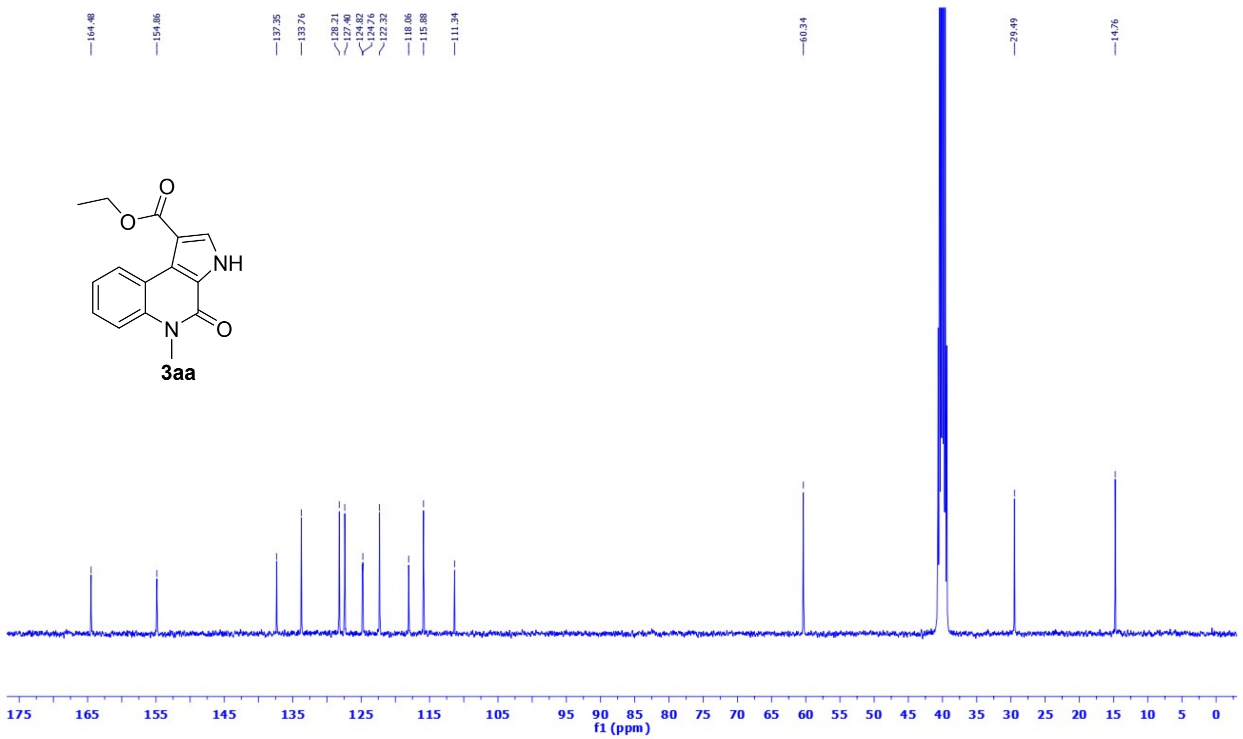

^{1}H-NMR of 3ab:

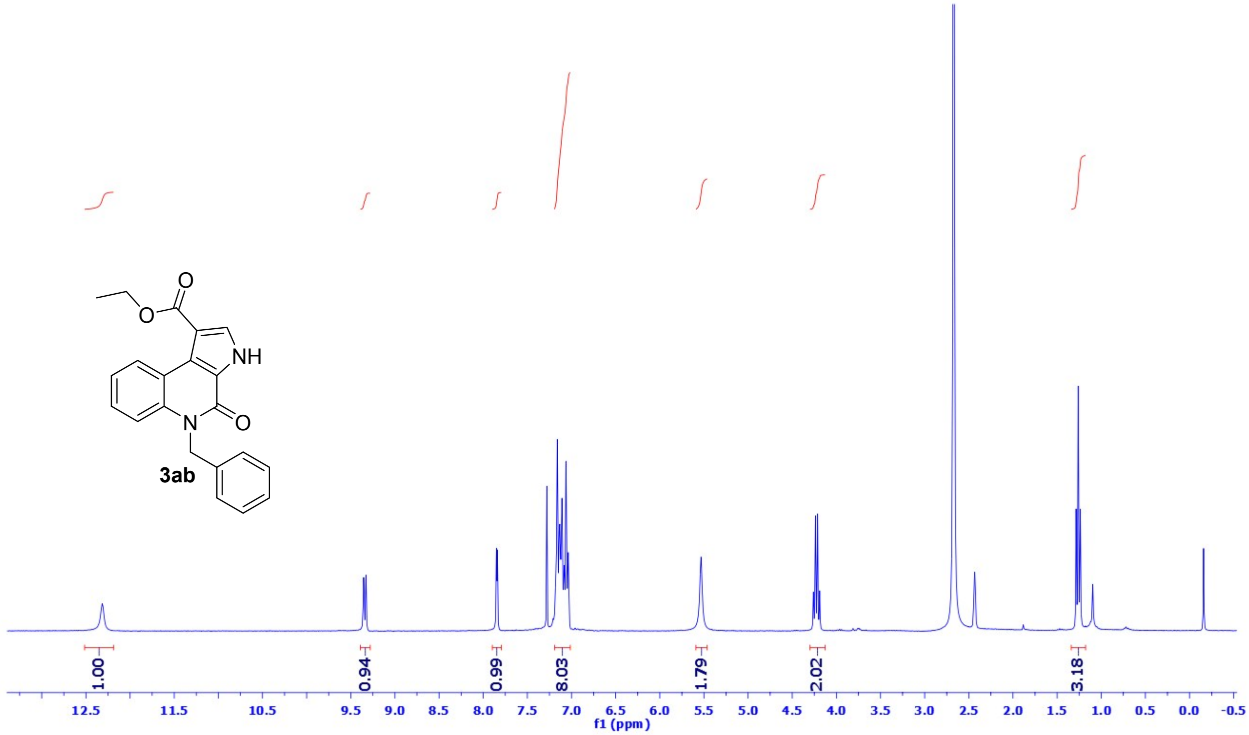

^{13}C-NMR of 3ab:

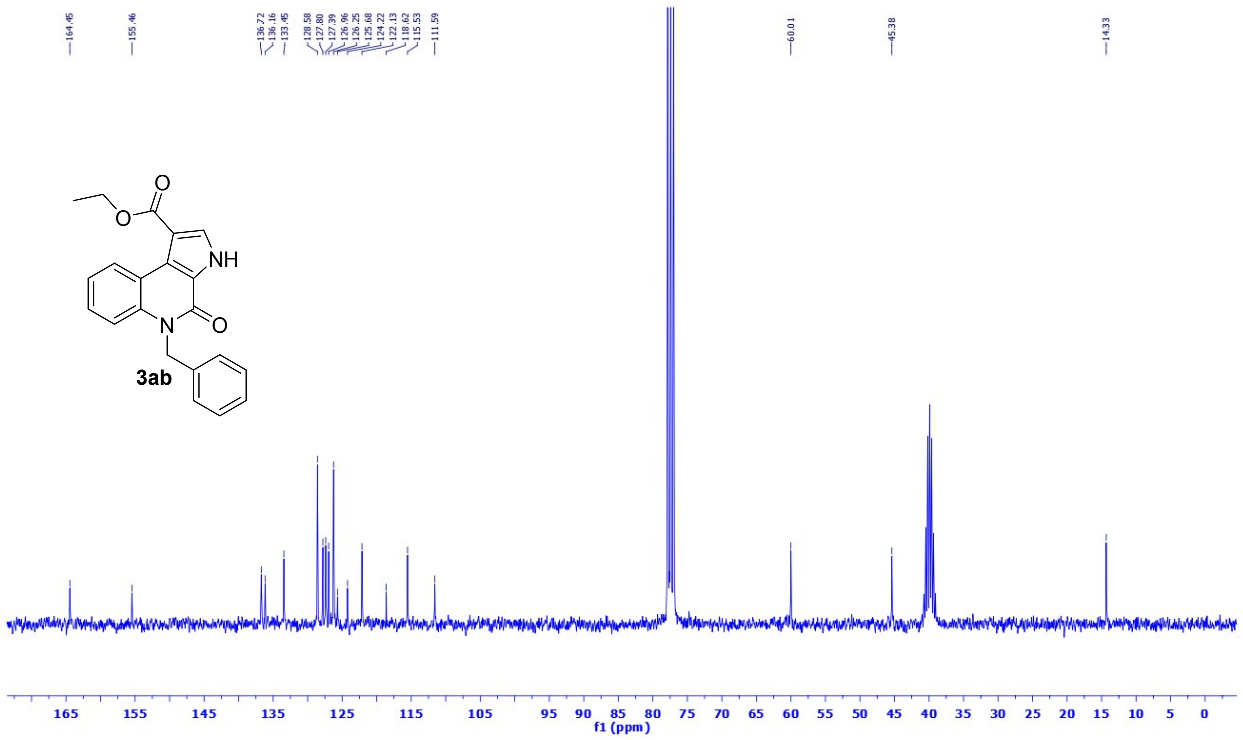

¹H-NMR of 3ac:

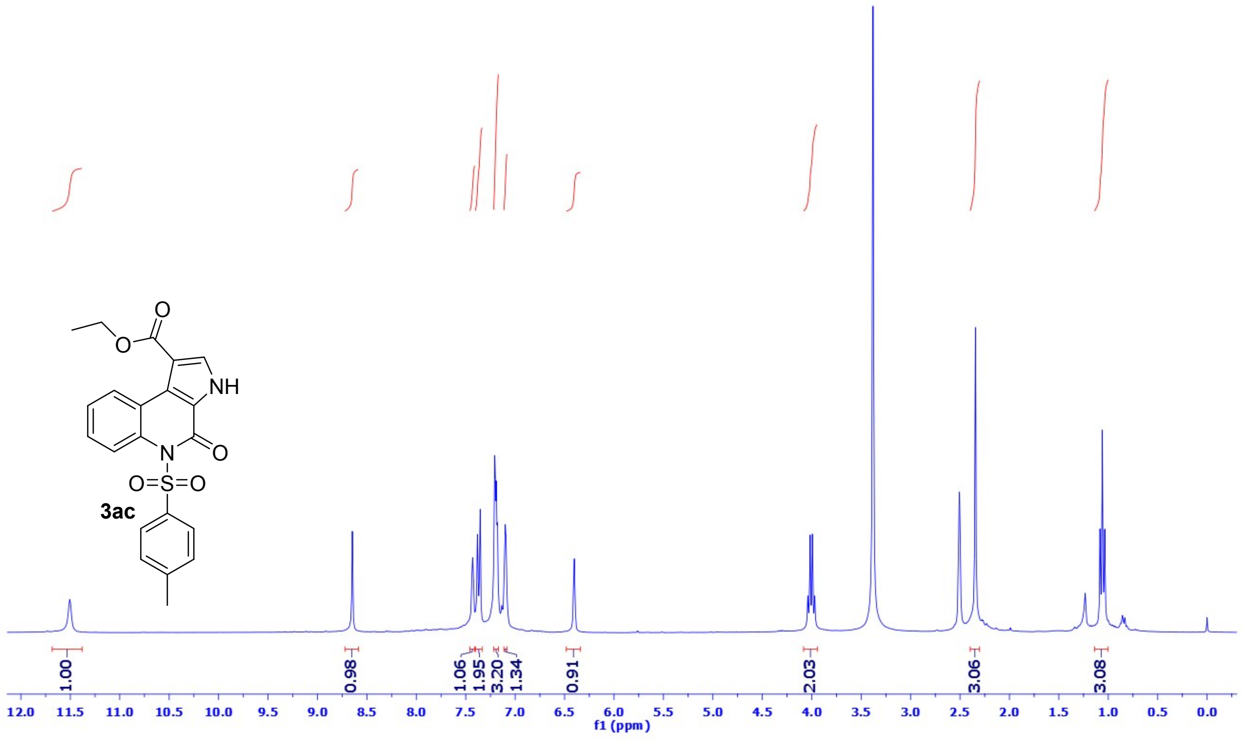

¹³C-NMR of 3ac:

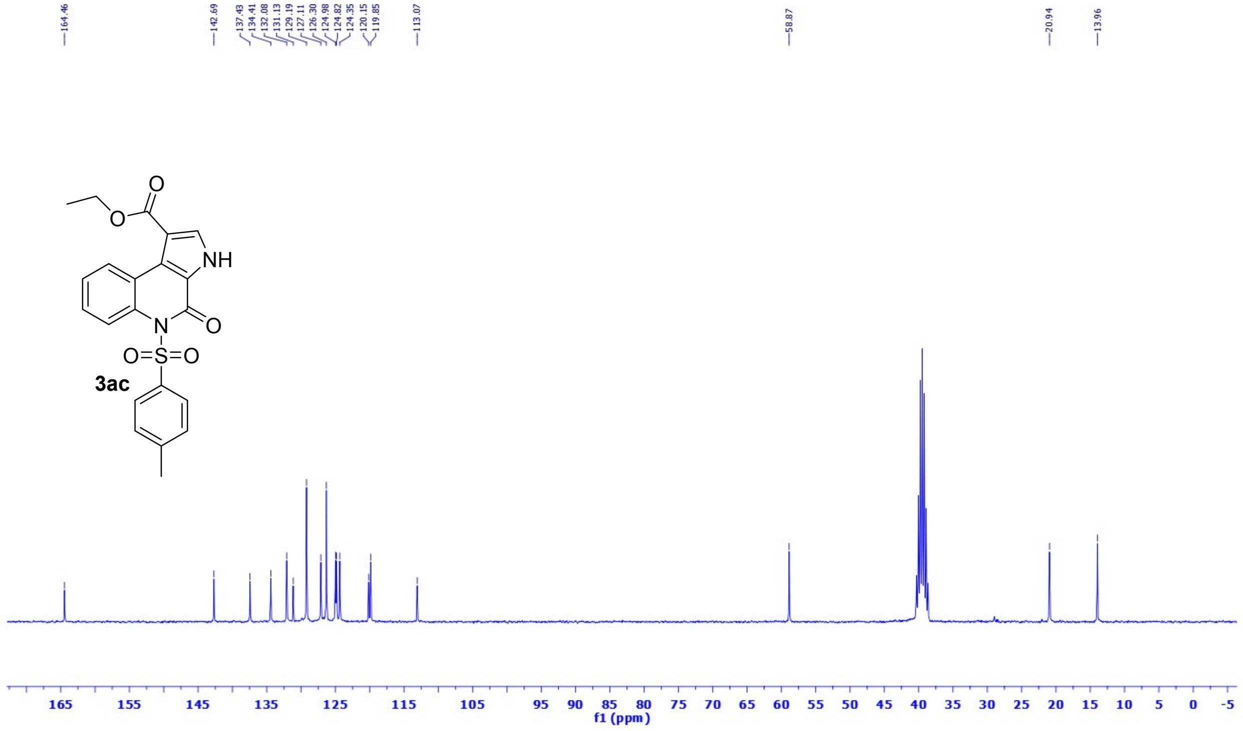
164.46
142.69
137.43
134.41
132.08
131.13
129.19
127.11
126.30
124.98
124.82
124.35
120.15
119.85
113.07
58.87
20.94
13.96
3ac
f1 (ppm)

^{1}H-NMR of 3ba:

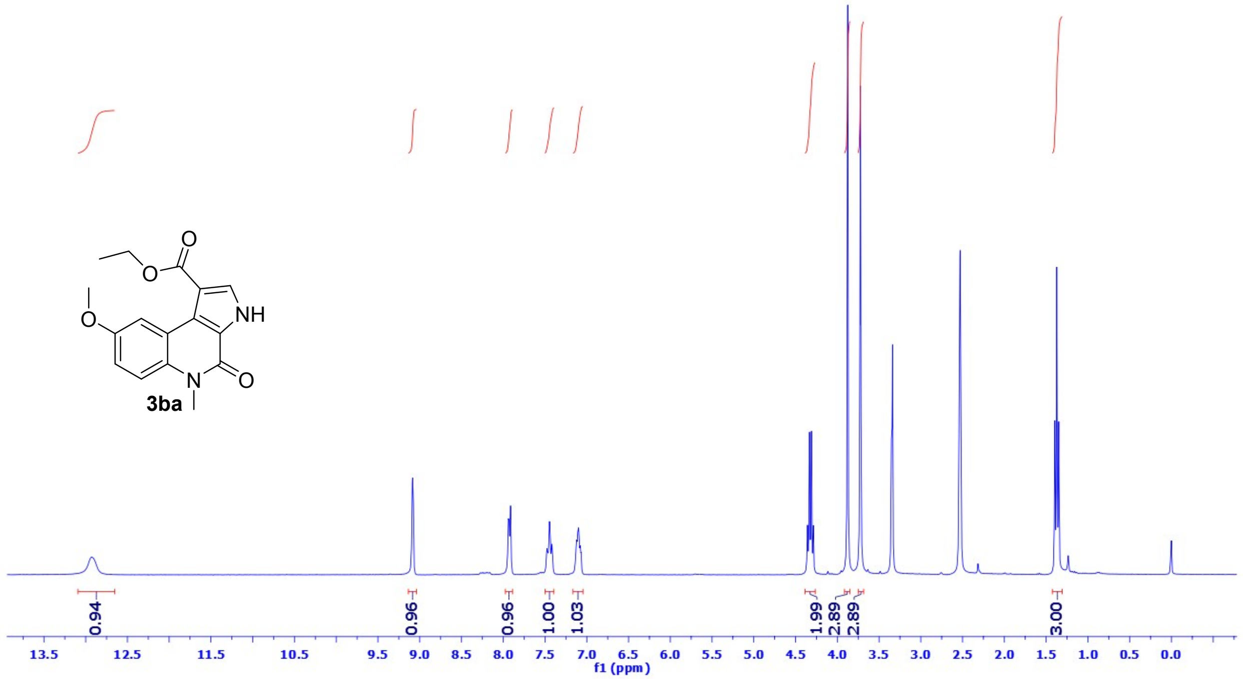
3ba
0.94
0.96
0.96
1.00
1.03
1.99
2.89
2.89
3.00
f1 (ppm)

^{13}C-NMR of 3ba:

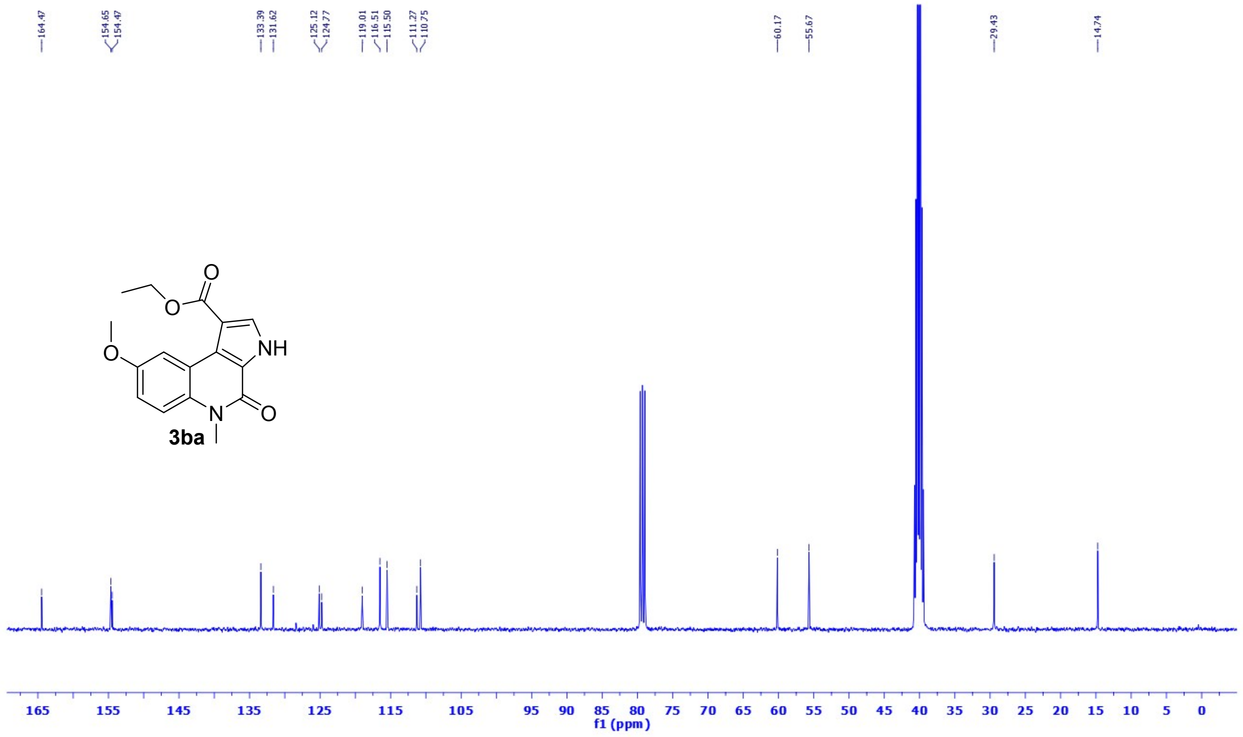

^{1}H-NMR of 3bb:

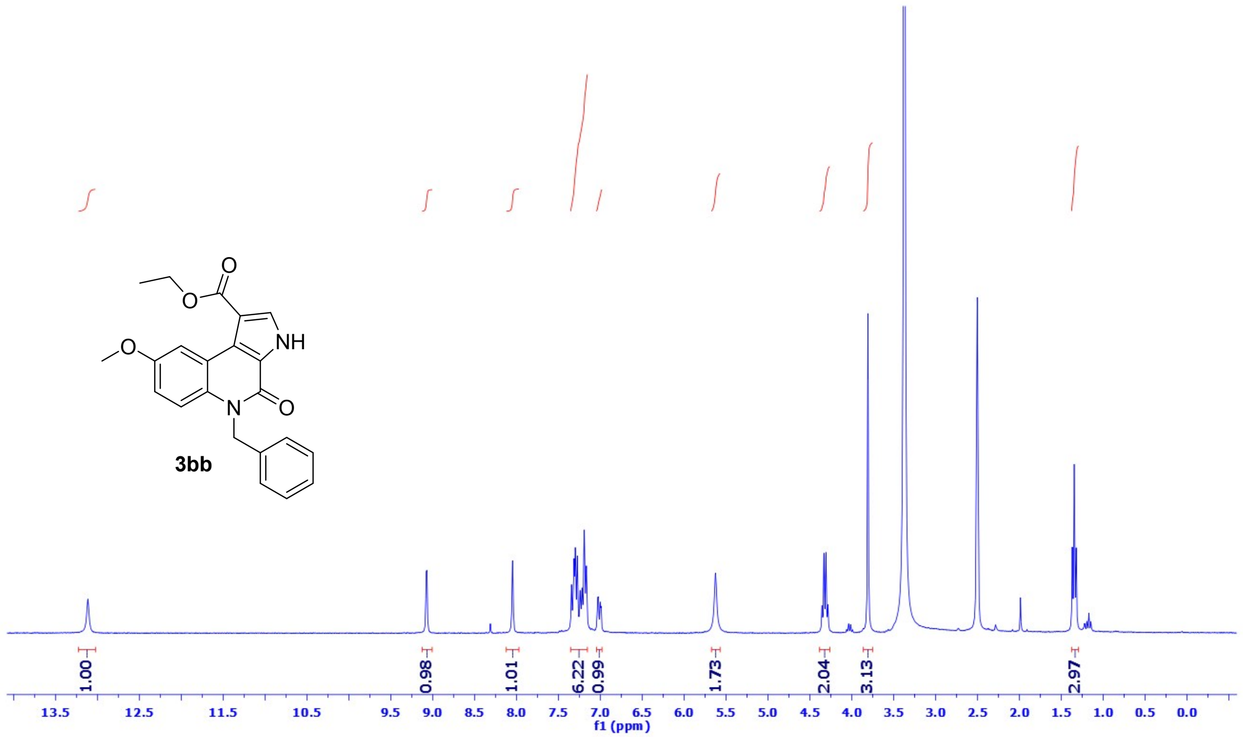

^{13}C-NMR of 3bb:

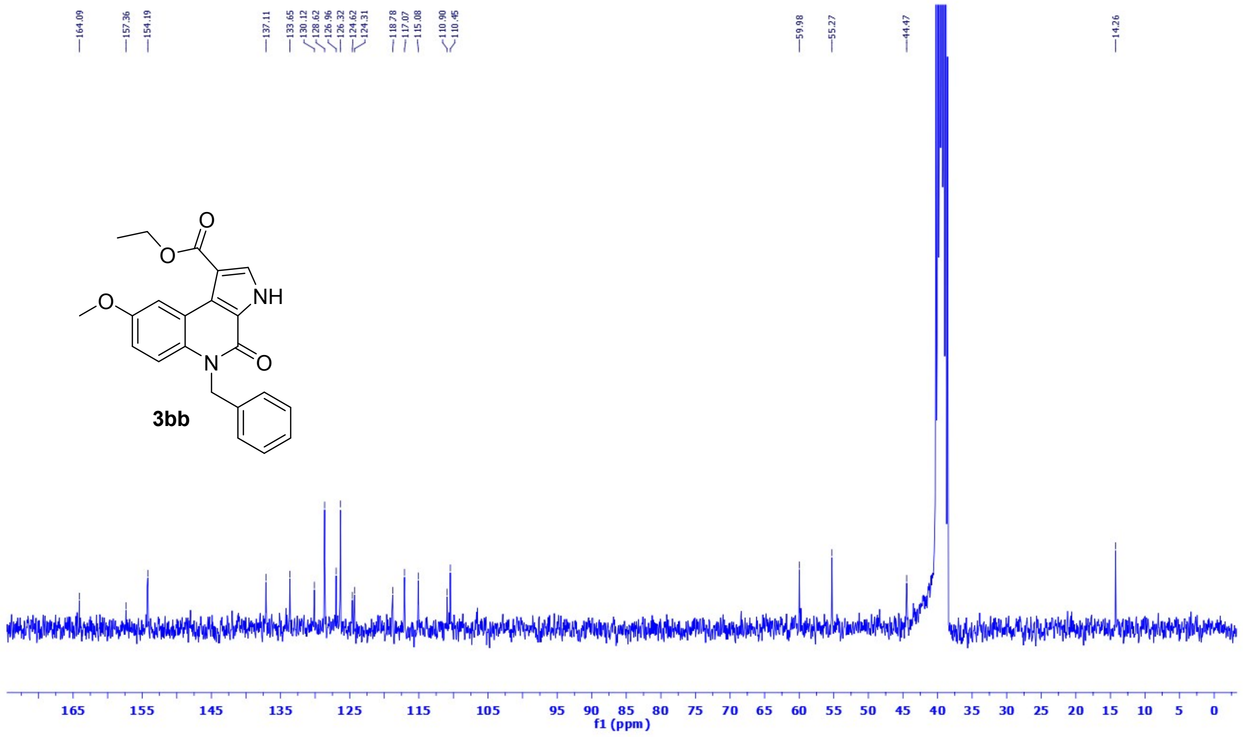

^{1}H-NMR of 3bc:

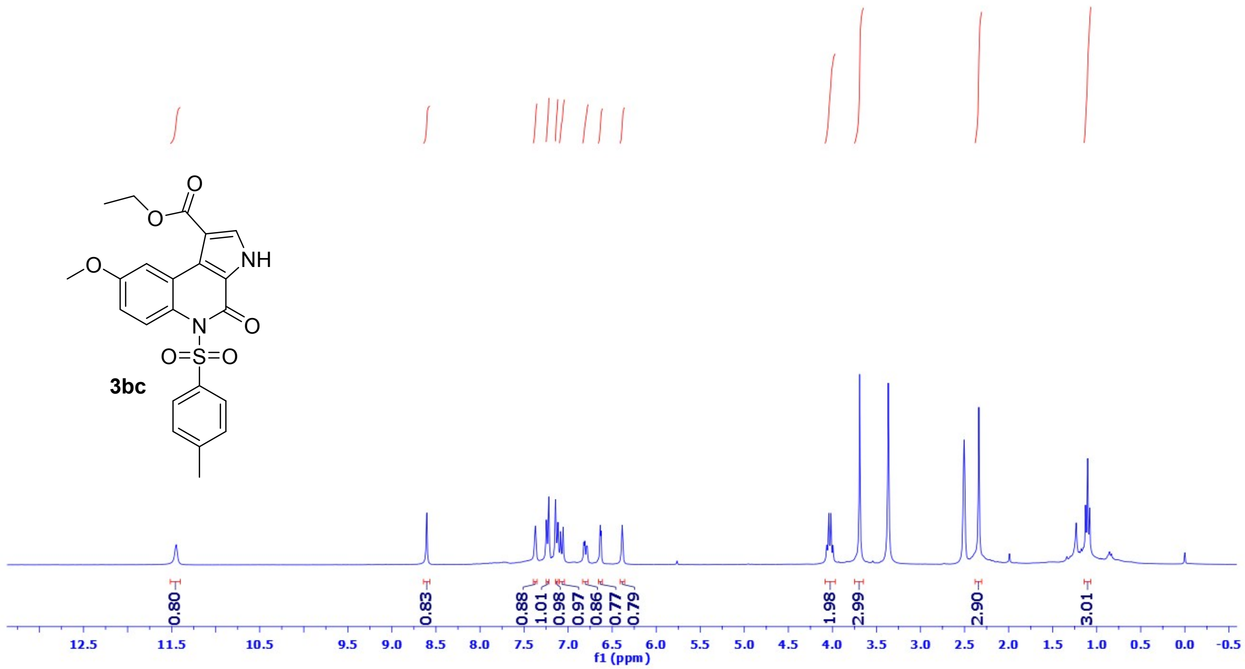

^{13}C-NMR of 3bc:

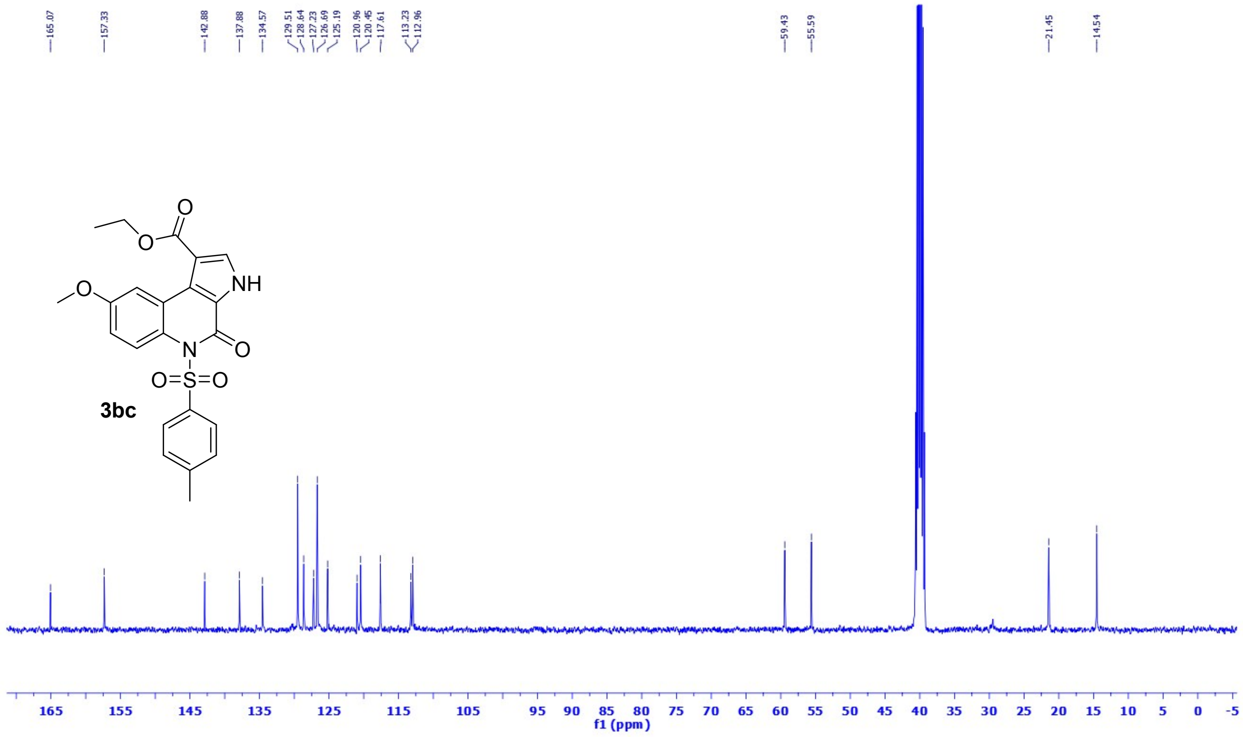

^{1}H-NMR of 3ca:

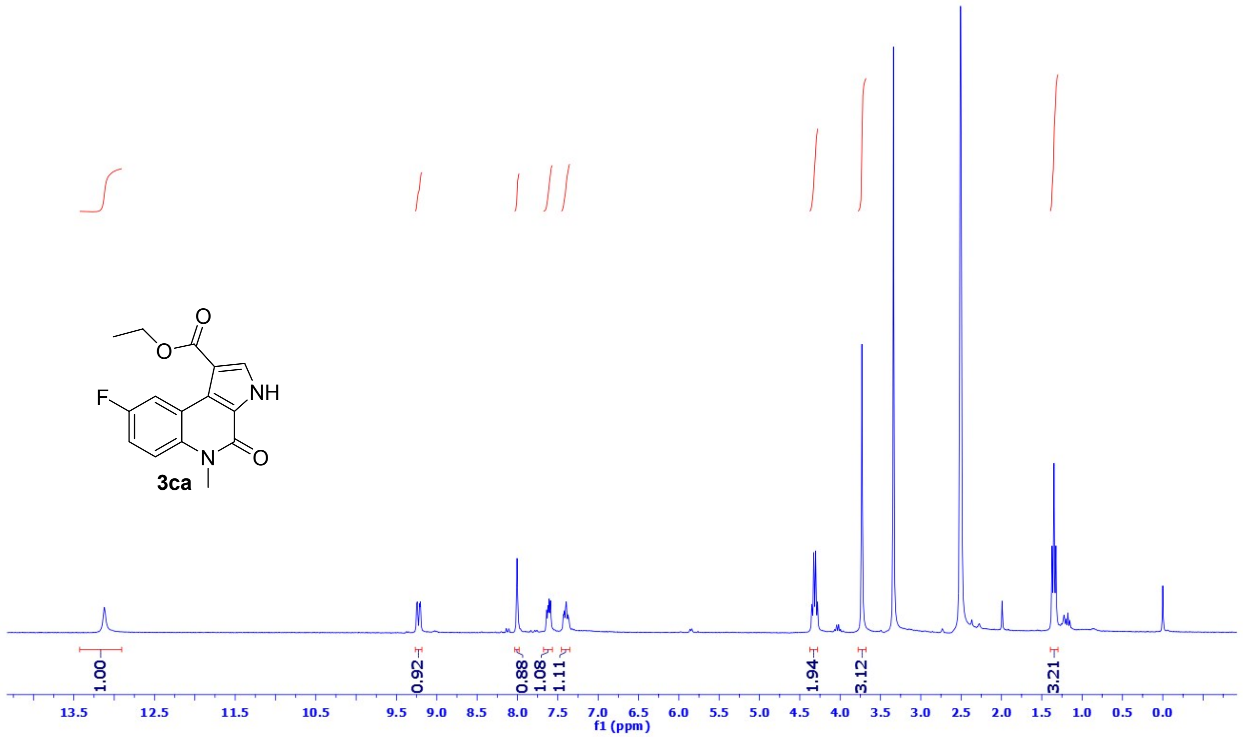

^{13}C-NMR of 3ca:

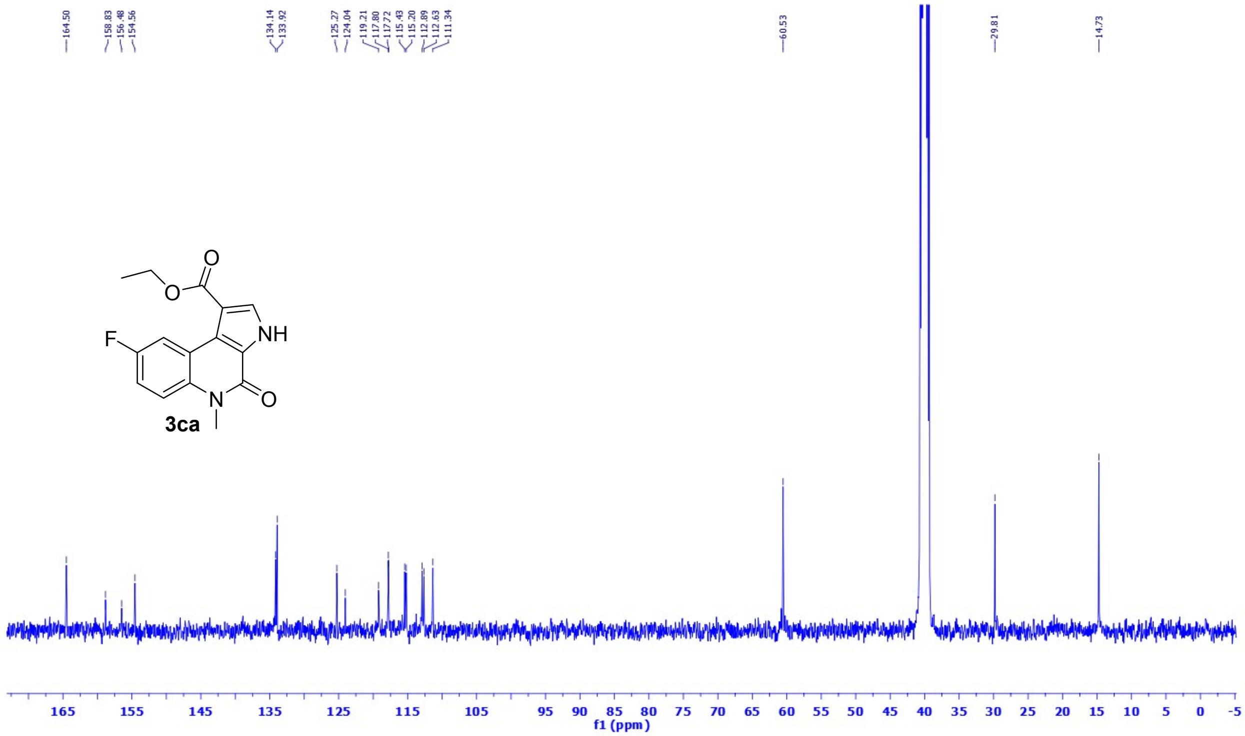

^{1}H-NMR of 3cb:

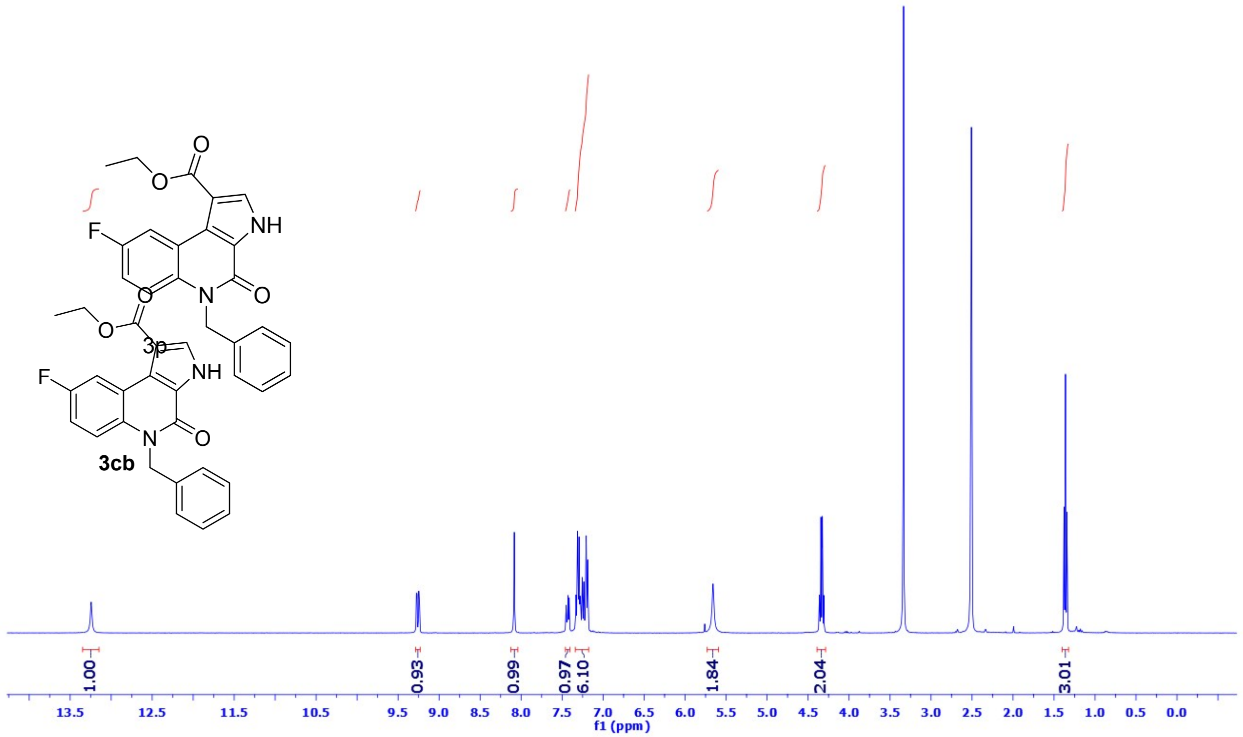

^{13}C-NMR of 3cb:

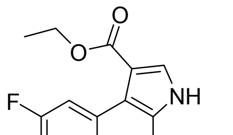

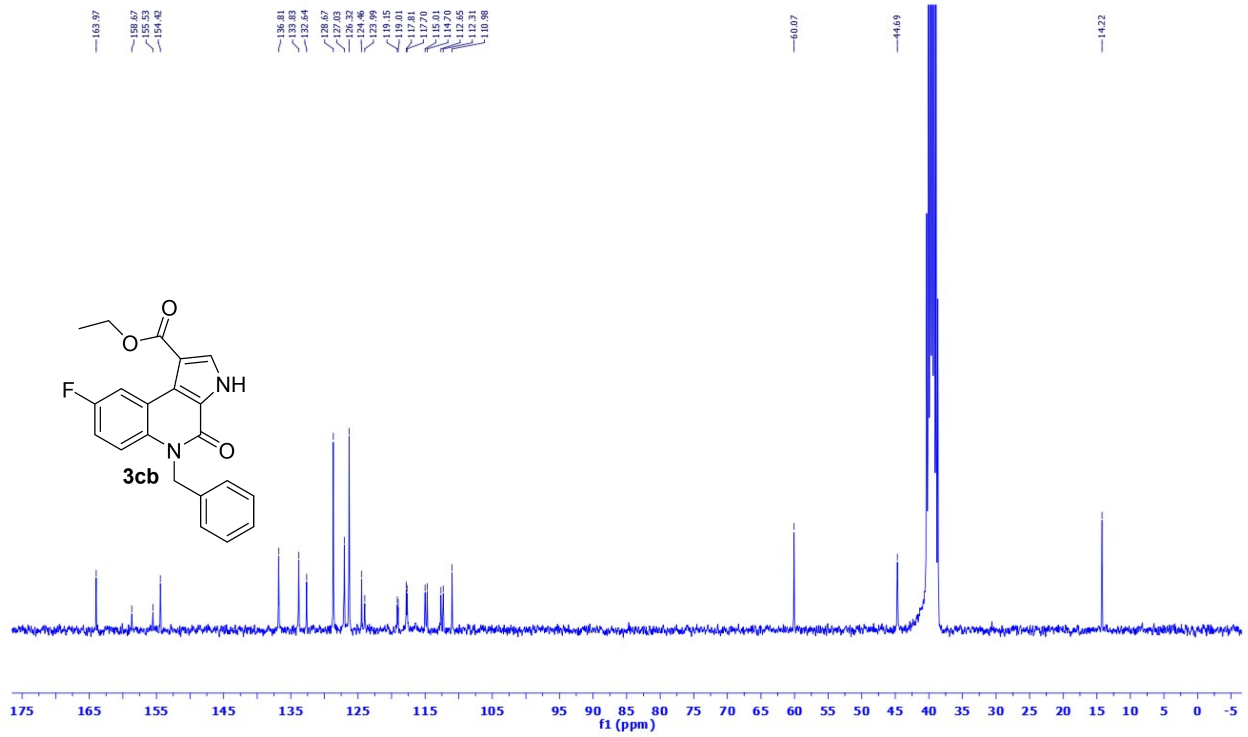

^{1}H-NMR of 3cc:

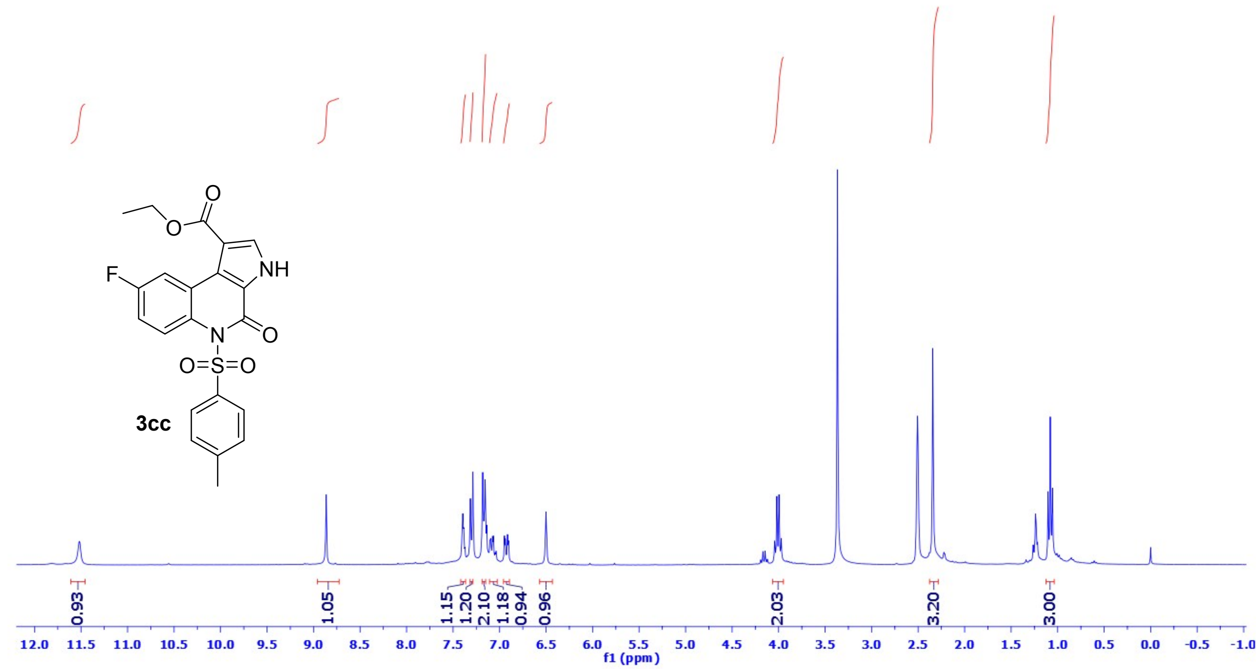

^{13}C-NMR of 3cc:

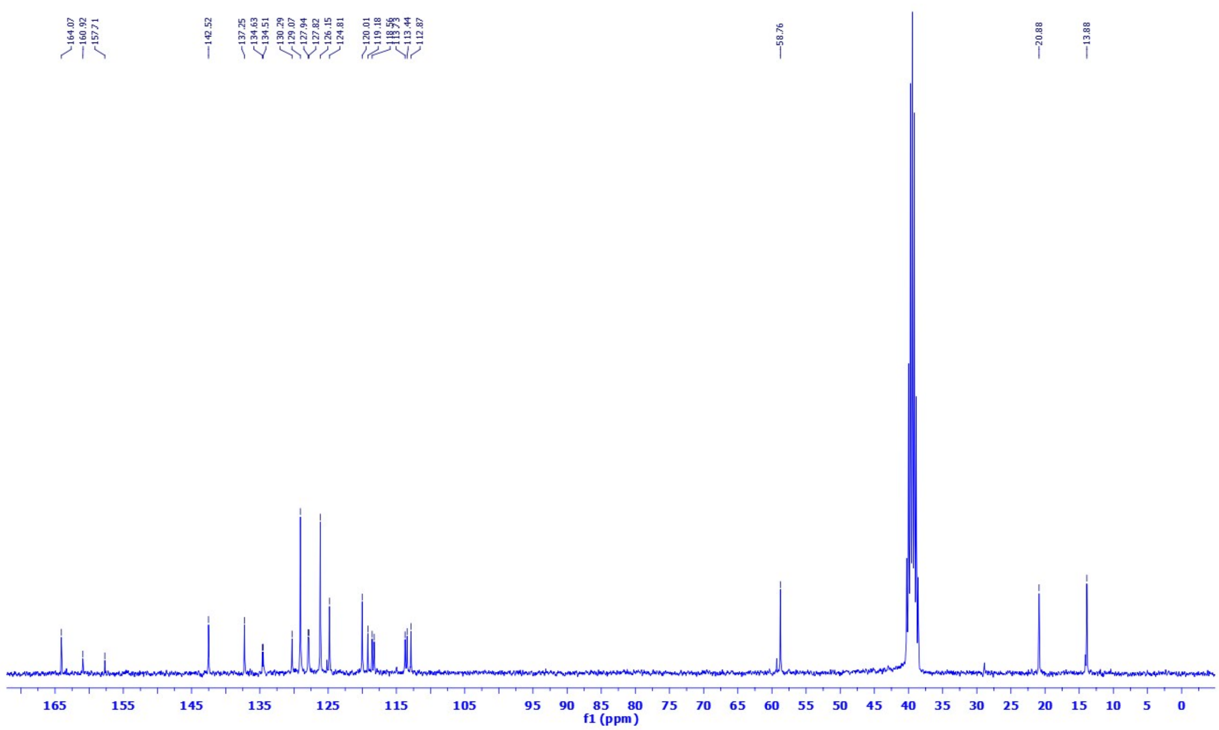

^{1}H-NMR of 3da:

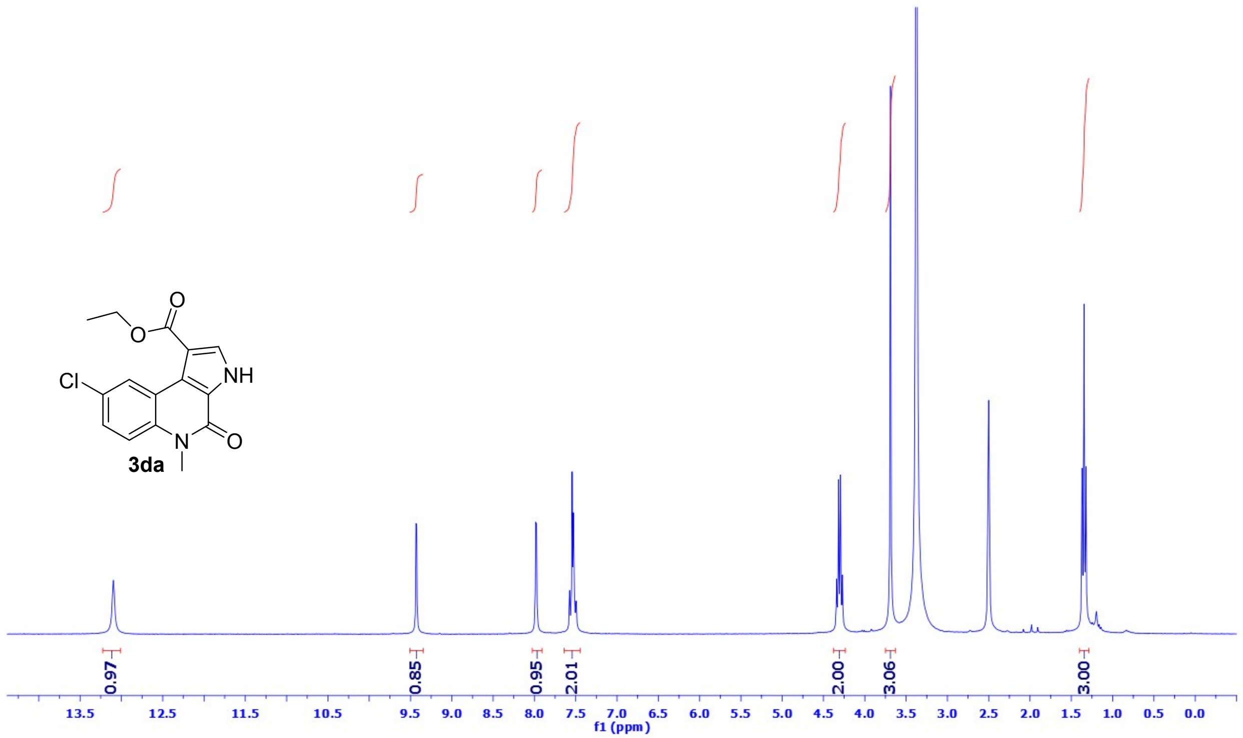

^{13}C-NMR of 3da:

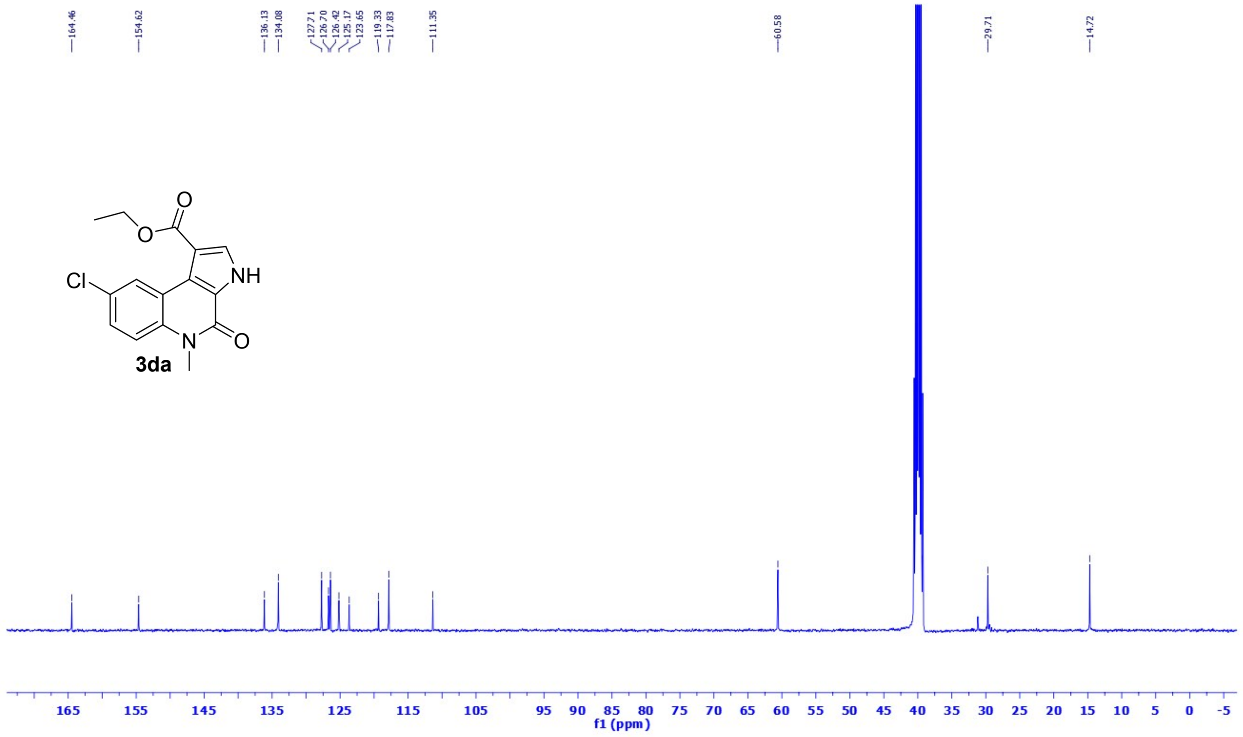

^{1}H-NMR of 3db:

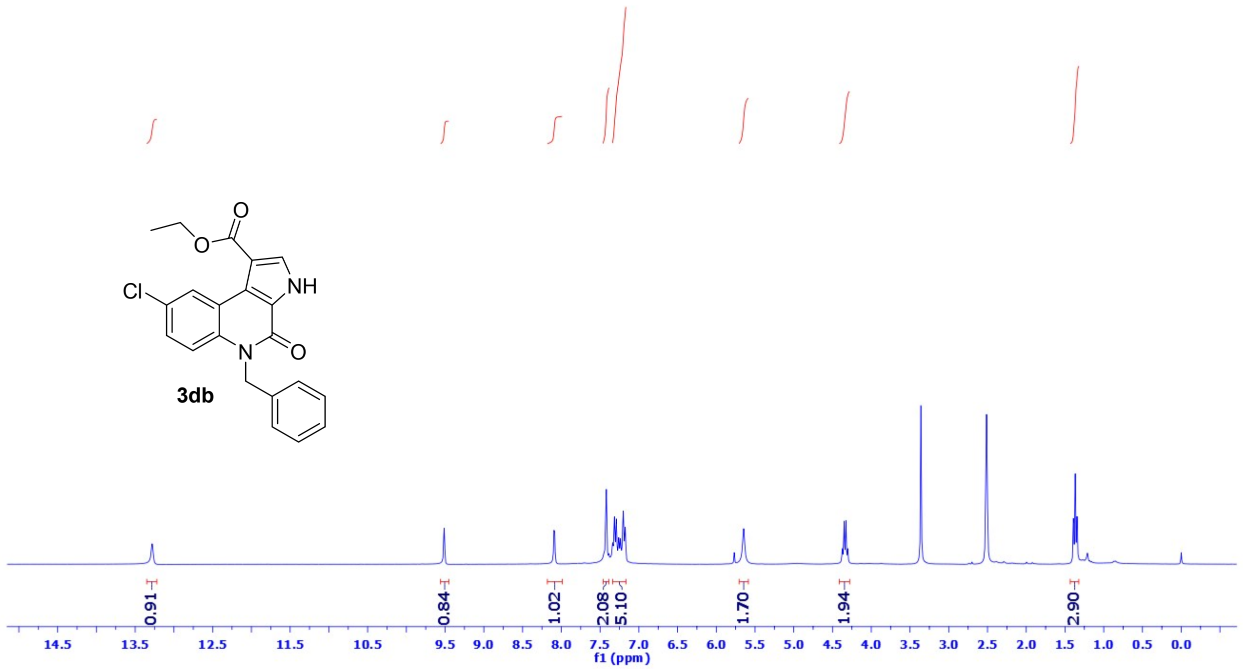

^{13}C-NMR of 3db:

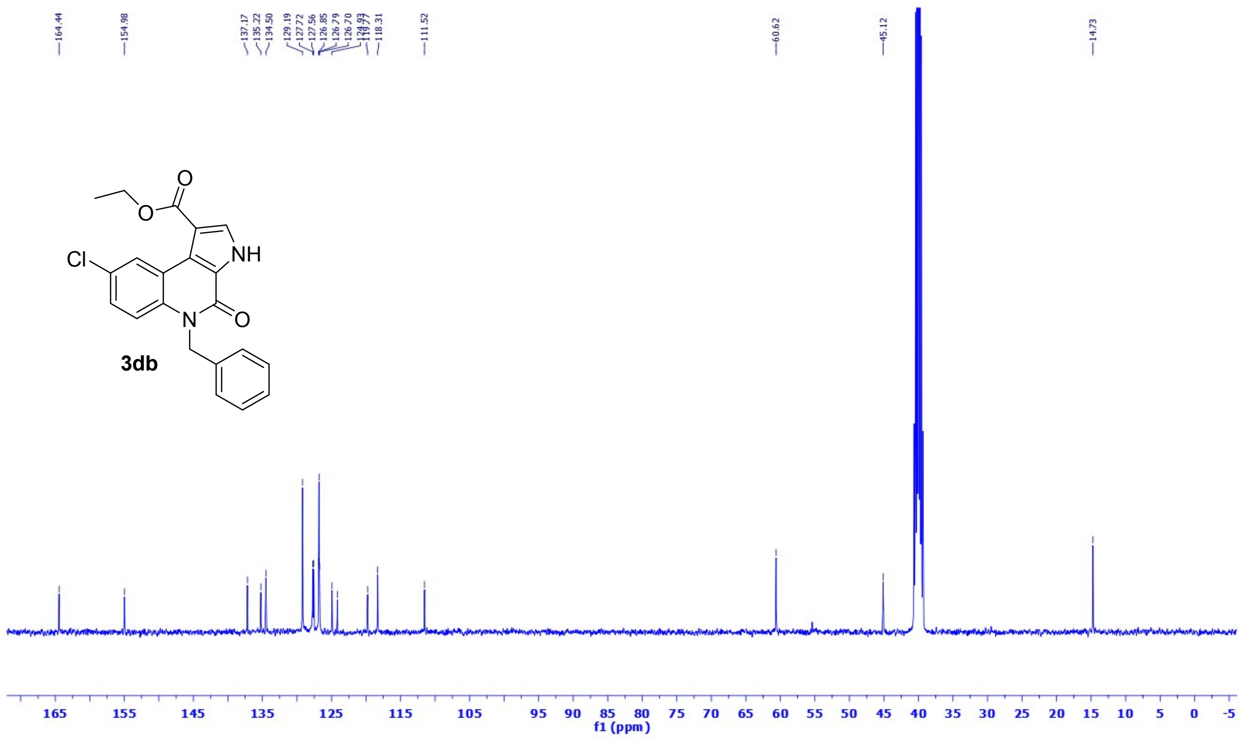

^{1}H-NMR of 3dc:

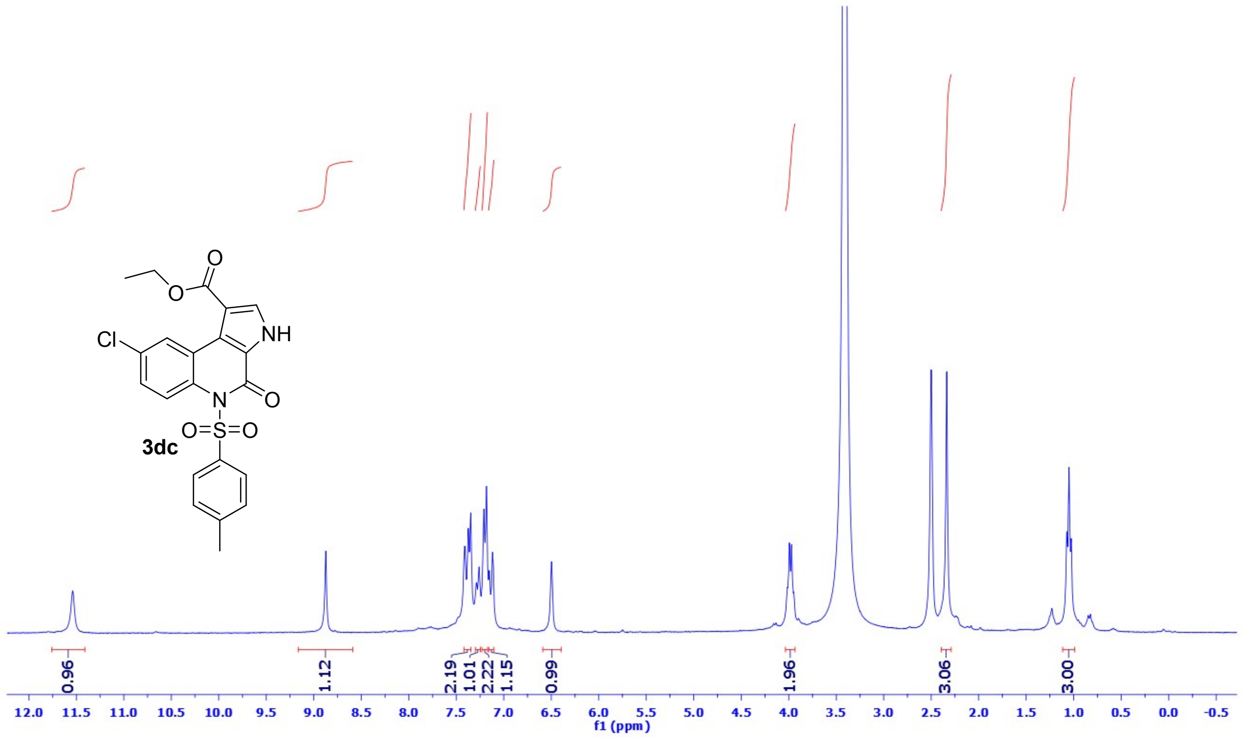

^{13}C-NMR of 3dc:

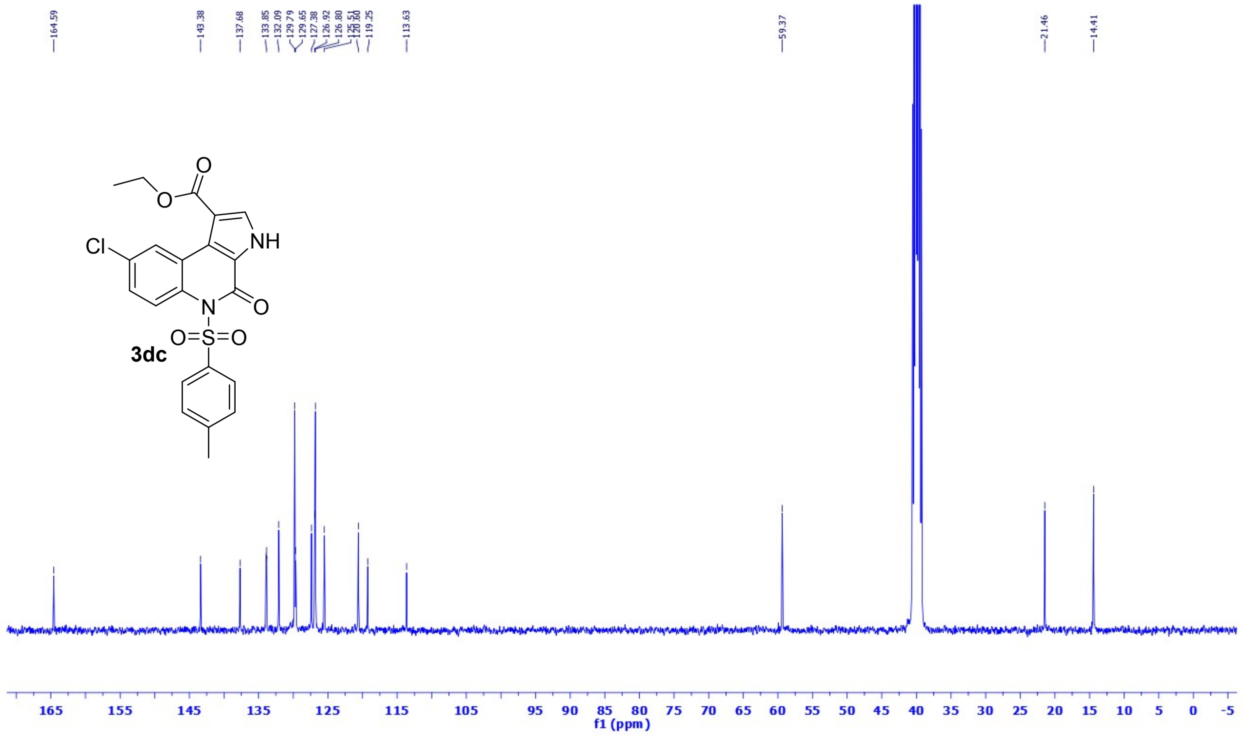

^{1}H-NMR of 3ea:

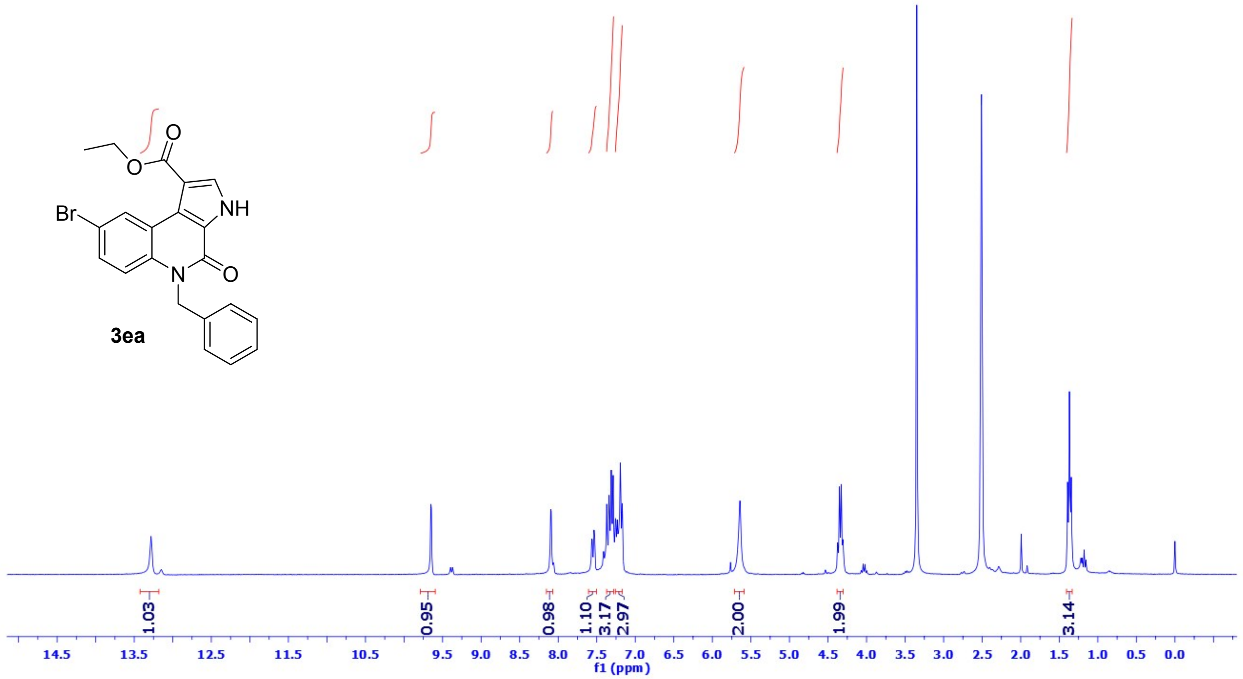

^{13}C-NMR of 3ea:

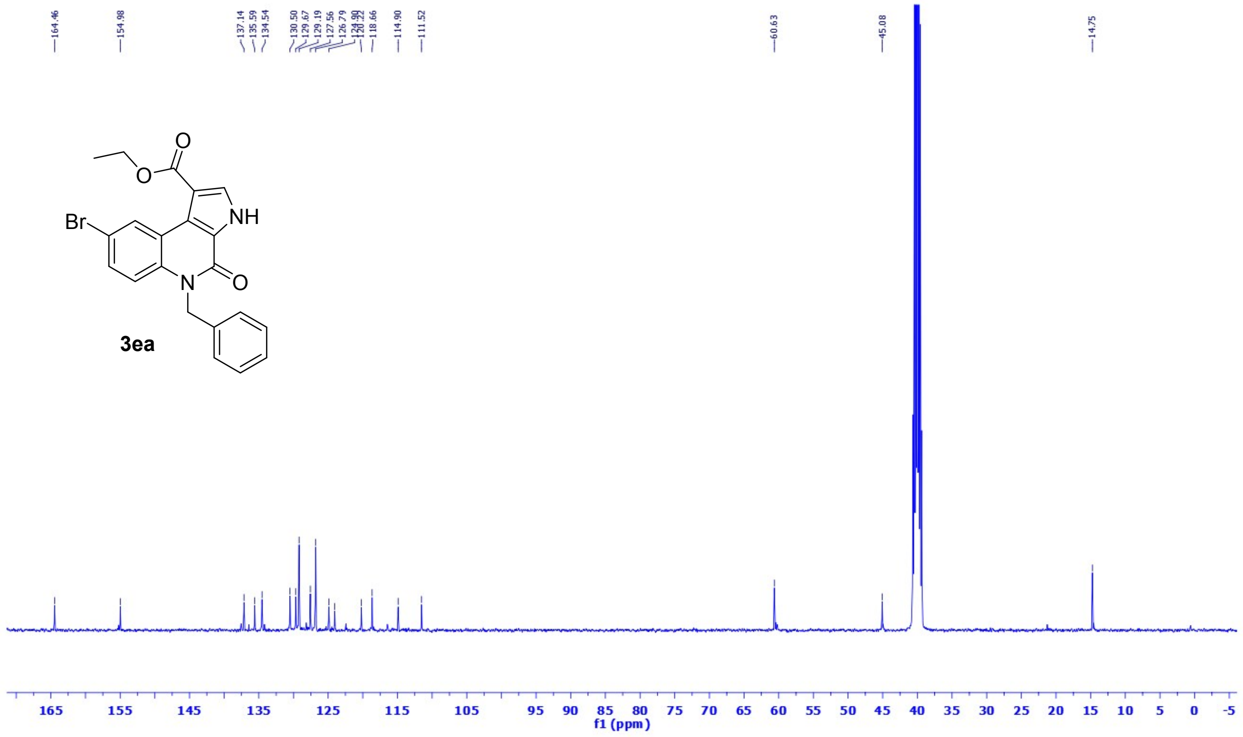

^{1}H-NMR of 3fa:

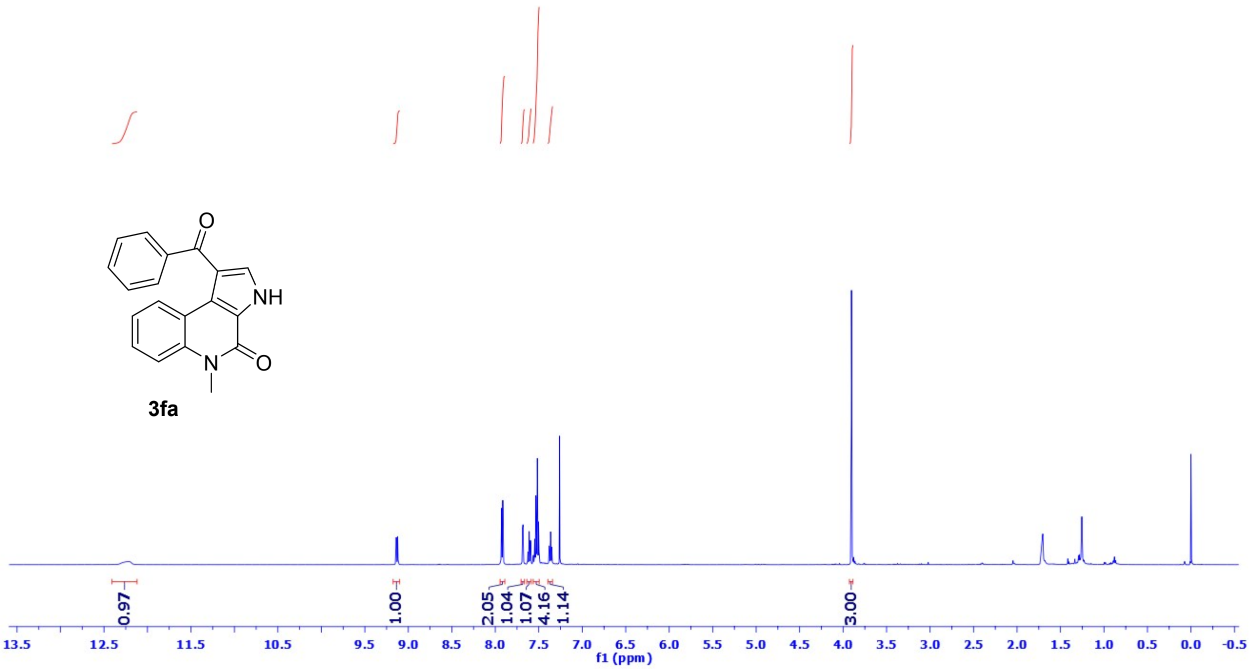

^{13}C-NMR of 3fa:

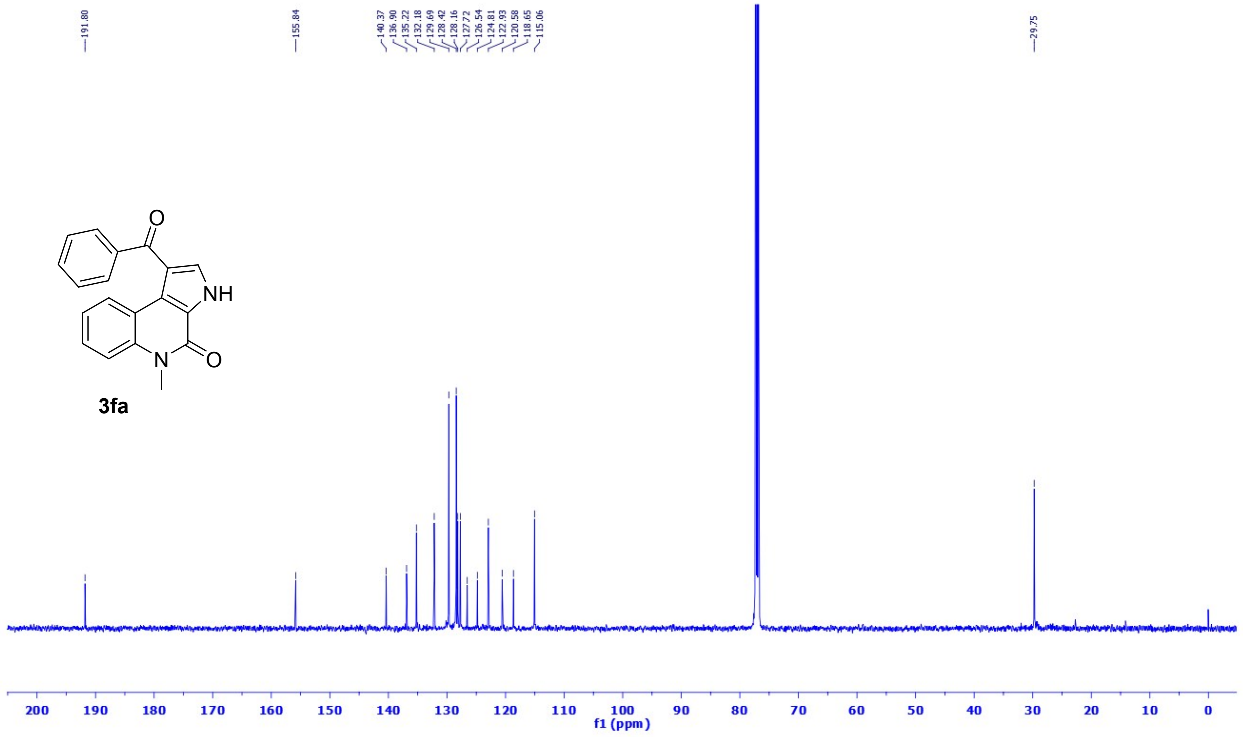

^{1}H-NMR of 3fb:

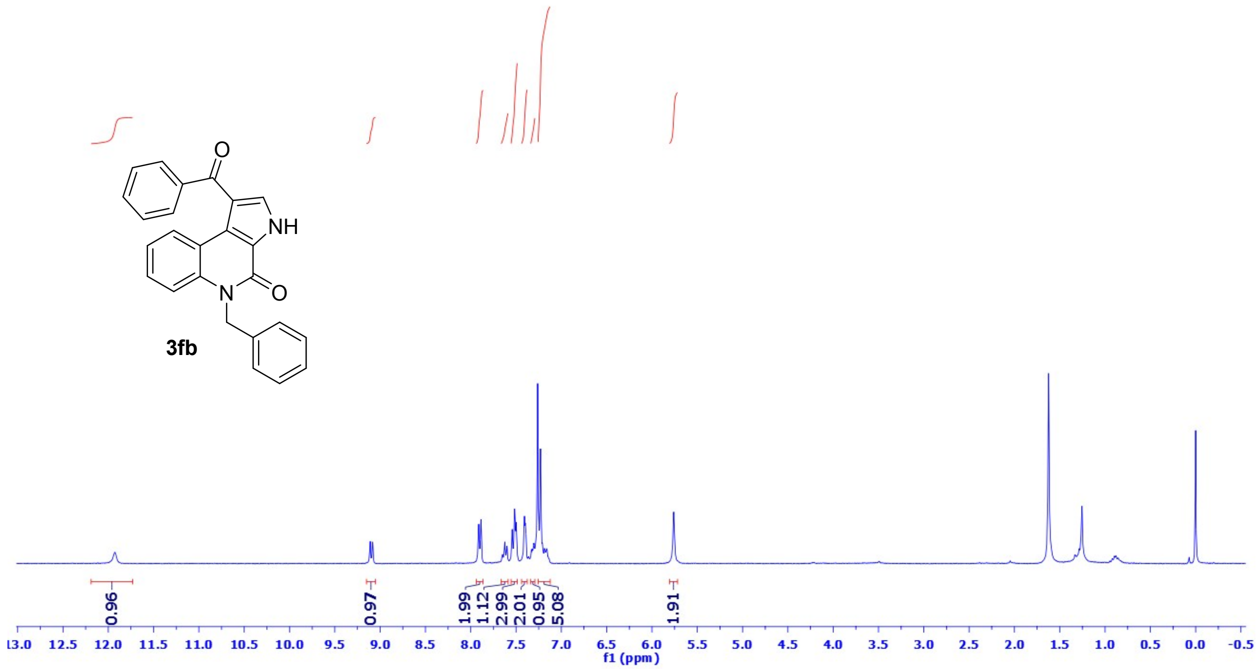

^{13}C-NMR of 3fb:

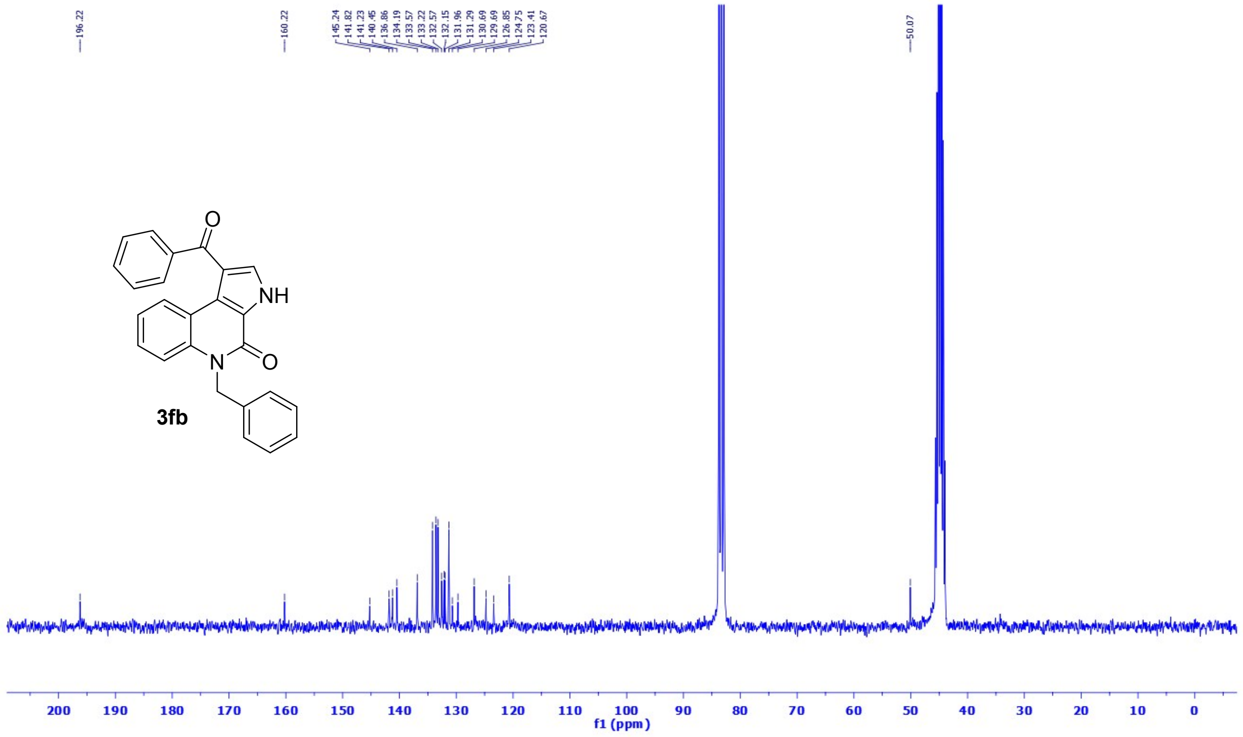

^{1}H-NMR of 3ga:

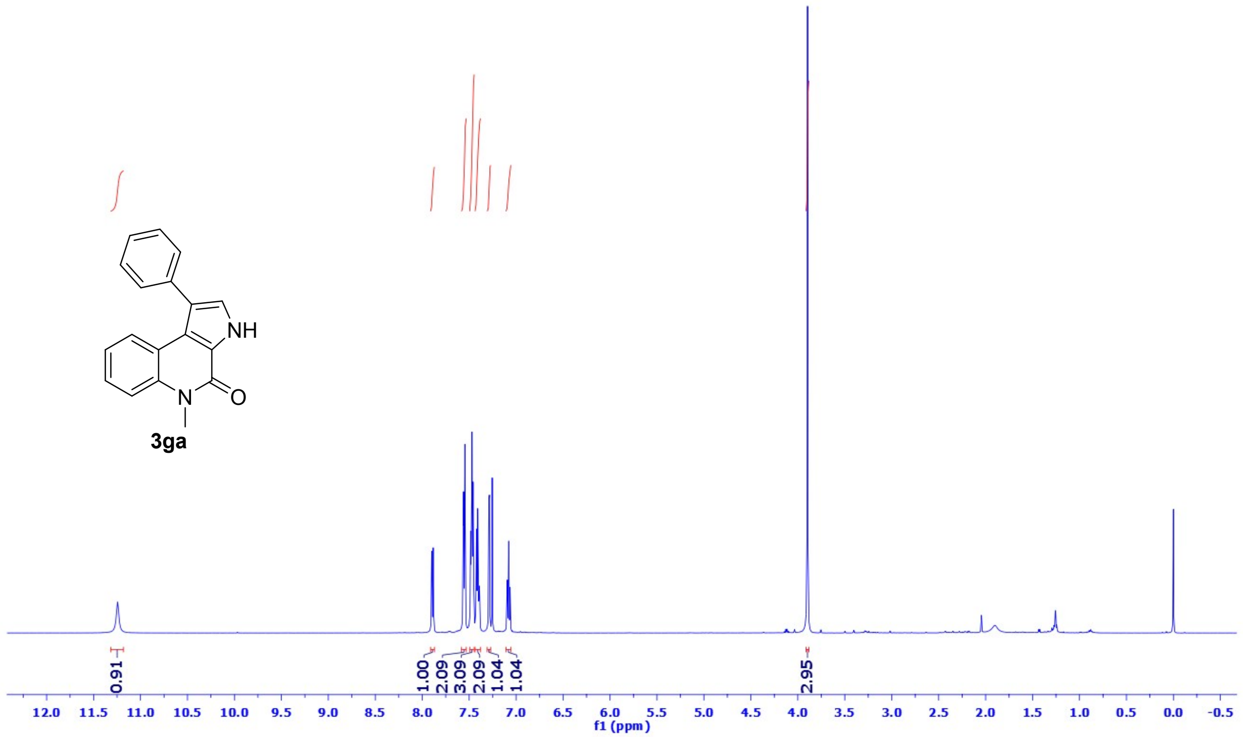

^{13}C-NMR of 3ga:

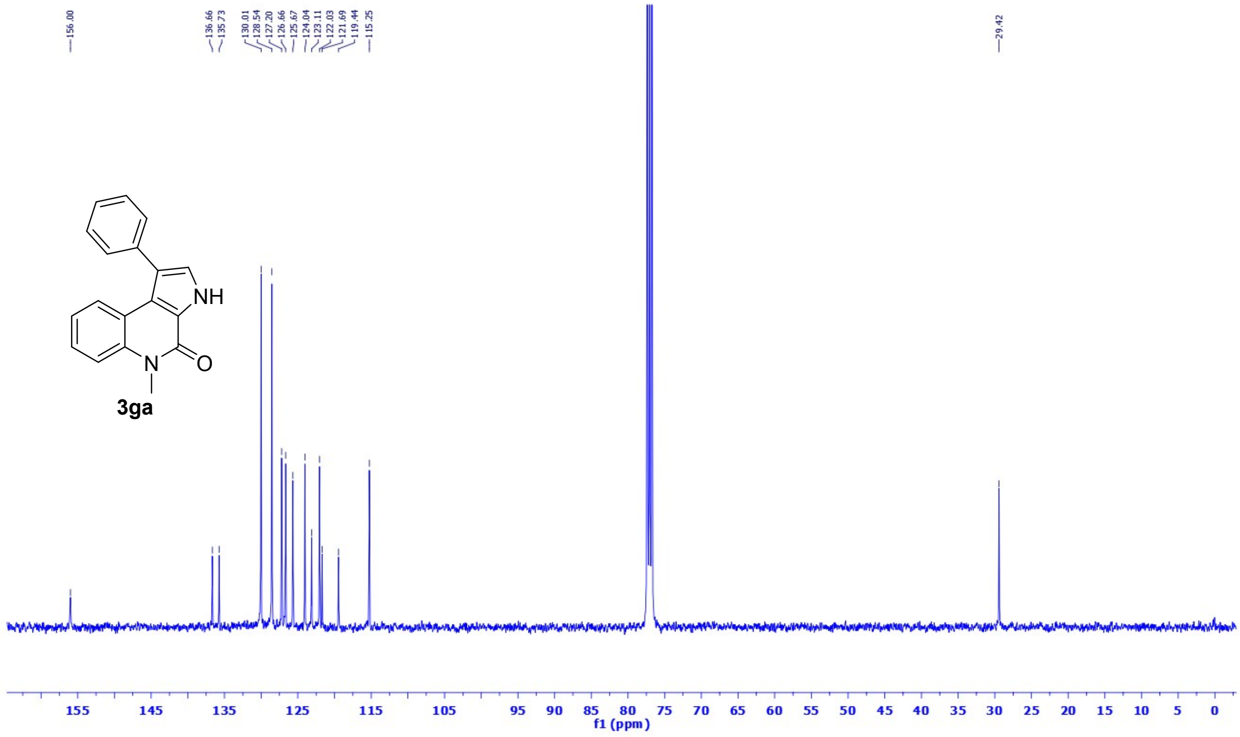
156.00
136.66
135.73
130.01
128.54
127.20
126.66
125.67
124.04
123.11
122.03
121.69
119.44
115.25
29.42
NH
N
O
3ga
155 145 135 125 115 105 95 90 85 80 75 70 65 60 55 50 45 40 35 30 25 20 15 10 5 0
f1 (ppm)

^{1}H-NMR of 4aa:

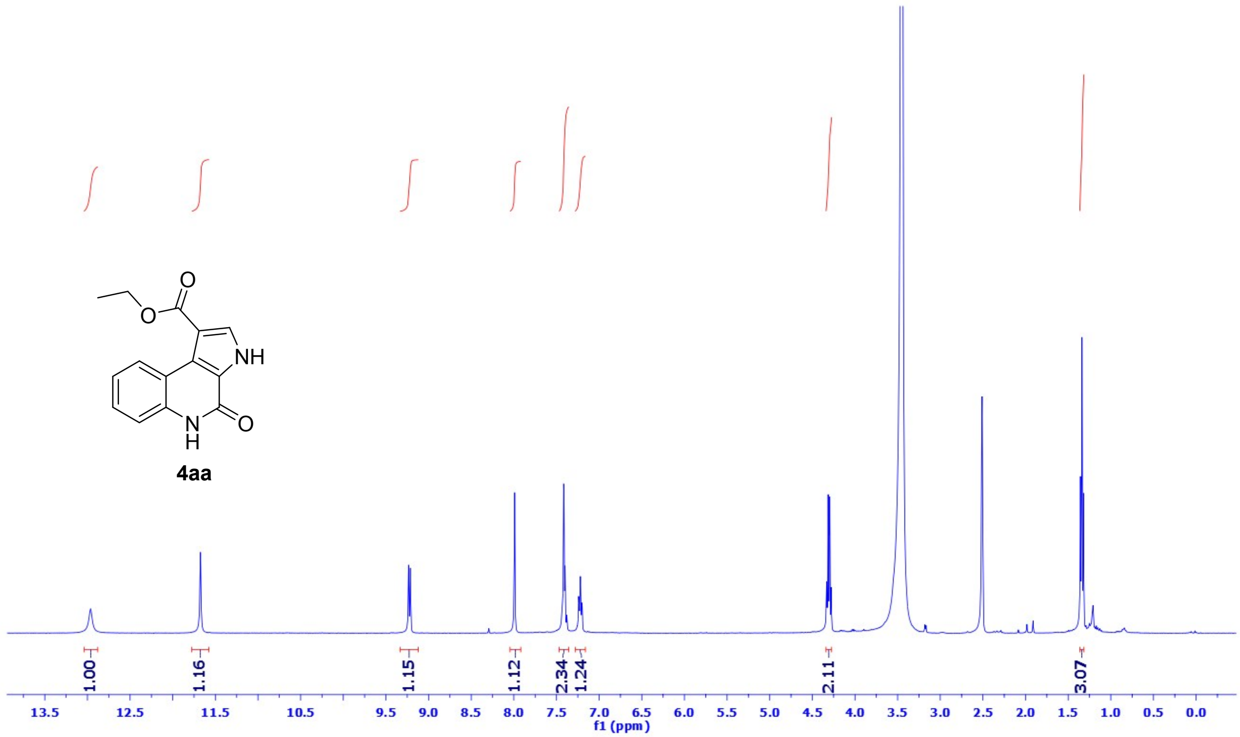
O
O
NH
N
H
O
4aa
1.00
1.16
1.15
1.12
2.34
1.24
2.11
3.07
13.5 12.5 11.5 10.5 9.5 9.0 8.5 8.0 7.5 7.0 6.5 6.0 5.5 5.0 4.5 4.0 3.5 3.0 2.5 2.0 1.5 1.0 0.5 0.0
f1 (ppm)

^{13}C-NMR of 4aa:

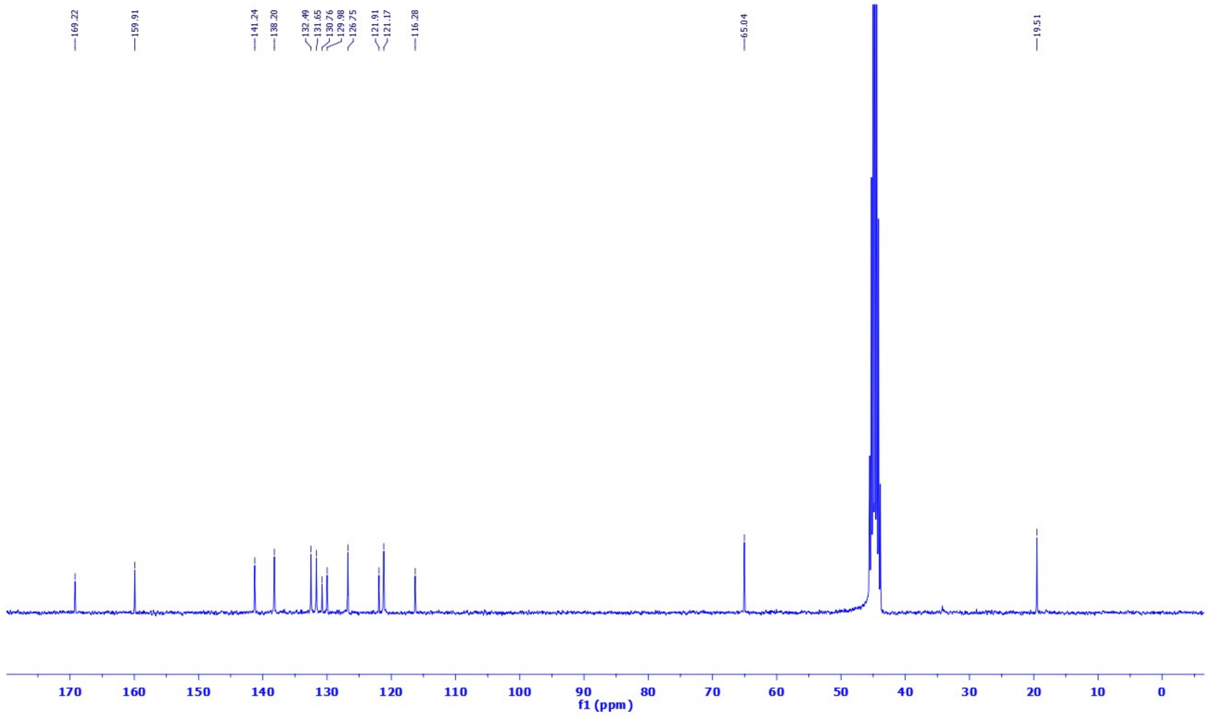

^{1}H-NMR of 4ba:

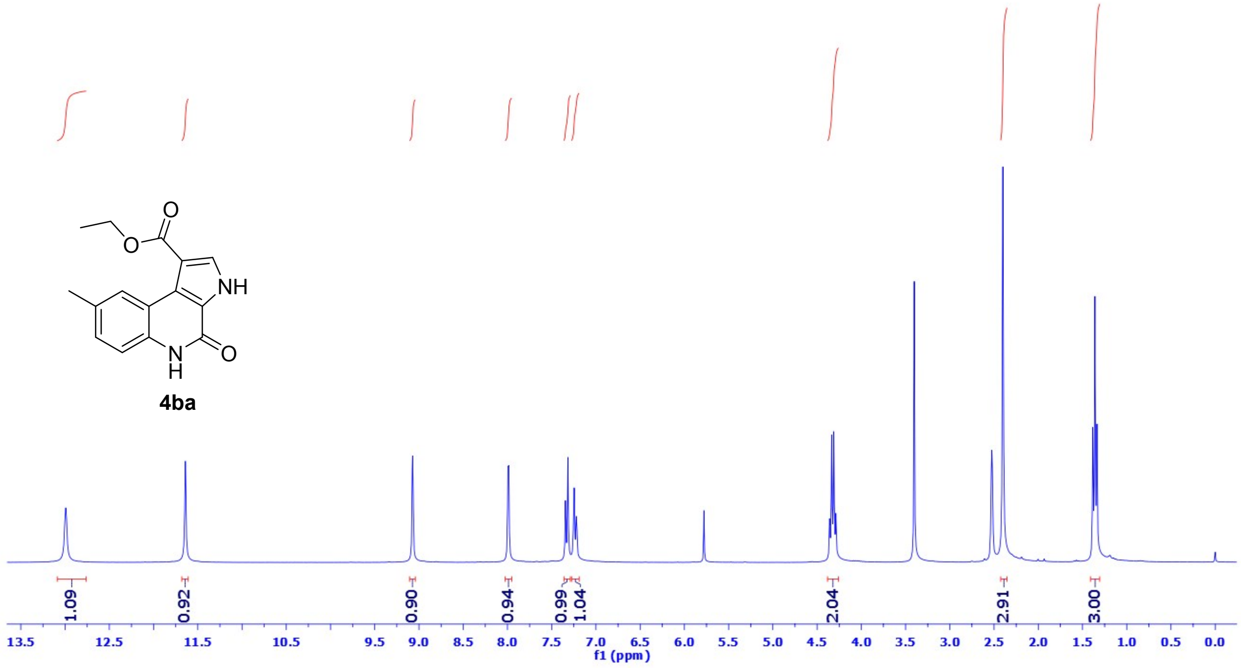

^{13}C-NMR of 4ba:

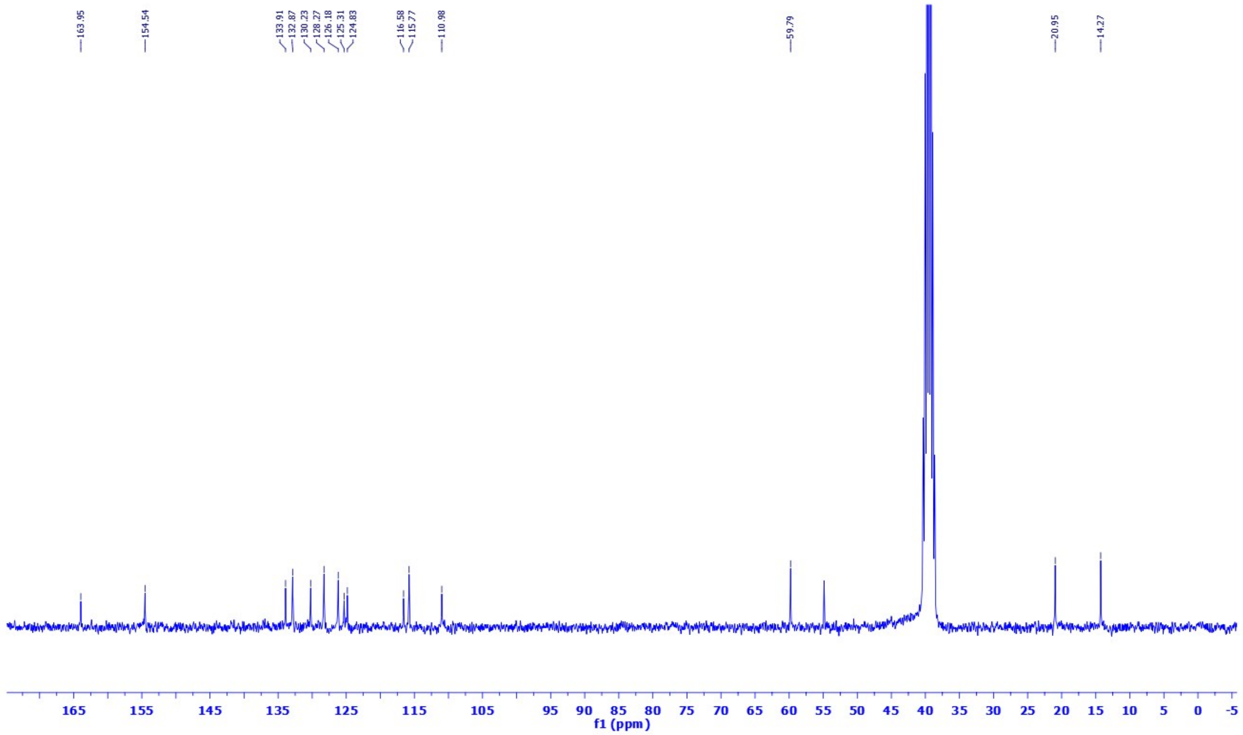

^{1}H-NMR of 4ca:

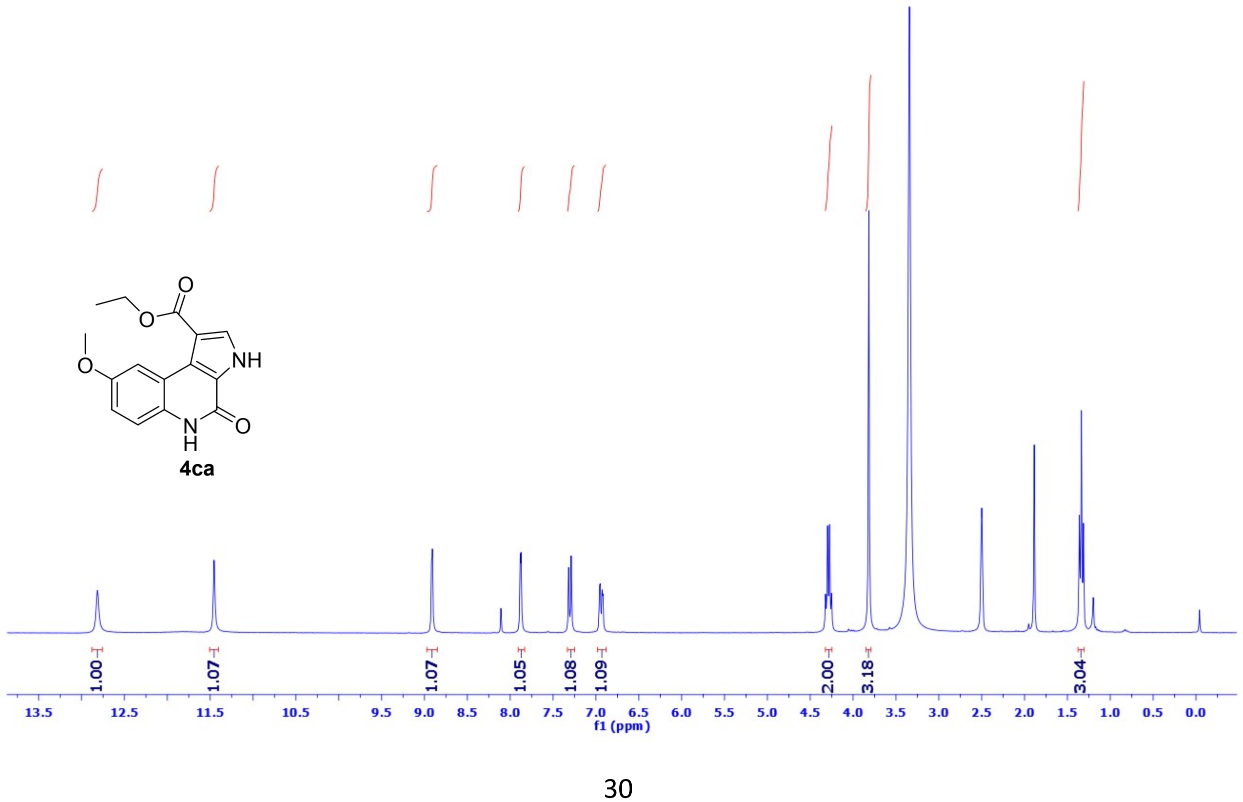

^{13}C-NMR of 4ca:

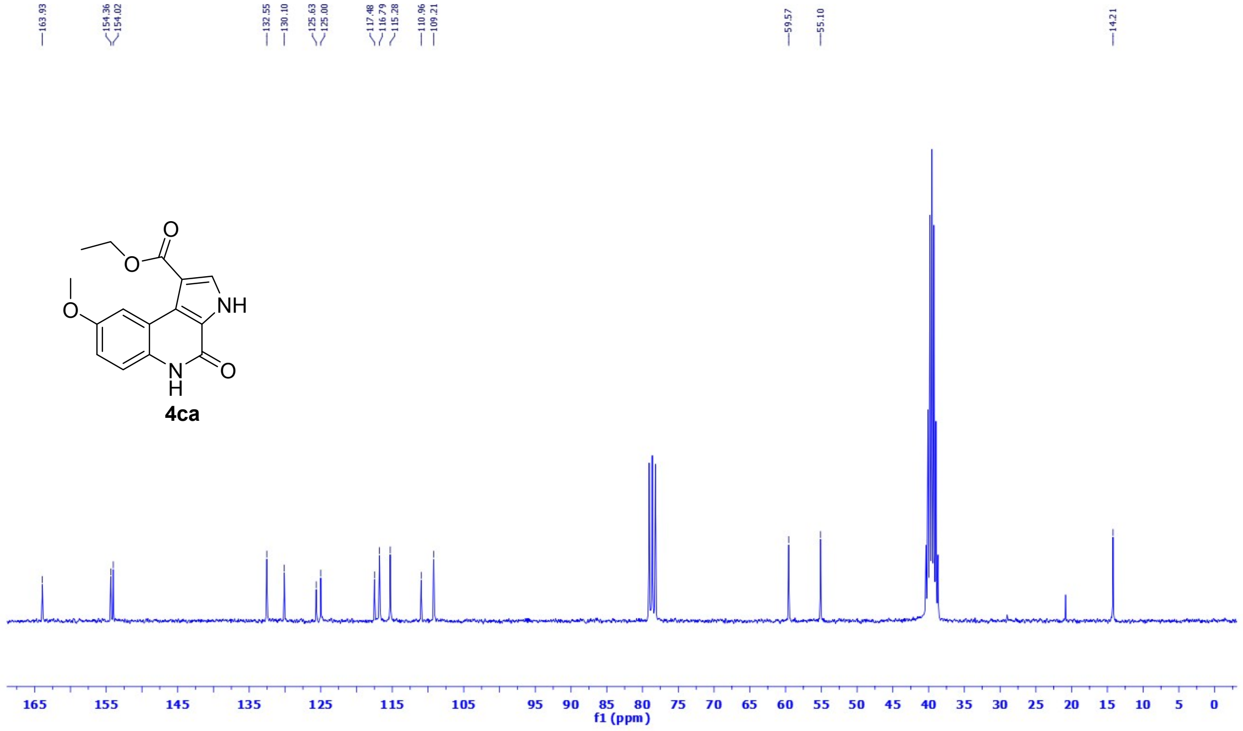

^{1}H-NMR of 4da:

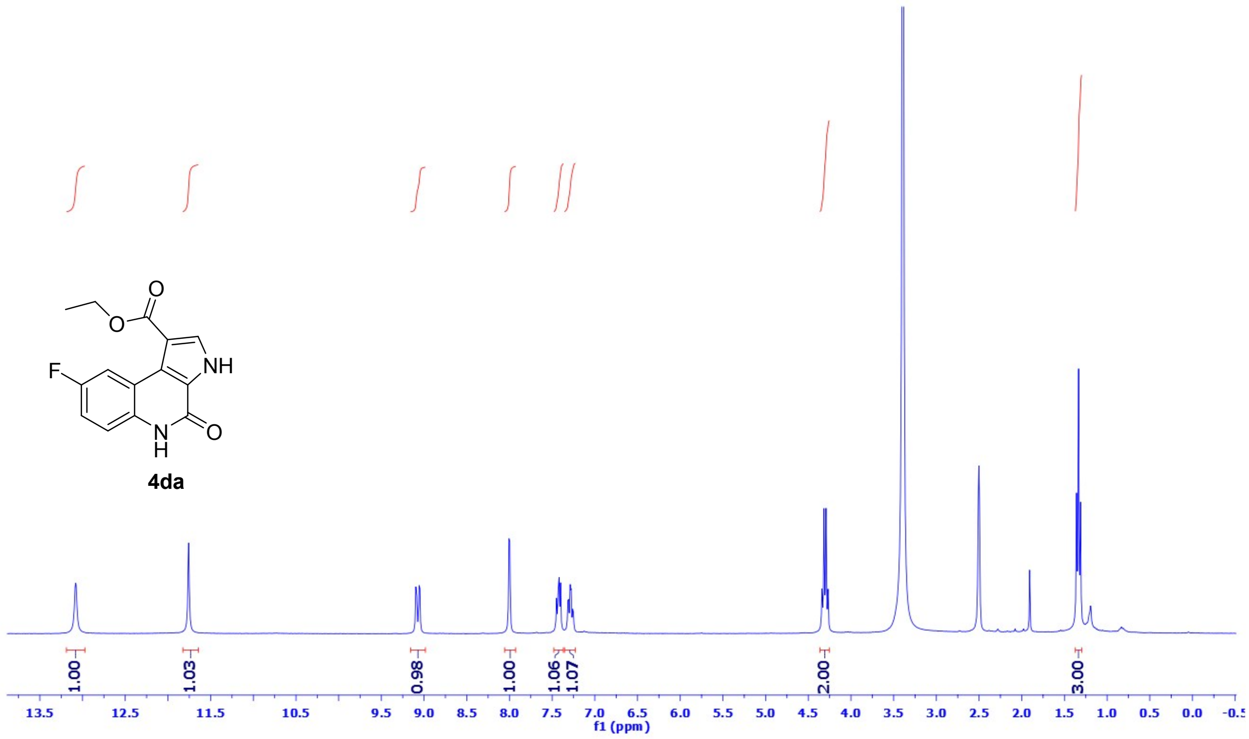

^{13}C-NMR of 4da:

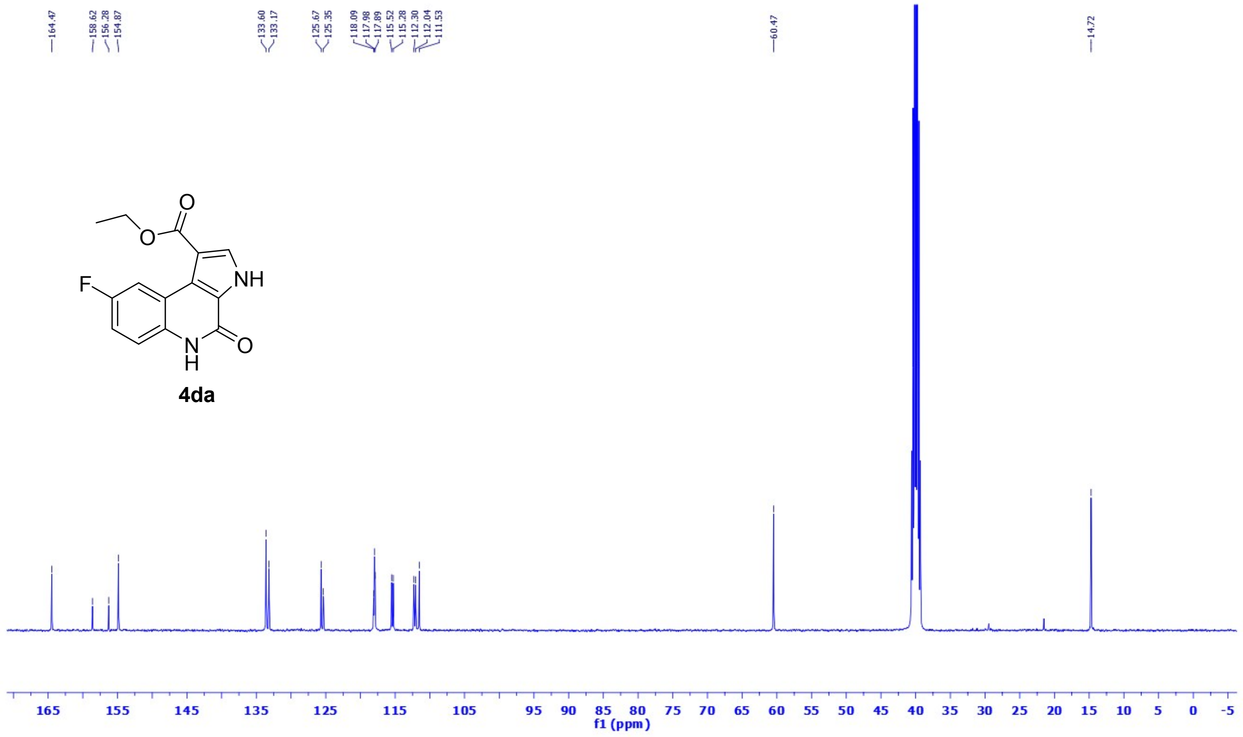

^{1}H-NMR of 4ea:

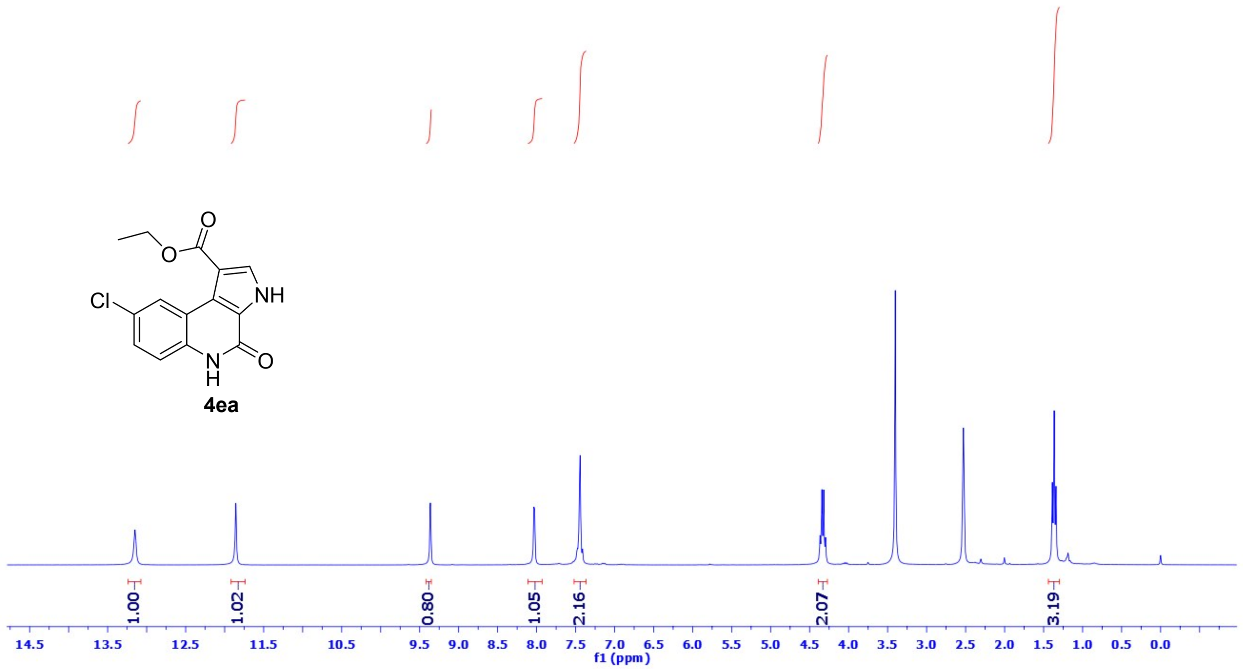

^{13}C-NMR of 4ea:

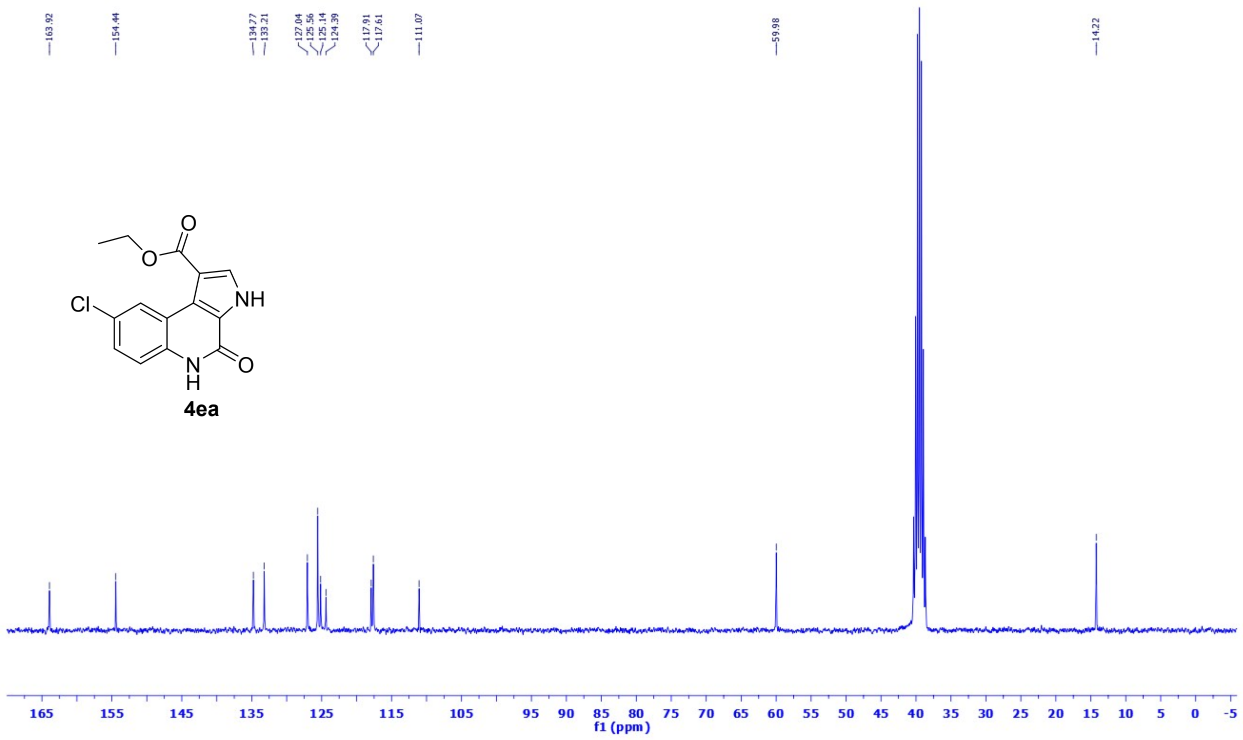

^{1}H-NMR of 4fa:

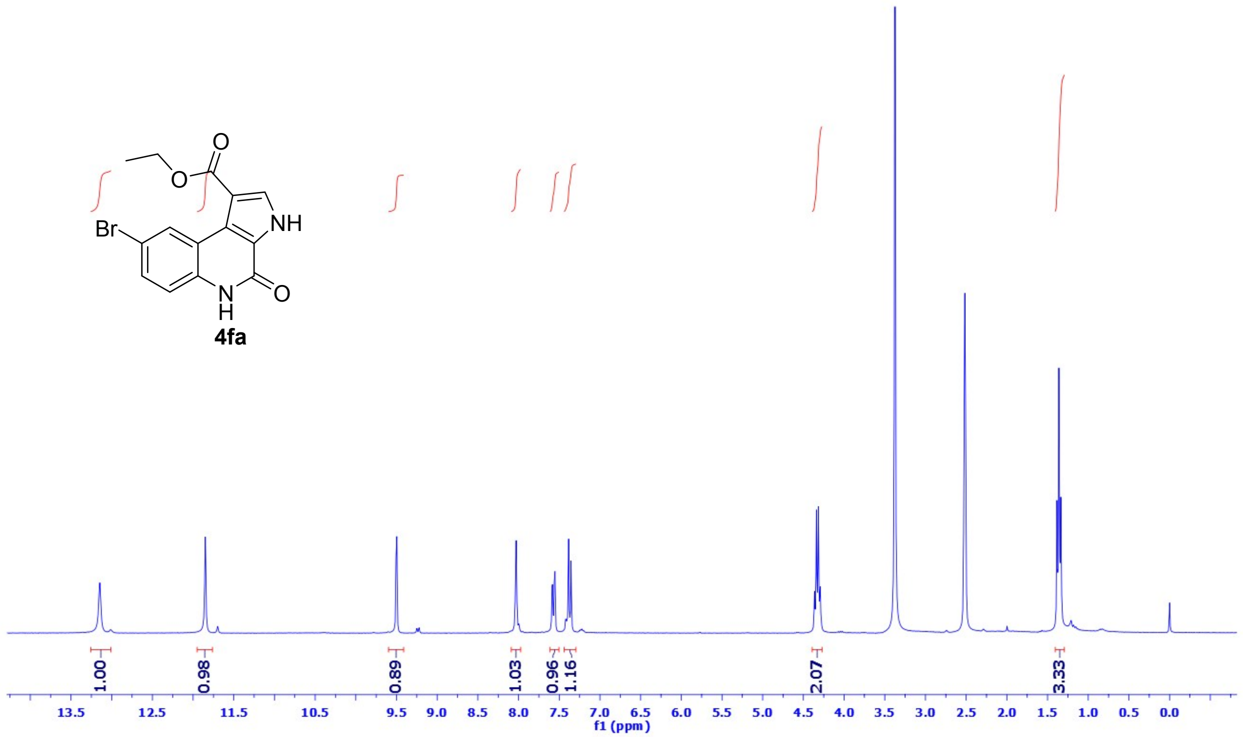

^{13}C-NMR of 4fa:

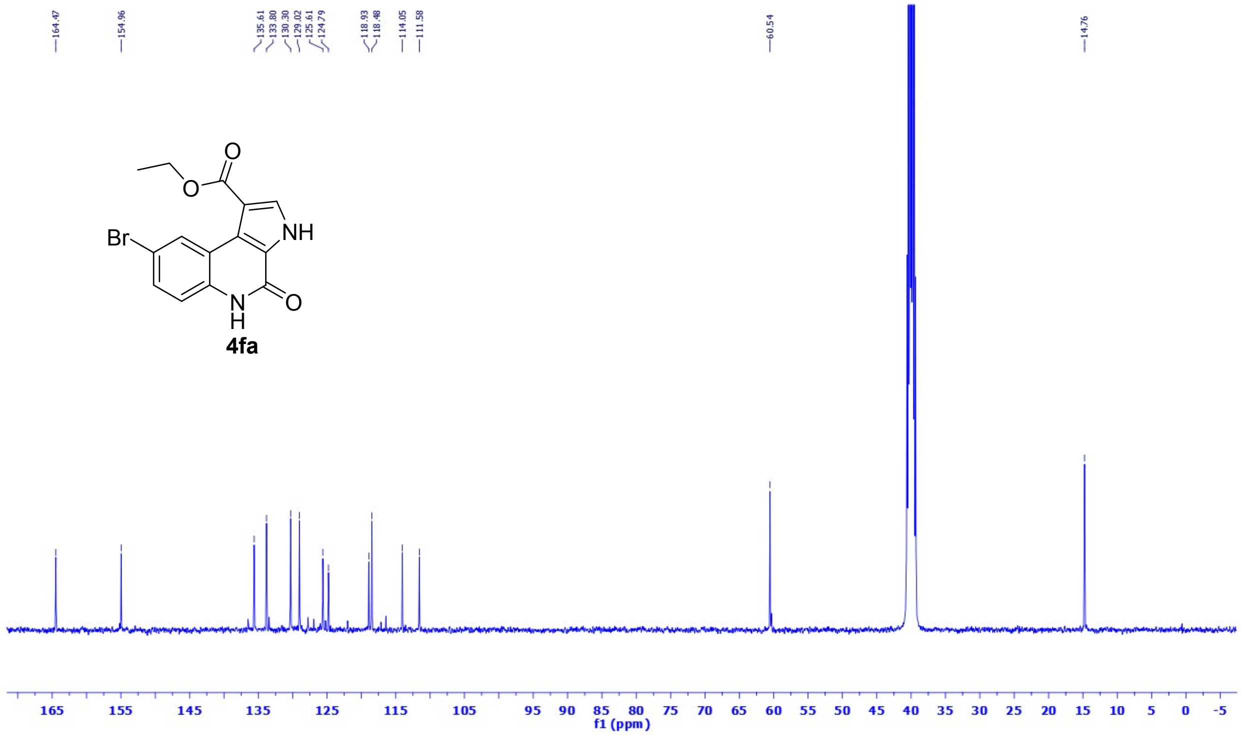

^{1}H-NMR of 4ga:

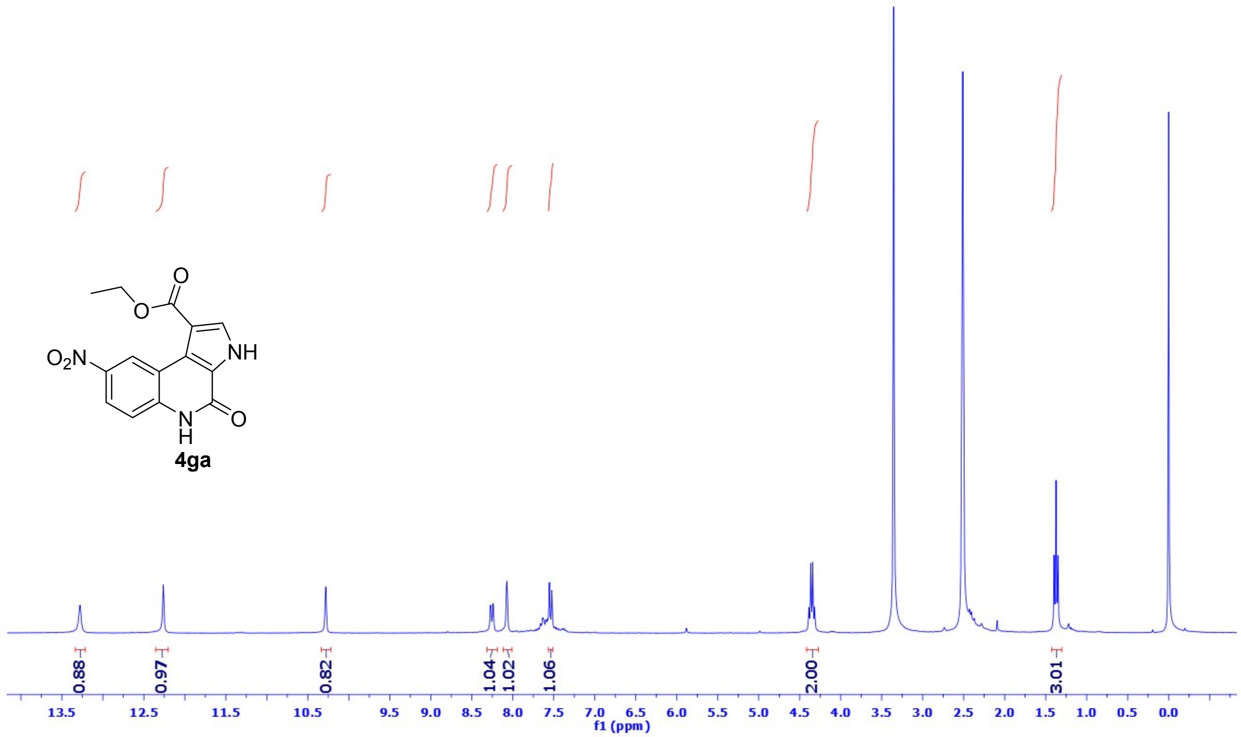

^{13}C-NMR of 4ga:

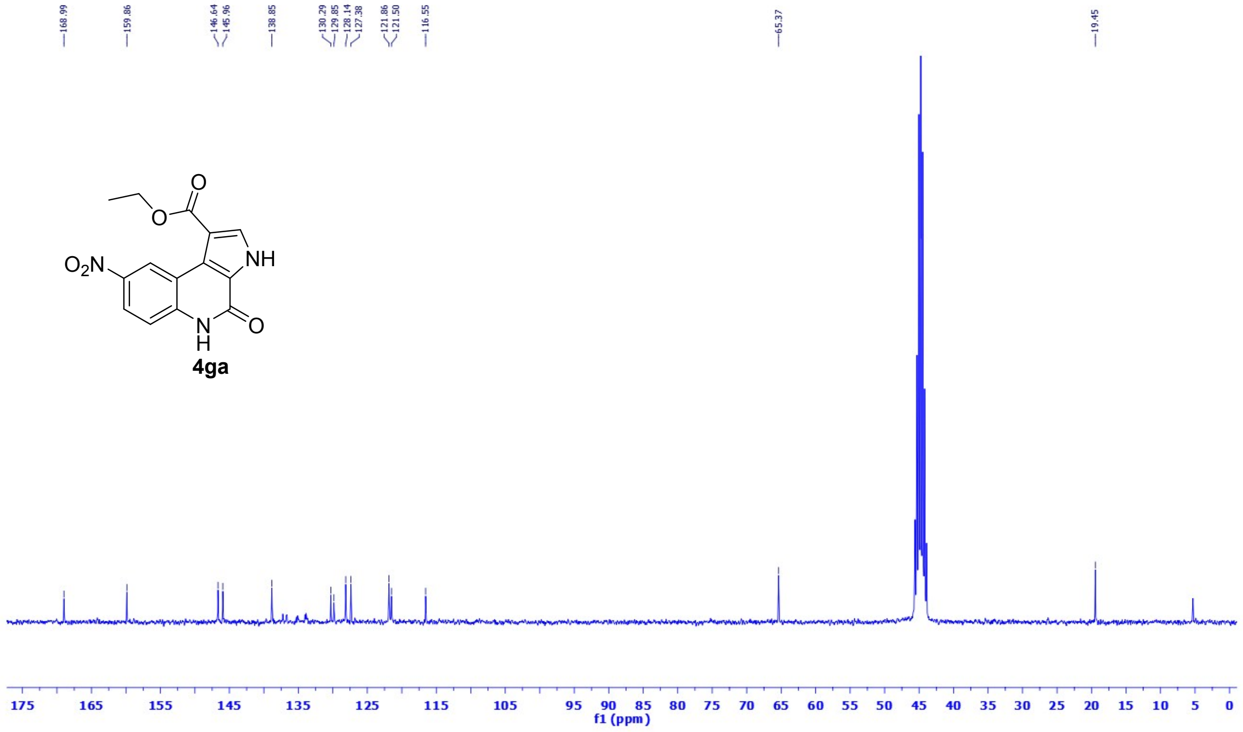

^{1}H-NMR of 4ha:

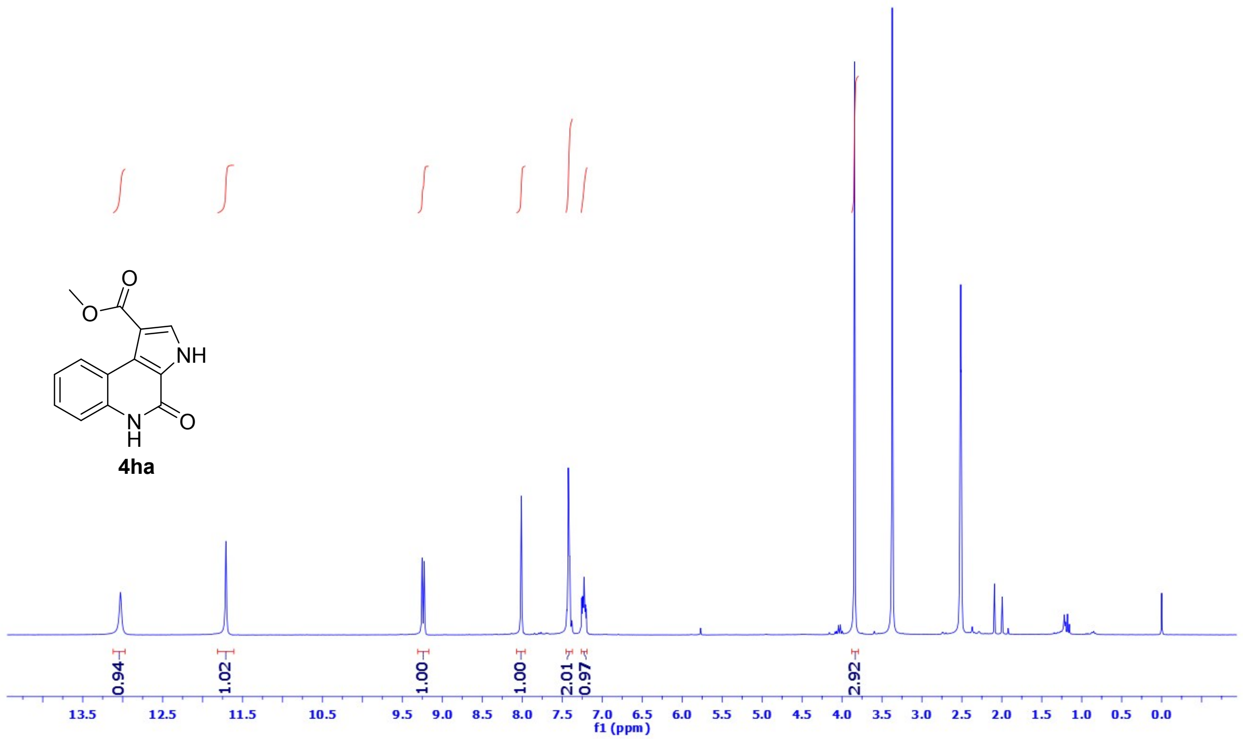

^{13}C-NMR of 4ha:

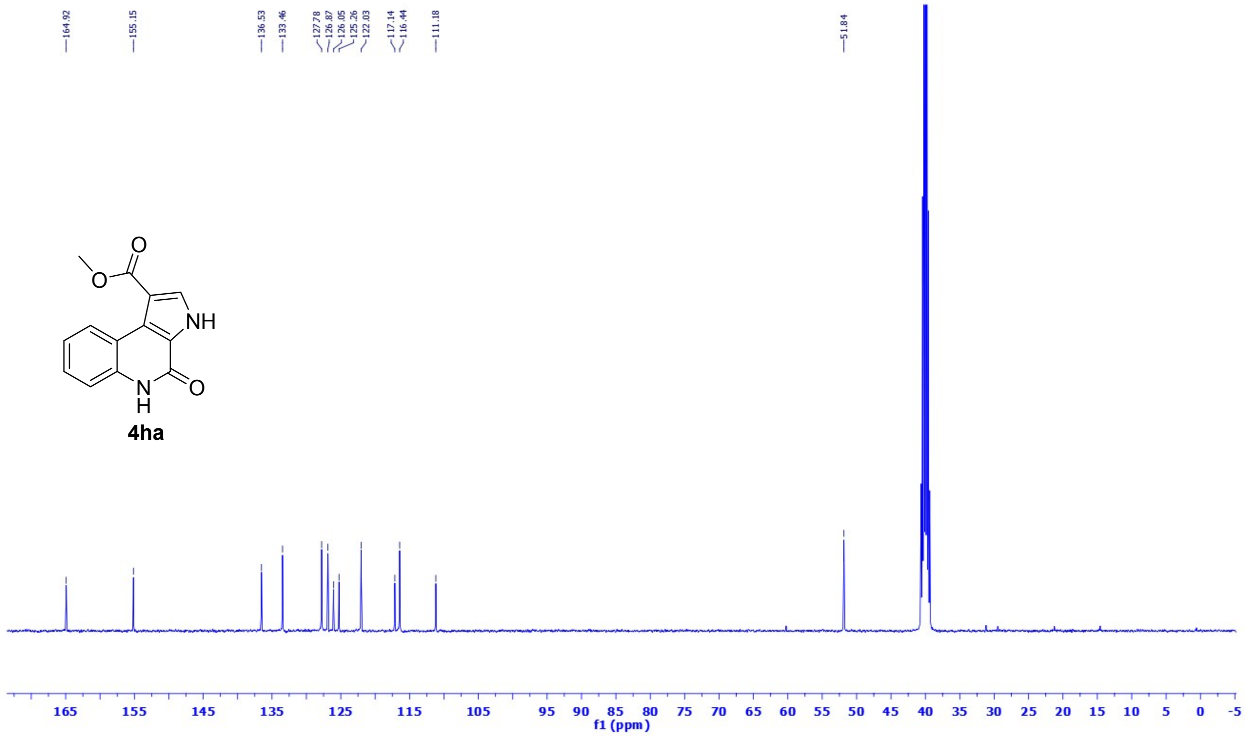

^{1}H-NMR of 4ia:

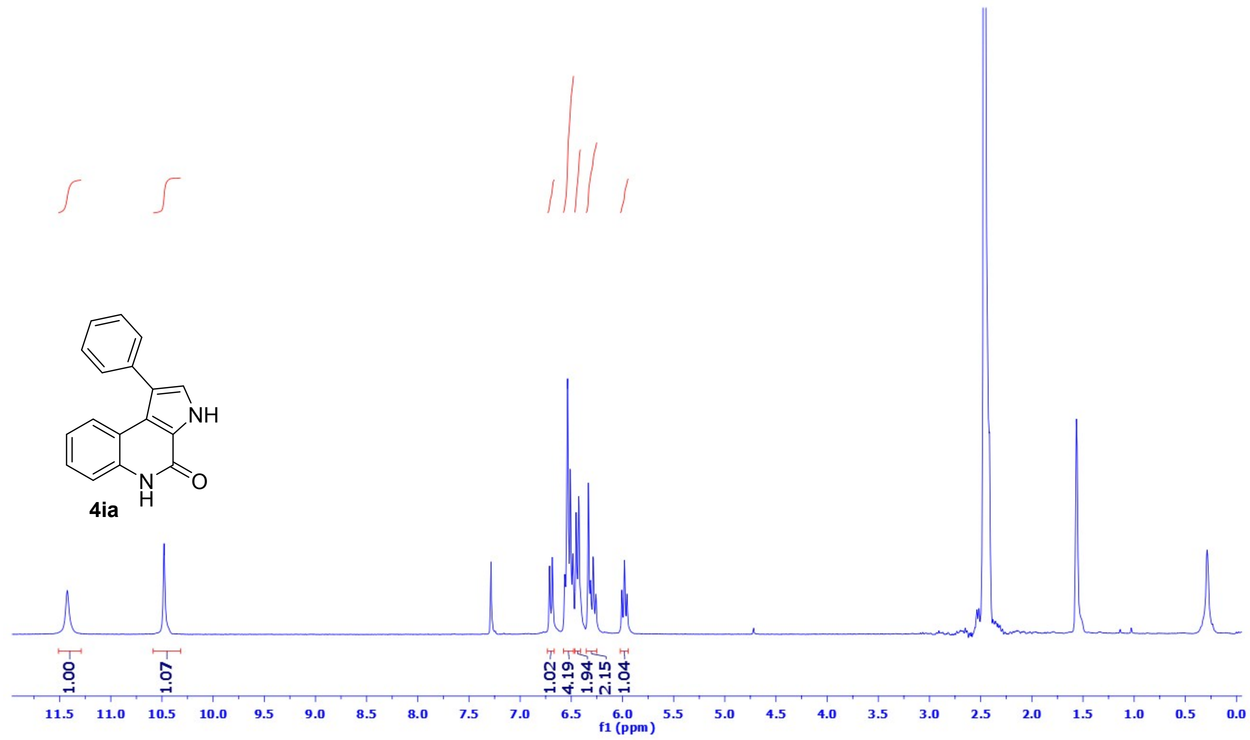

^{13}C-NMR of 4ia:

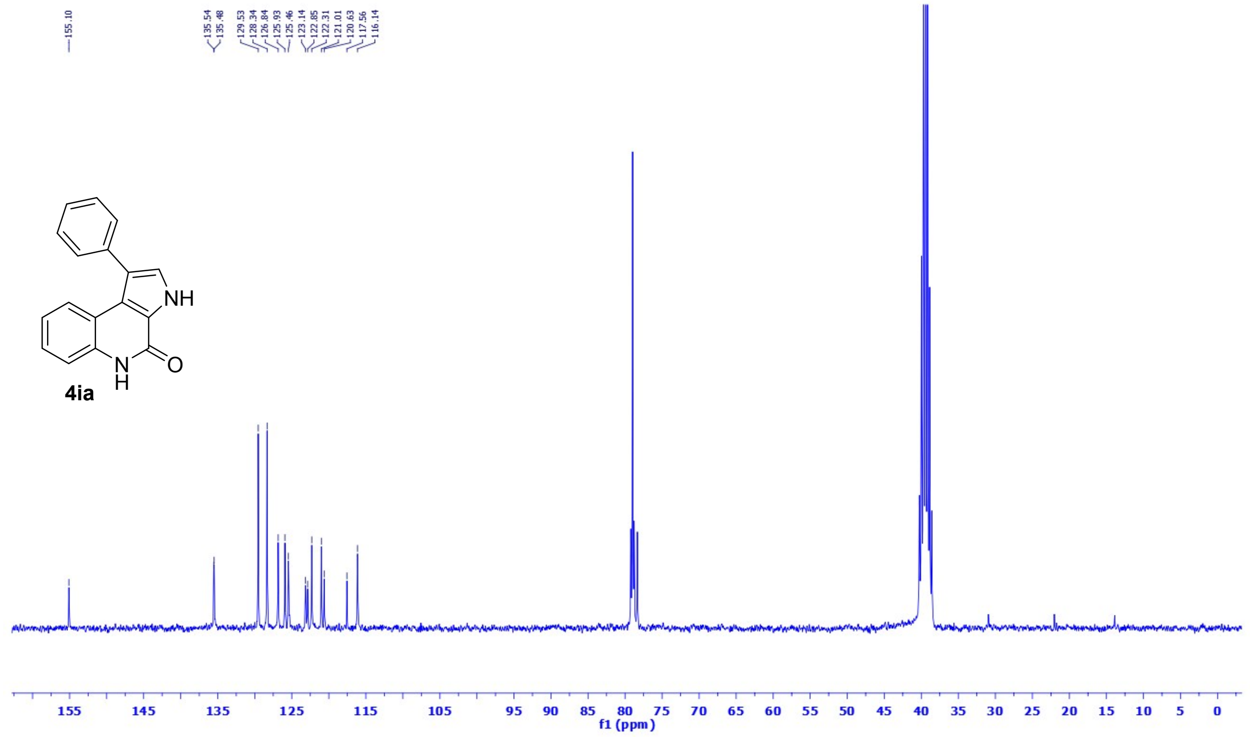

^{1}H-NMR of 4ja:

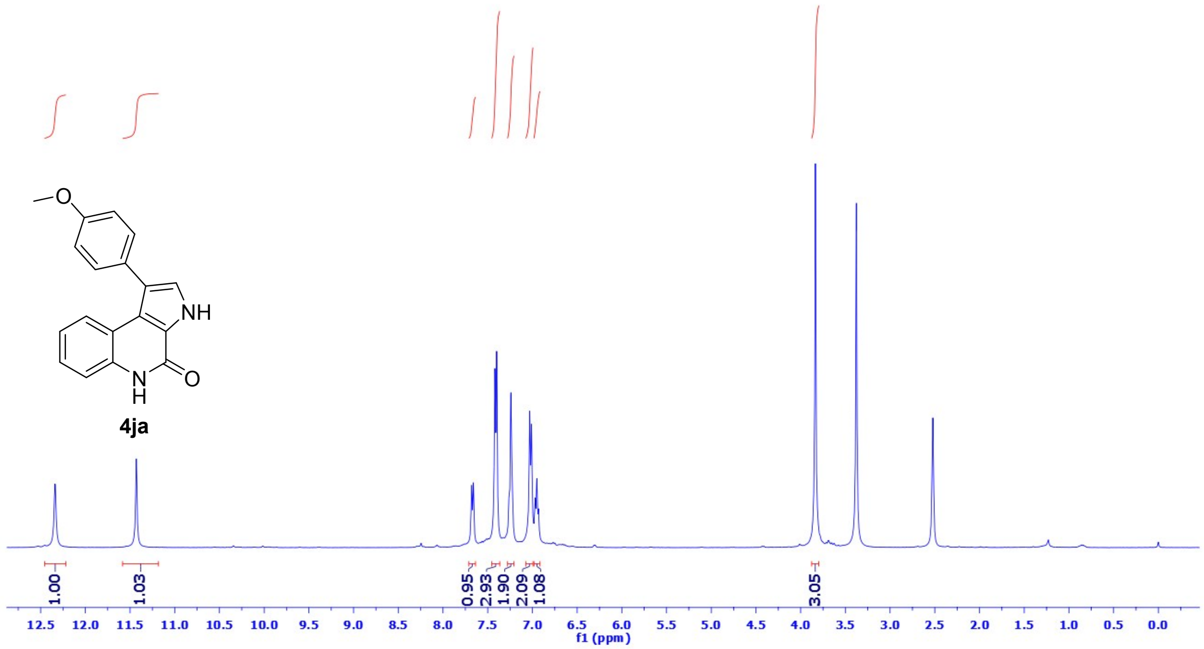

^{13}C-NMR of 4ja:

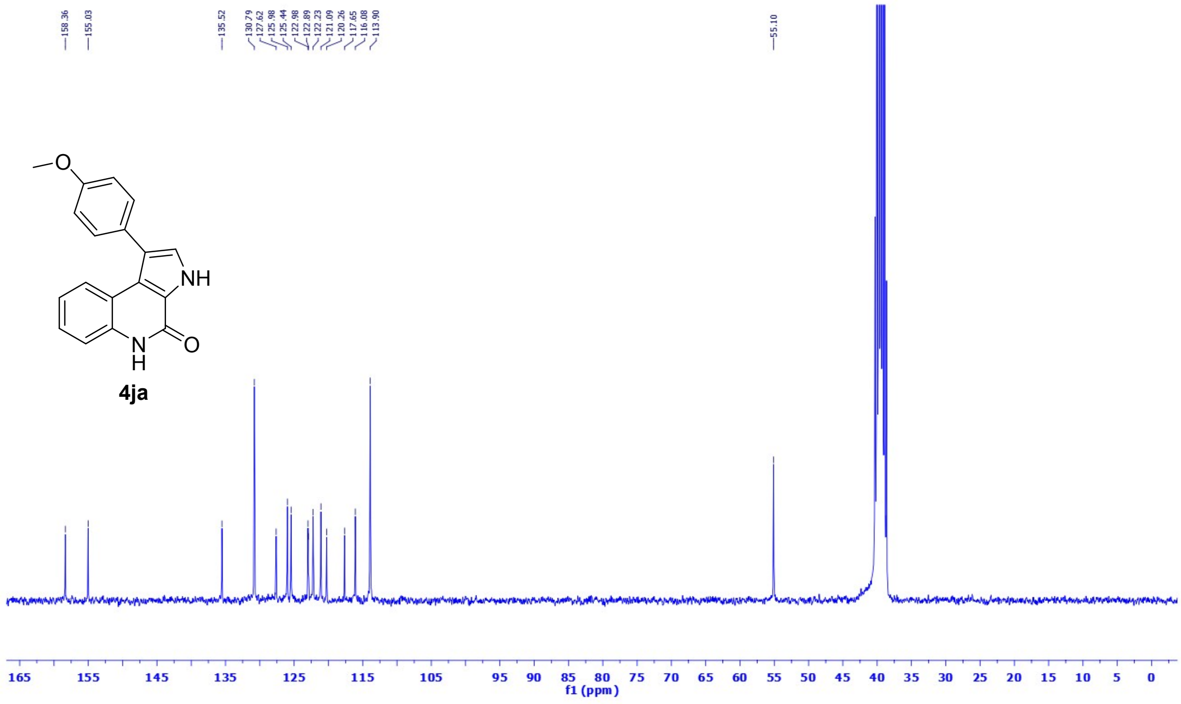

^{1}H-NMR of 4ka:

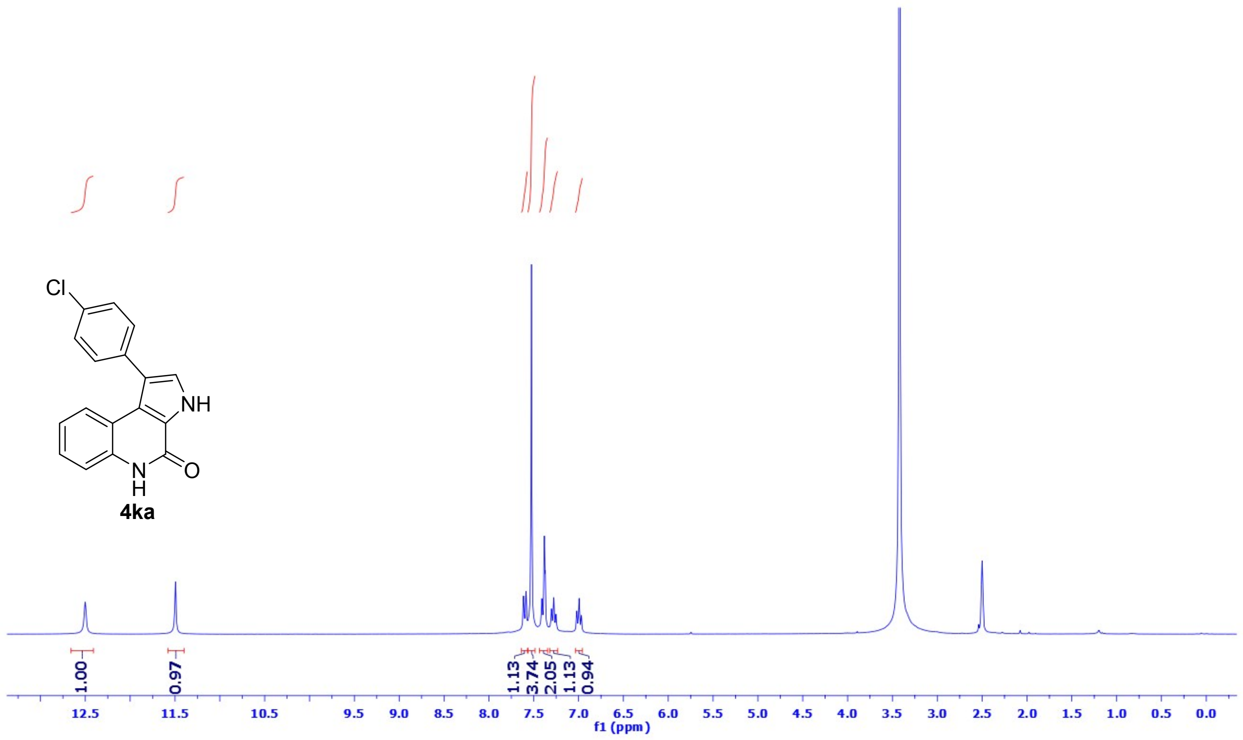

^{13}C-NMR of 4ka:

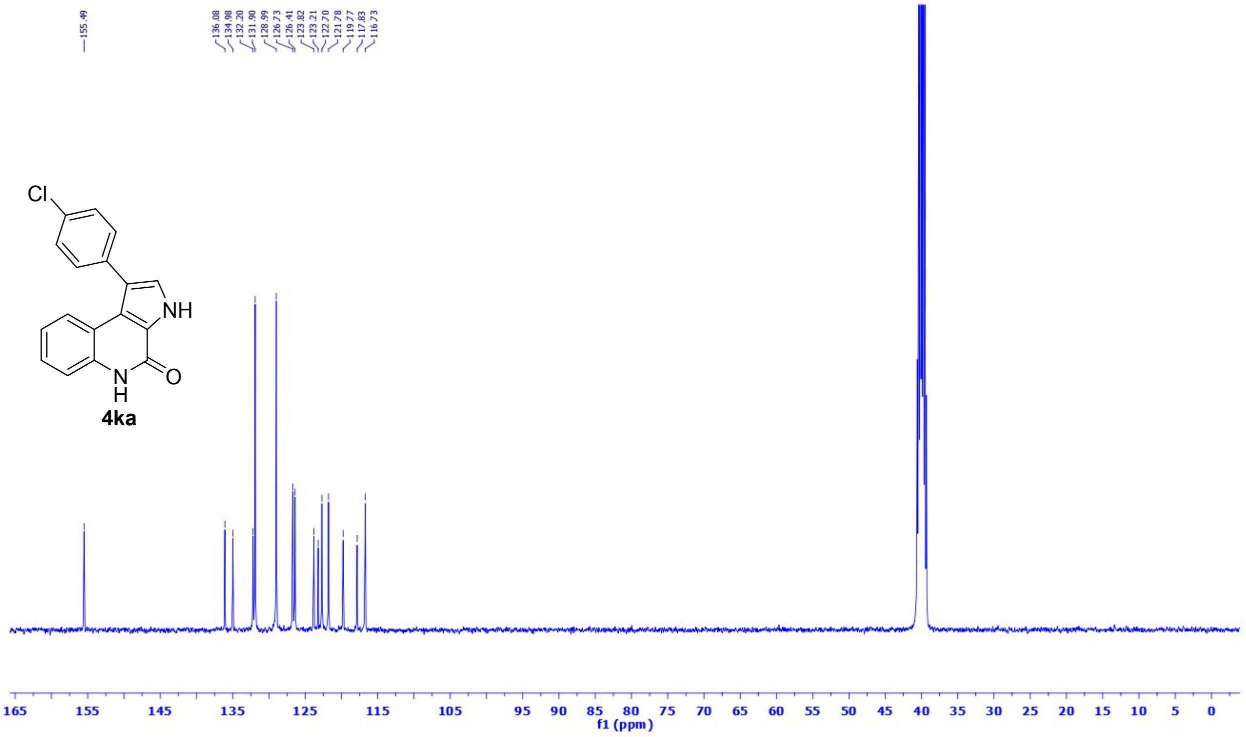

^{1}H-NMR of 4la:

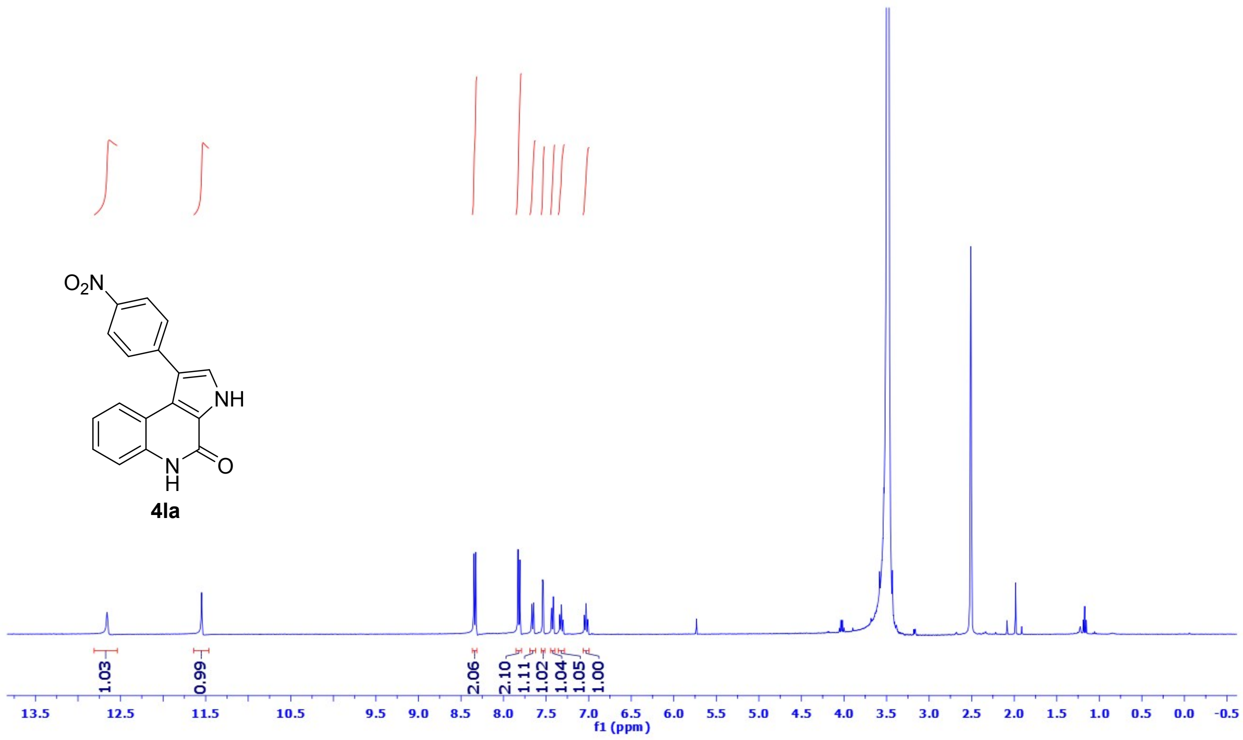

^{13}C-NMR of 4la:

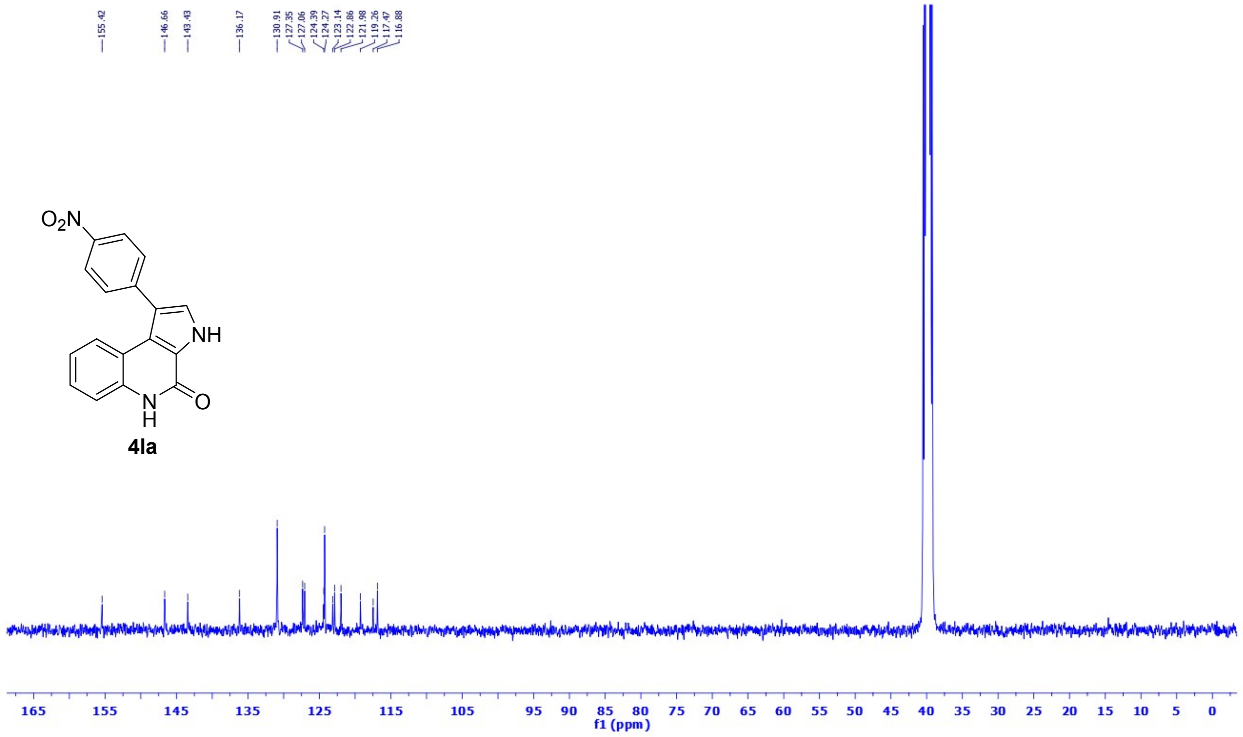

^{1}H-NMR of 4ma:

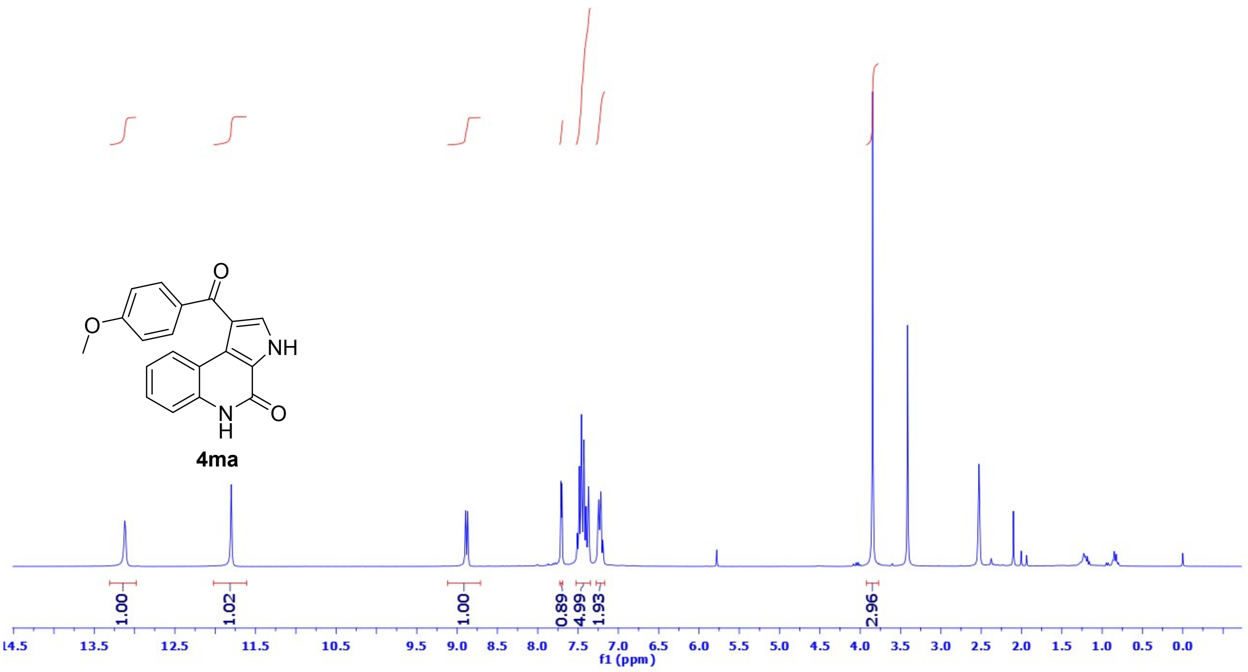

^{13}C-NMR of 4ma:

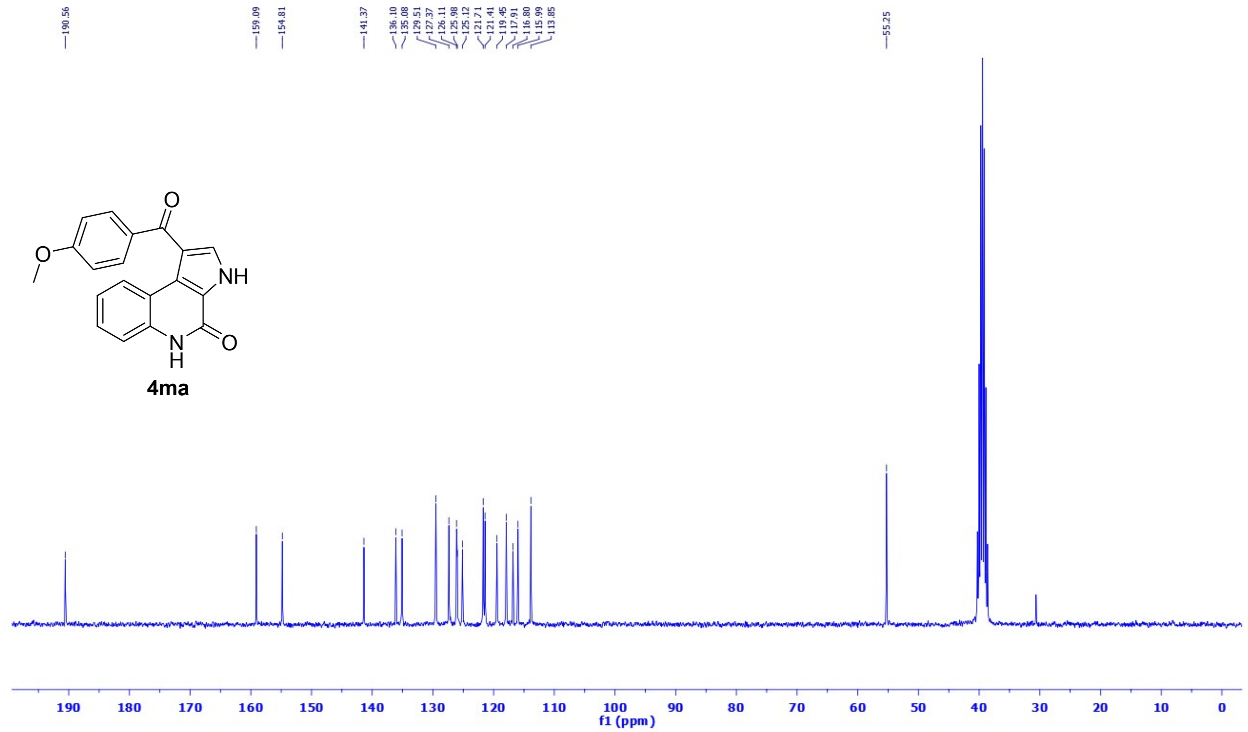

^{1}H-NMR of 4na:

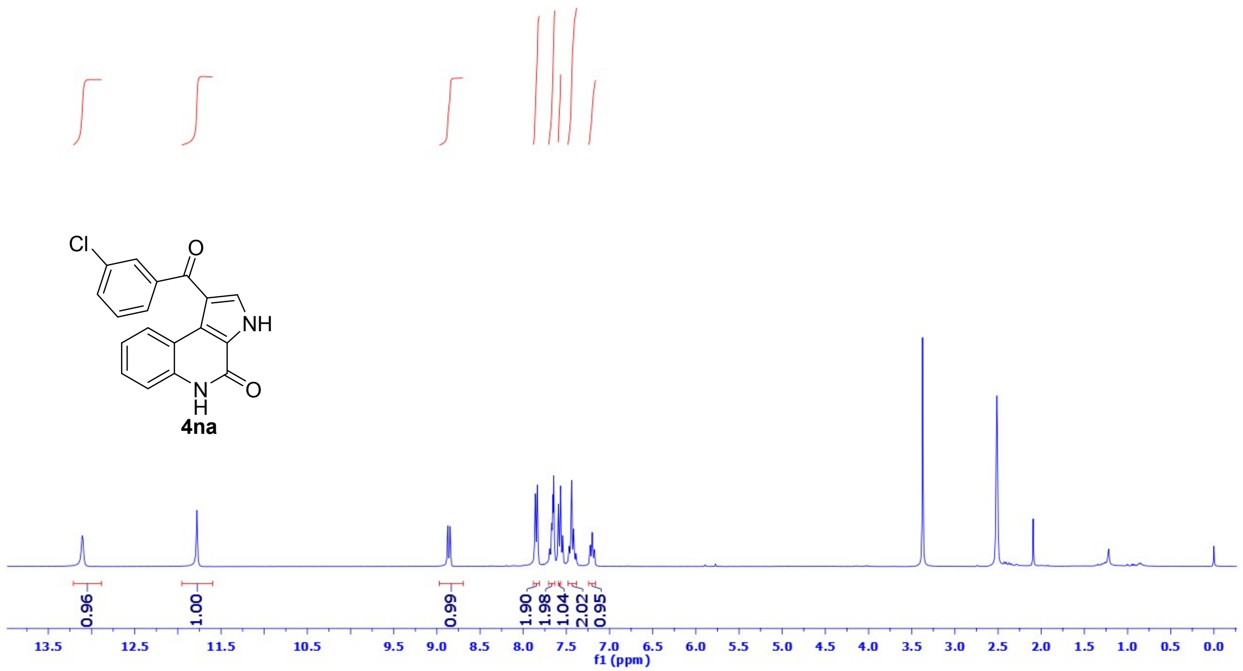

^{13}C-NMR of 4na:

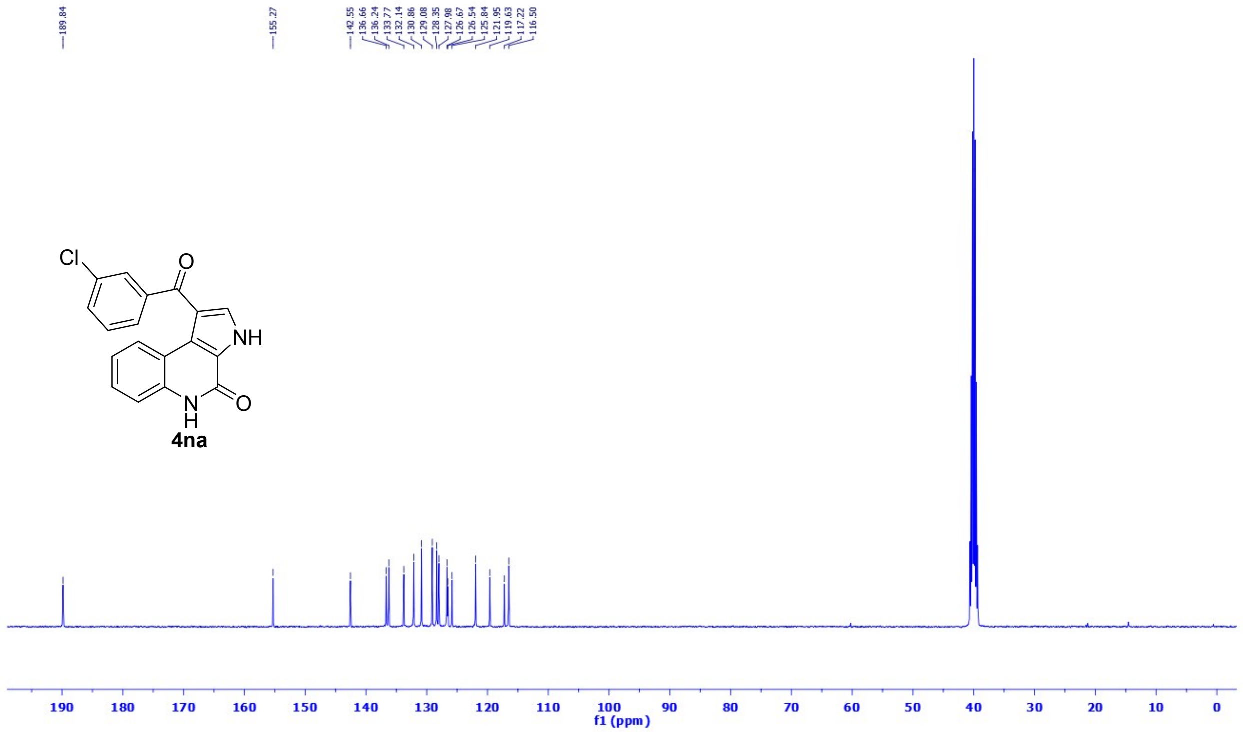

^{1}H-NMR of 4oa:

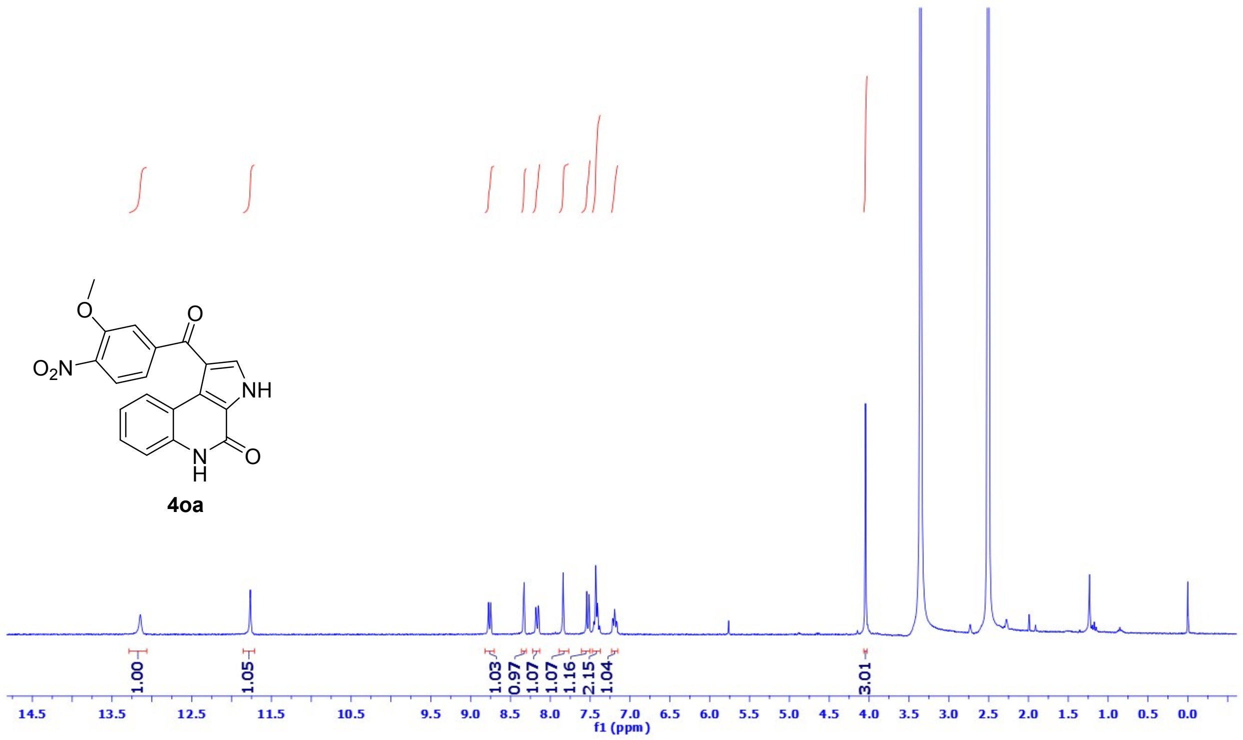

^{13}C-NMR of 4oa:

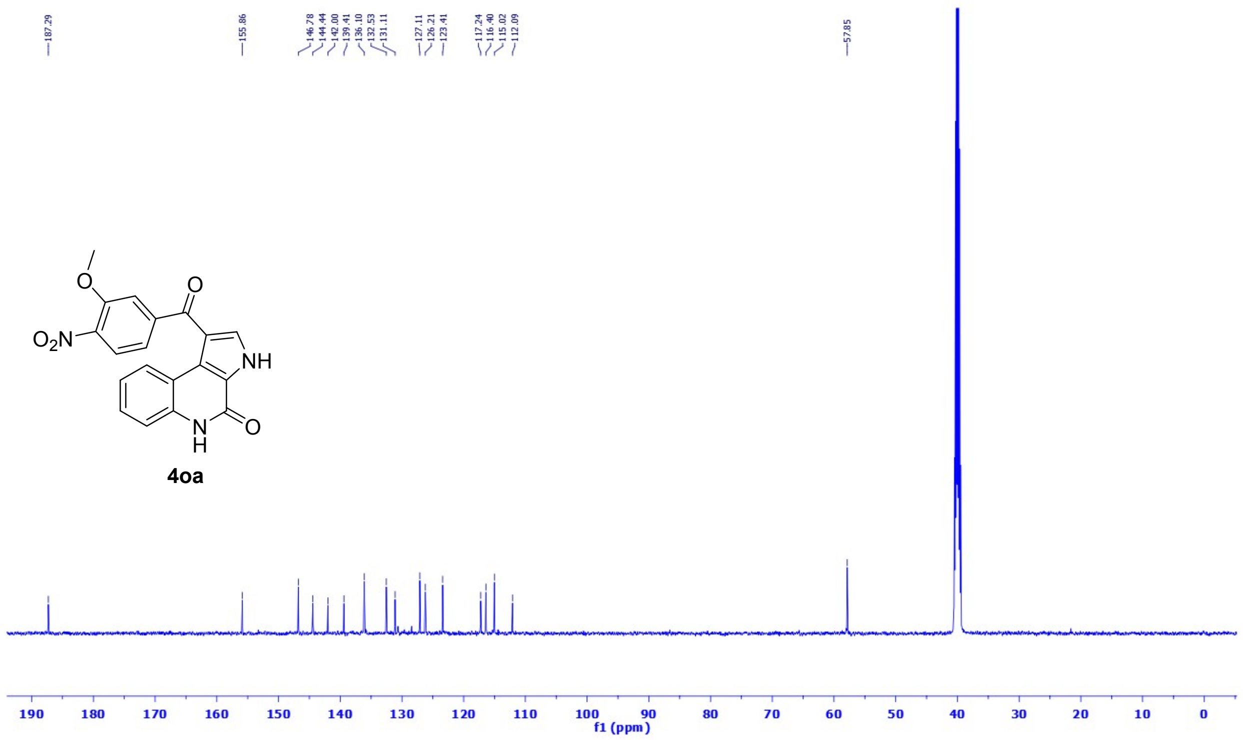

^{1}H-NMR of 4pa:

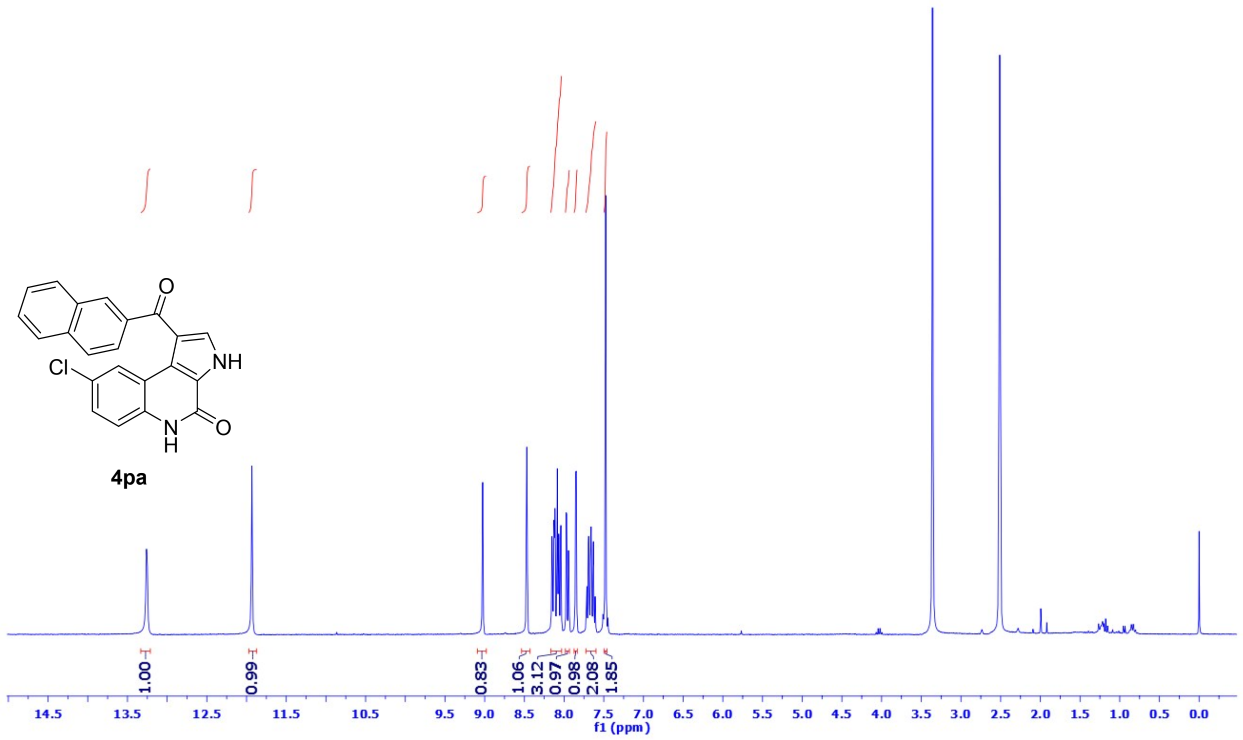

^{13}C-NMR of 4pa:

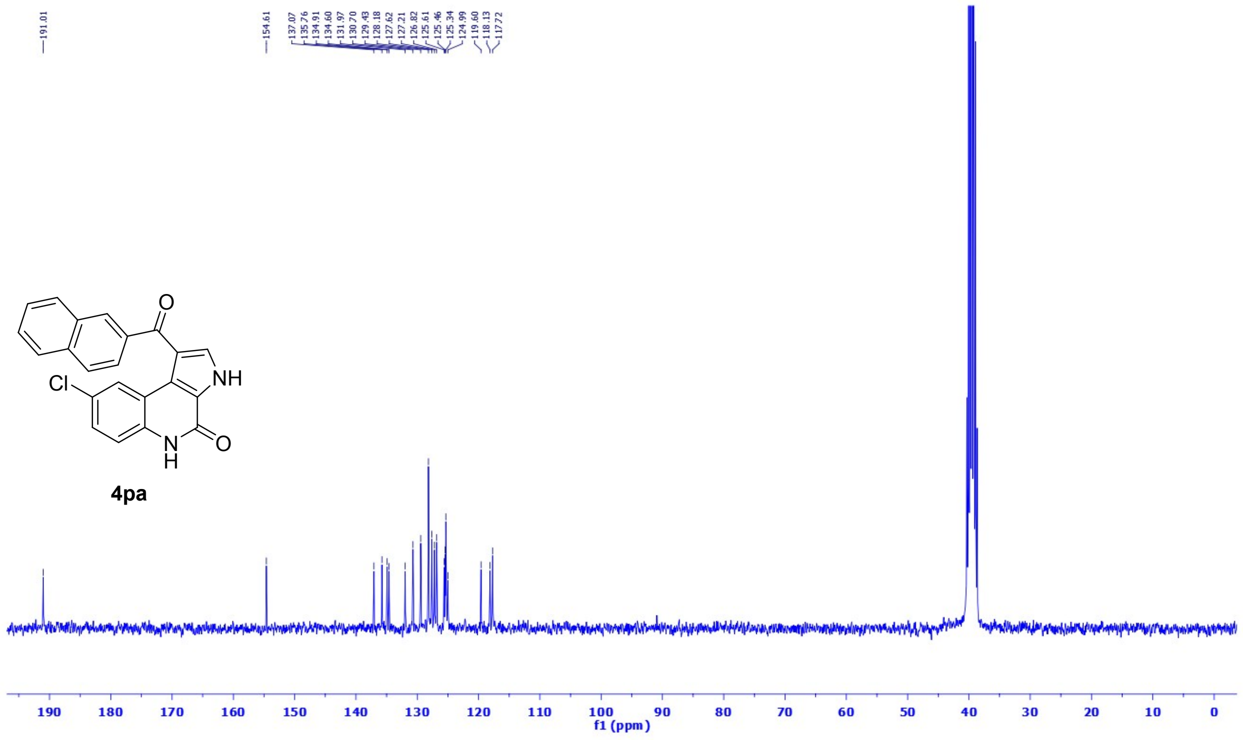

^{1}H-NMR of 4qa:

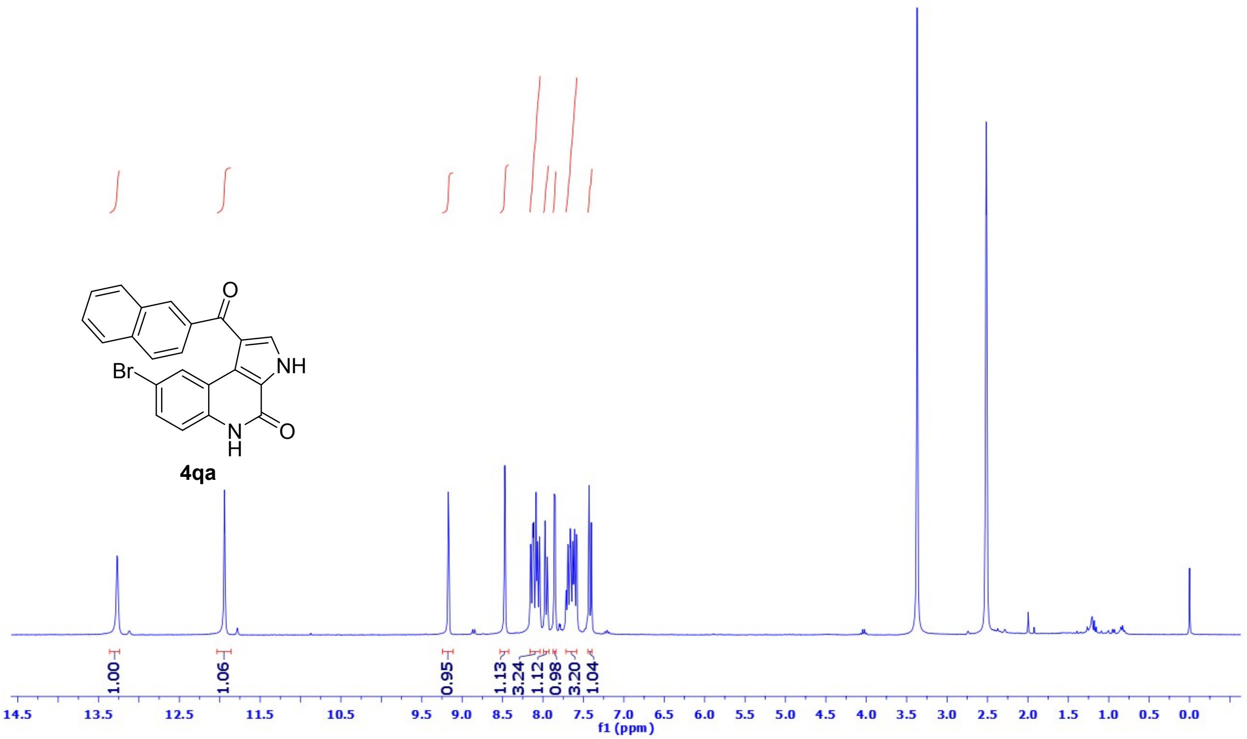

^{13}C-NMR of 4qa:

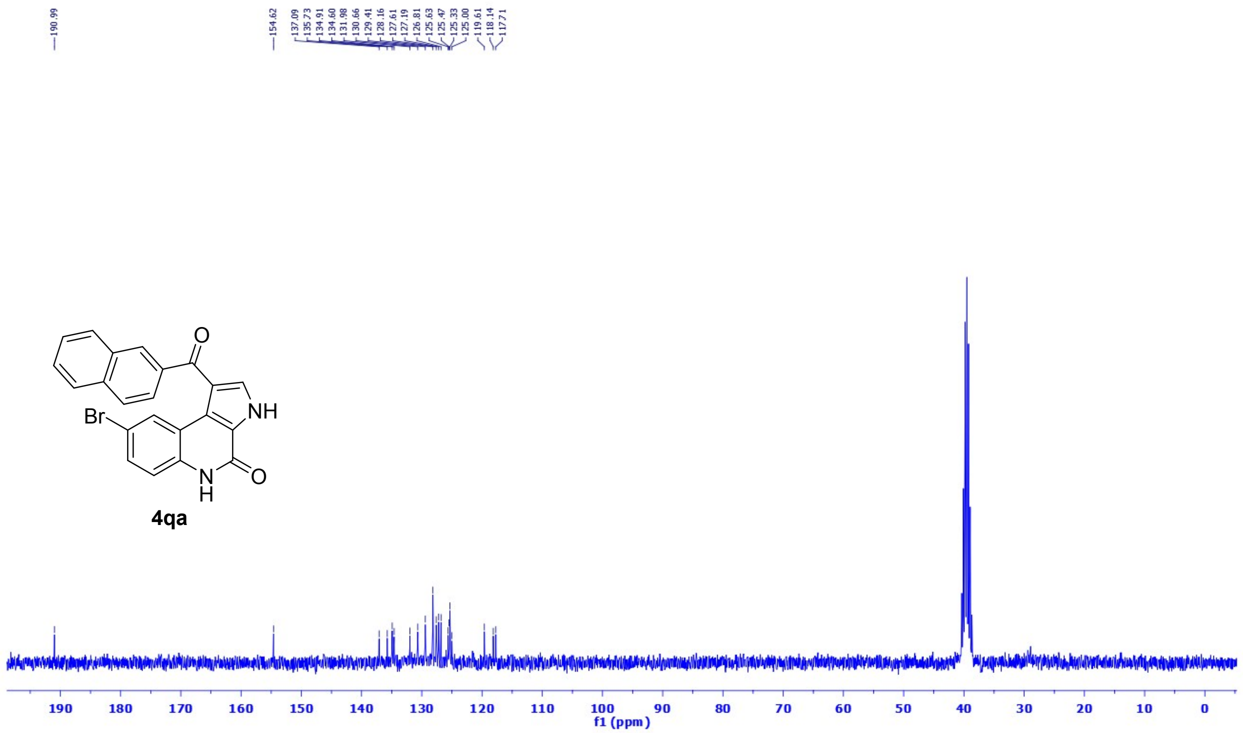

^{1}H-NMR of 5:

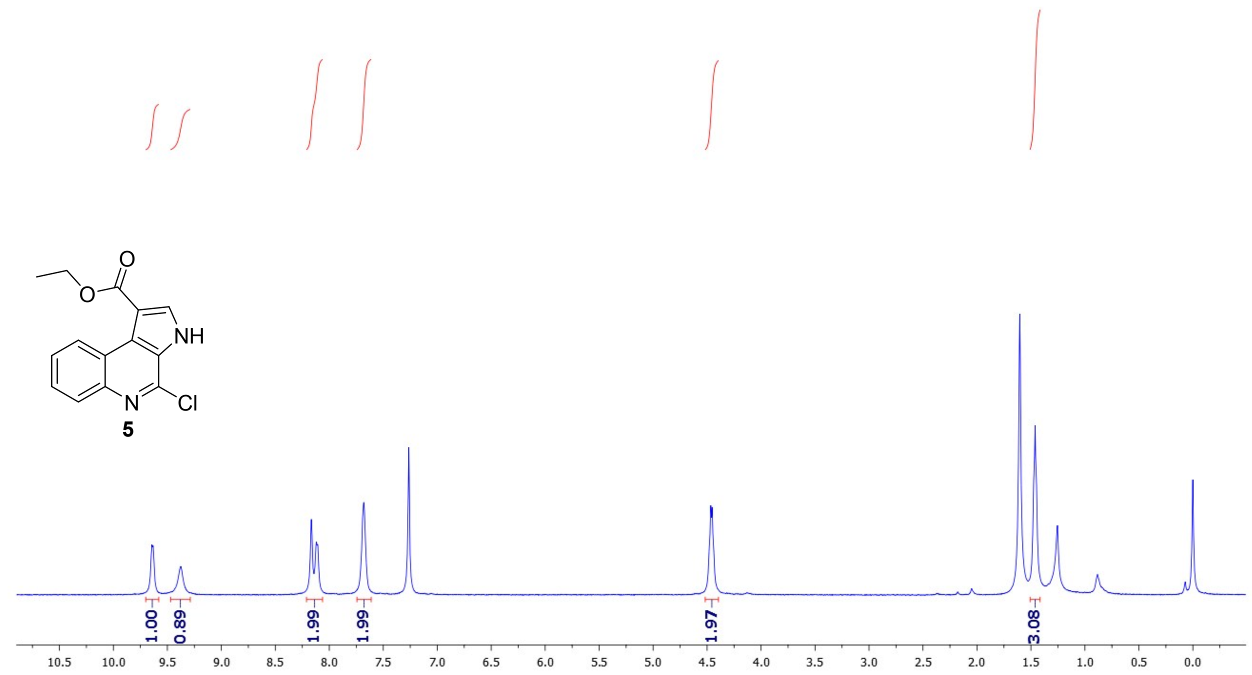

^{13}C-NMR of 5:

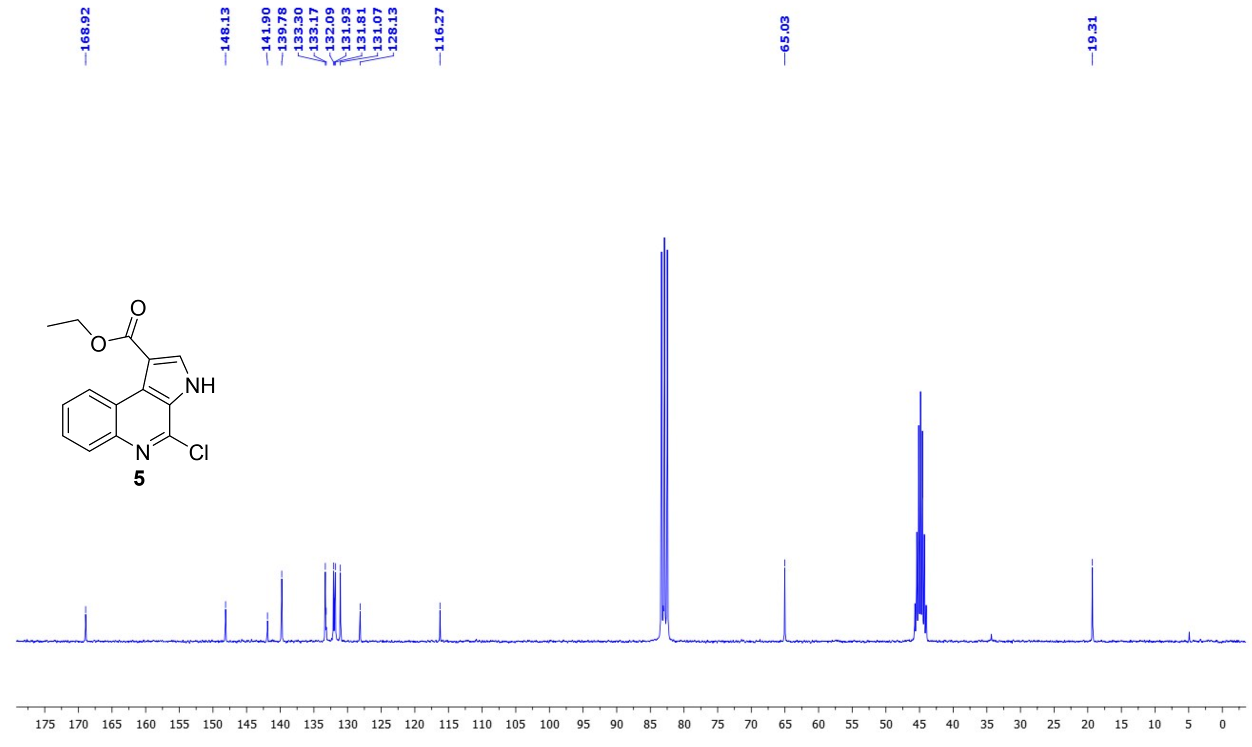

^{1}H-NMR of 6:

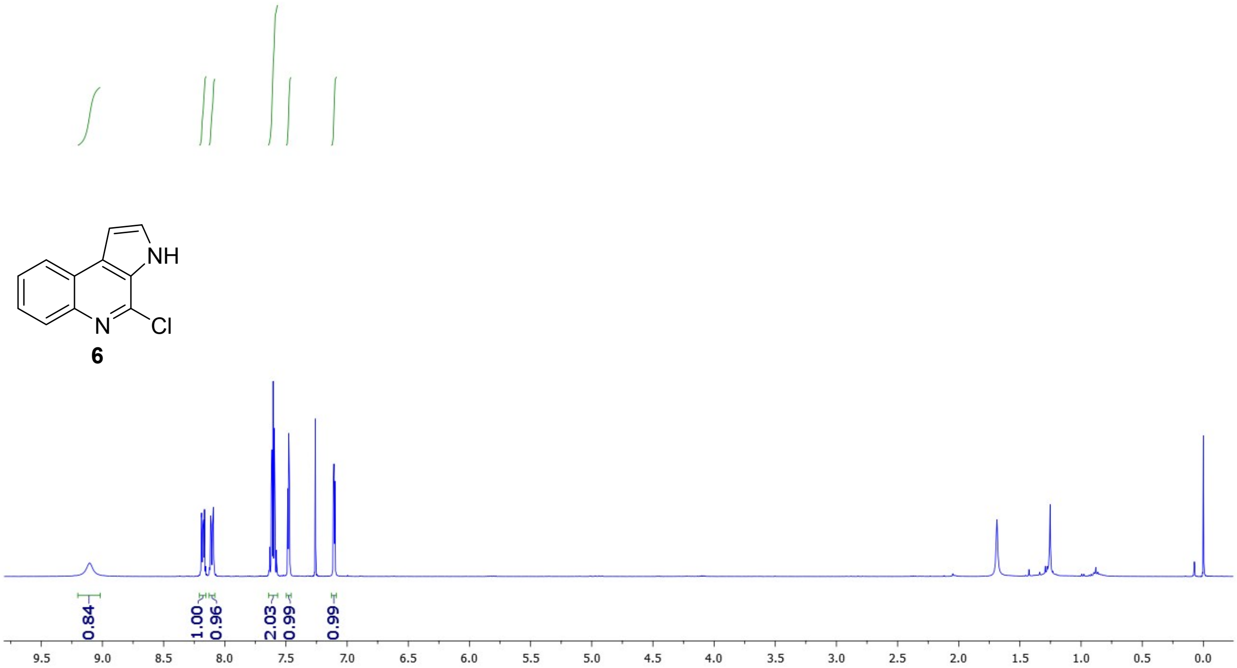

^{13}C-NMR of 6:

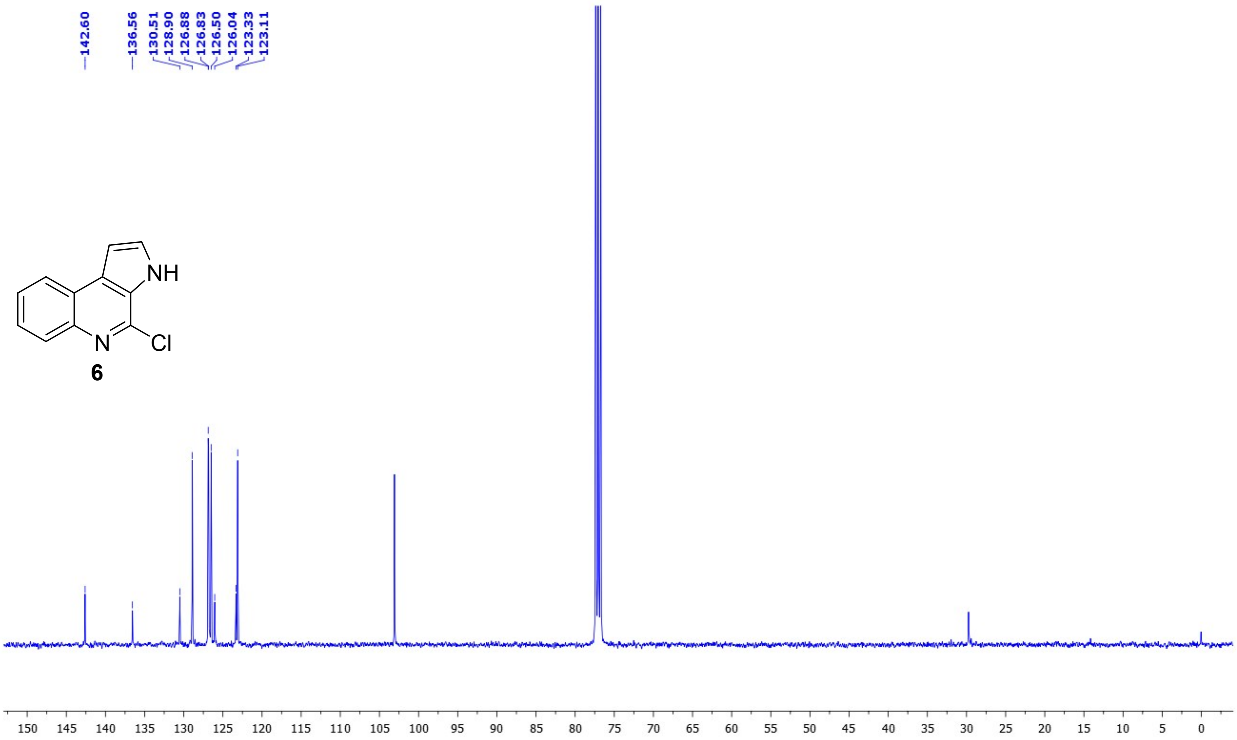

^{1}H-NMR of 7:

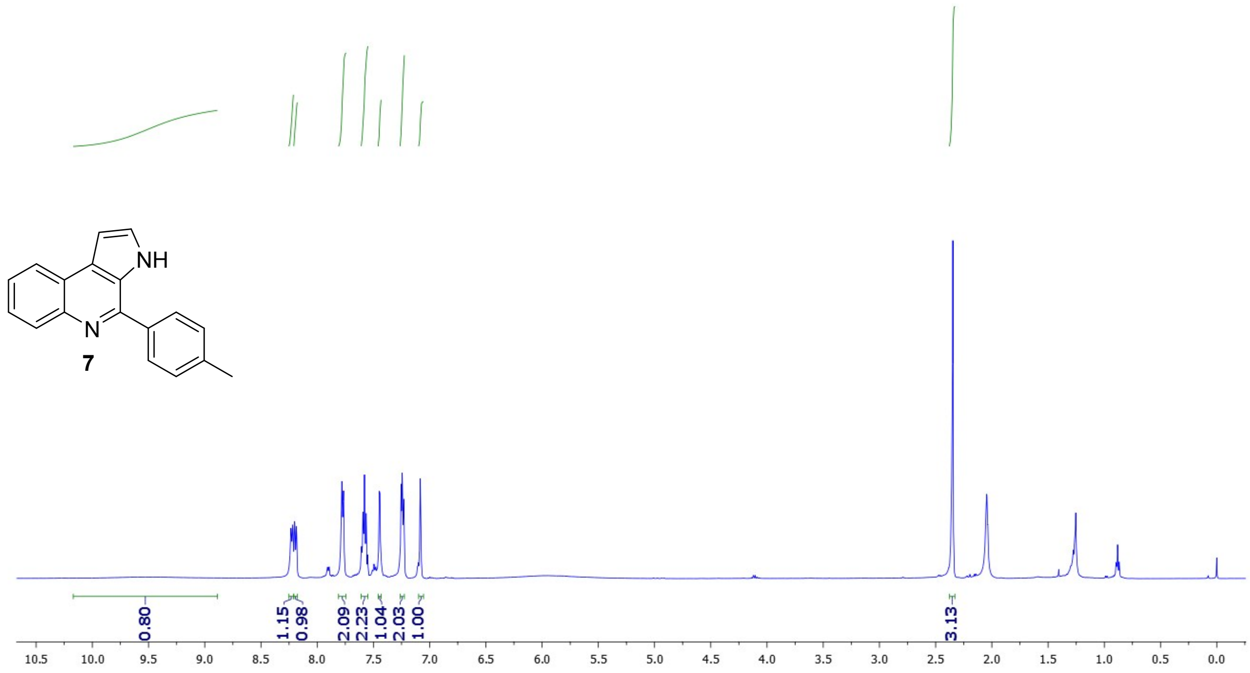
NH
N
7
0.80
1.15
0.98
2.09
2.23
1.04
2.03
1.00
3.13
10.5 10.0 9.5 9.0 8.5 8.0 7.5 7.0 6.5 6.0 5.5 5.0 4.5 4.0 3.5 3.0 2.5 2.0 1.5 1.0 0.5 0.0

^{13}C-NMR of 7:

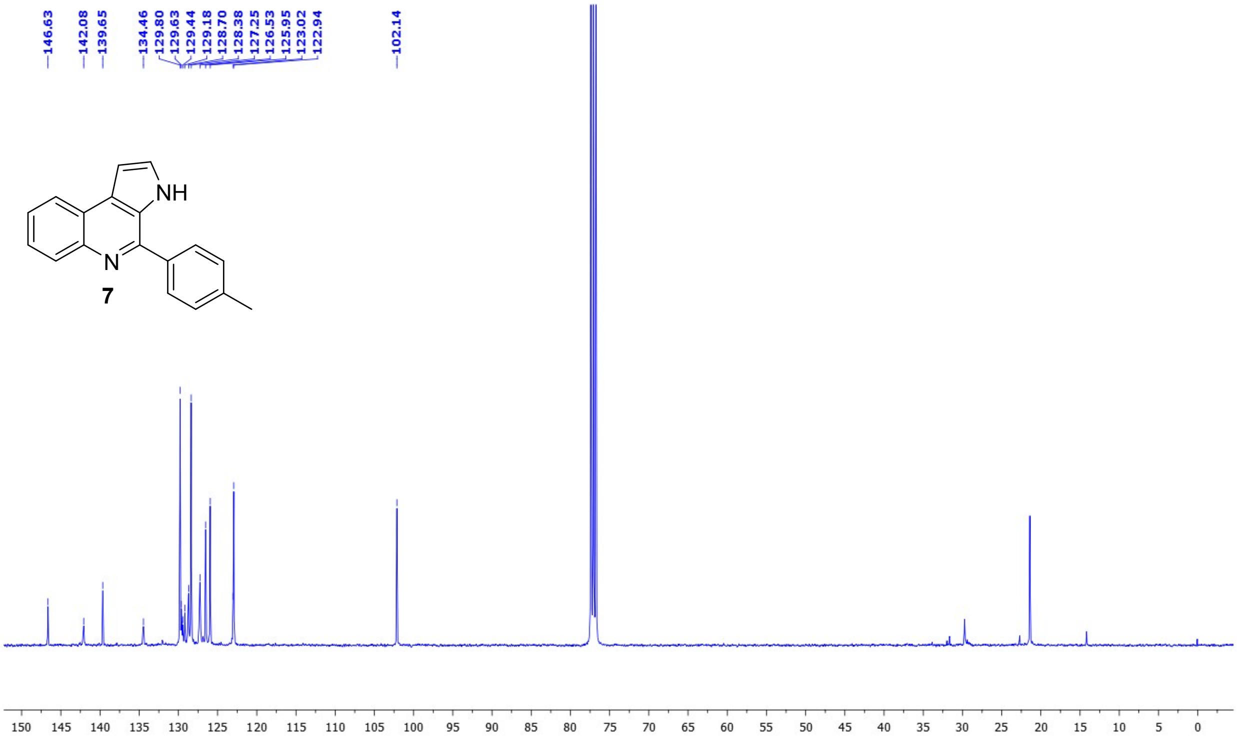
146.63
142.08
139.65
134.46
129.80
129.63
129.44
129.18
128.70
128.38
127.25
126.53
125.95
123.02
122.94
102.14
NH
N
7
150 145 140 135 130 125 120 115 110 105 100 95 90 85 80 75 70 65 60 55 50 45 40 35 30 25 20 15 10 5 0

^{1}H-NMR of 8:

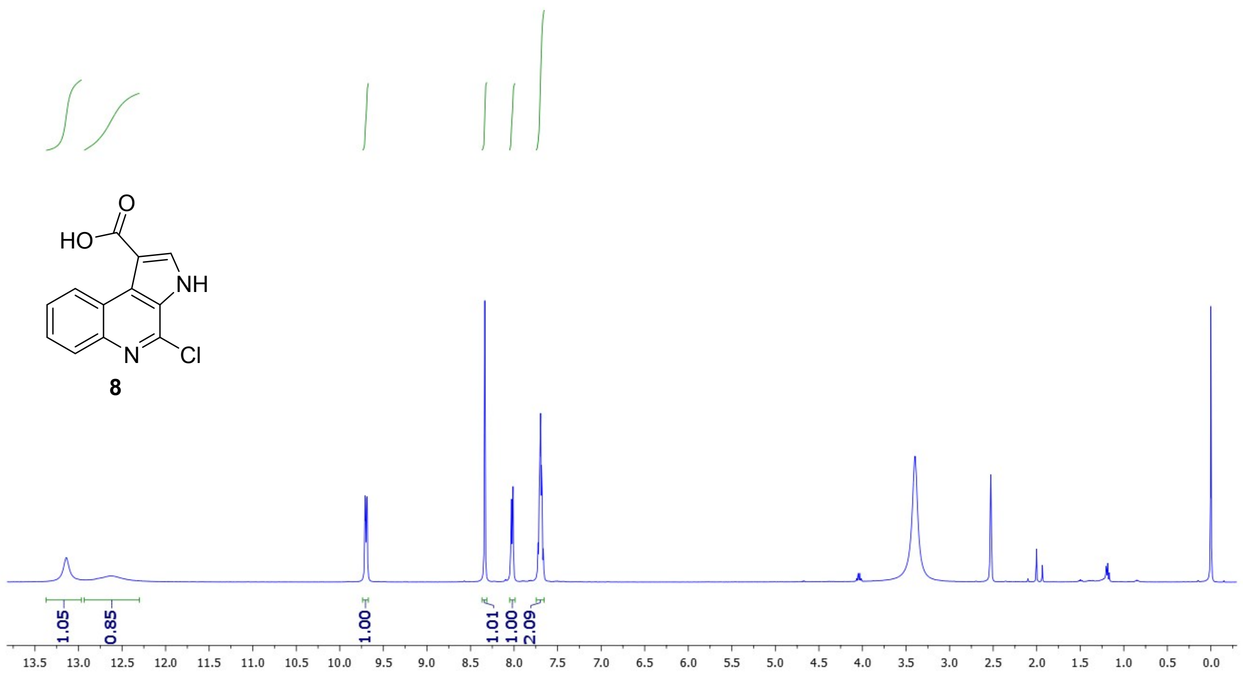

^{13}C-NMR of 8:

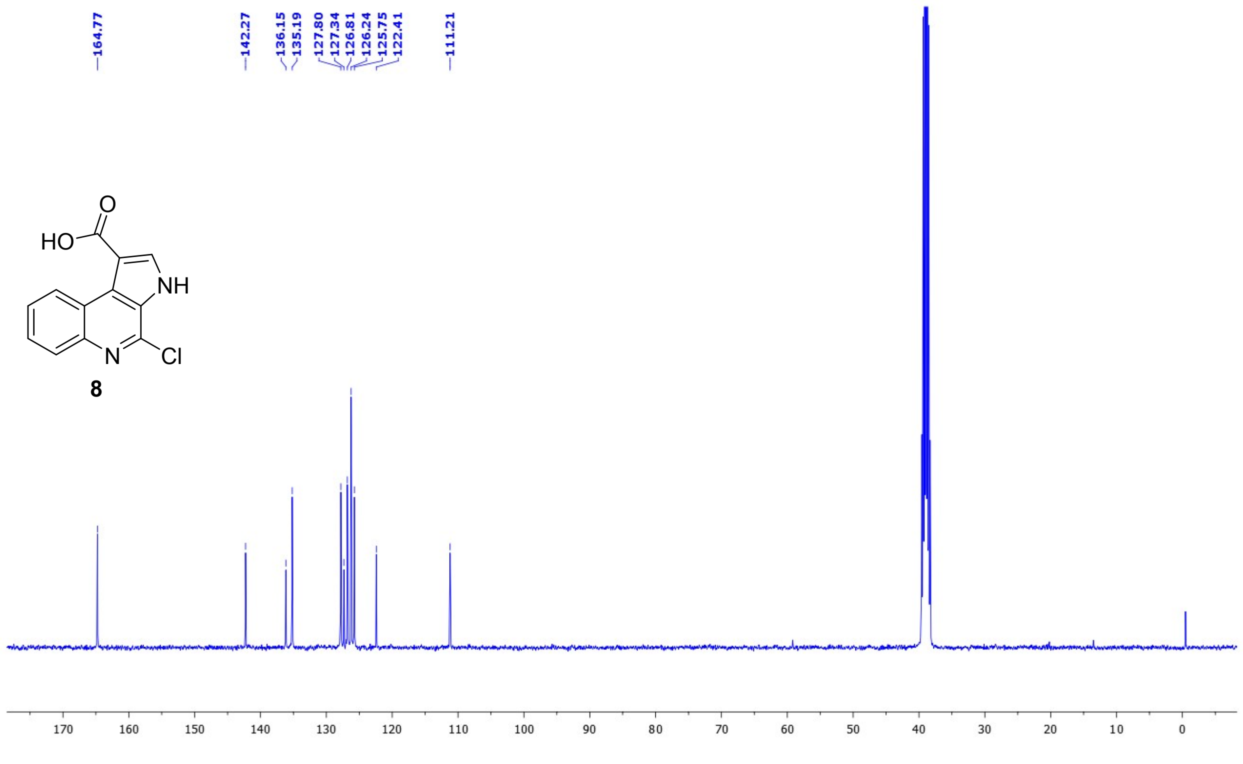

X-ray Crystallographic Data of compounds **3aa.**

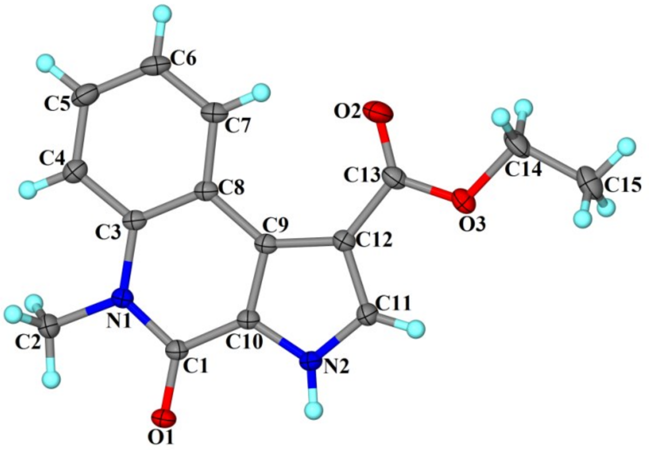

Figure caption: The molecular structure of **3aa** (in-house code # KA279) with the atom-numbering scheme. Displacement ellipsoids are drawn at the 30% probability level and H atoms are shown as small spheres of arbitrary radius. CCDC **1831087** contains the supplementary crystallographic data for this paper. These data can be obtained free of charge from the Cambridge Crystallographic Data Centre via https://www.ccdc.cam.ac.uk/structures/.

Table 1 Crystal data and structure refinement for KA279MF.

Identification code	KA279MF , CCDC **1831087**
Empirical formula	$C_{15}H_{14}N_2O_3$
Formula weight	270.28
Temperature/K	293(2)
Crystal system	triclinic
Space group	$P\bar{1}$
a/Å	6.063(4)
b/Å	9.023(7)
c/Å	11.915(8)
α/°	96.04(3)
β/°	90.884(19)
γ/°	90.83(2)
Volume/Å^3	648.1(8)
Z	2
ρ_{calc}g/cm^3	1.385
μ/mm^{-1}	0.098
F(000)	284.0
Crystal size/mm^3	0.380 × 0.210 × 0.120
Radiation	MoKα (λ = 0.71073)
2Θ range for data collection/°	4.54 to 51.988
Index ranges	-7 ≤ h ≤ 7, -11 ≤ k ≤ 11, -14 ≤ l ≤ 14

Reflections collected	8651
Independent reflections	2526 [R_{int} = 0.0397, R_{sigma} = 0.0403]
Data/restraints/parameters	2526/0/188
Goodness-of-fit on F^2	1.019
Final R indexes [I>=2σ (I)]	R_1 = 0.0406, wR_2 = 0.1299
Final R indexes [all data]	R_1 = 0.0524, wR_2 = 0.1521
Largest diff. peak/hole / e Å^{-3}	0.24/-0.17

Data collection and structure solution of 3aa (KA279): Single crystal X-ray data for two compounds were collected at room temperature on a Bruker D8 QUEST equipped with a four circle kappa diffractometer and Photon 100 detector. An Iμs microfocus Mo source (λ=0.71073Å) supplied the multi-mirror monochromated incident beam. A combination of Phi and Omega scans were used to collect the necessary data. Unit cell dimensions were determined using 5997 reflections for KA279. Integration and scaling of intensity data were accomplished using SAINT program.[1] The structures were solved by Direct Methods using SHELXS97[2] and refinement was carried out by full-matrix least-squares technique using SHELXL-2014/7.[2-3] Anisotropic displacement parameters were included for all non-hydrogen atoms. All H atoms were positioned geometrically and treated as riding on their parent C atoms with C-H distances of 0.93--0.97 Å, and with $U_{iso}(H) = 1.2U_{eq}$ (C) or $1.5U_{eq}$ for methyl atoms.

References:

1. SMART & SAINT. Software Reference manuals. Versions 6.28a & 5.625, Bruker Analytical X-ray Systems Inc., Madison, Wisconsin, U.S.A., 2001.
2. Sheldrick, G. M. SHELXS97 and SHELXL Version 2014/7, http://shelx.uni-ac.gwdg.de/SHELX/index.php.
3. Muller, P, Herbst-Imer, R, Spek, A. L, Schneider, T. R, and Sawaya, M. R. Crystal Structure Refinement: A Crystallographer's Guide to SHELXL. Muller, P. Ed. 2006 Oxford University Press: Oxford, New York, 57–91.
